# PEPITEM Regulates the Synovial Microenvironment During Immune‐Mediated Inflammatory Arthritis to Limit Disease

**DOI:** 10.1002/art.70108

**Published:** 2026-04-13

**Authors:** Mussarat Wahid, Samuel Kemble, Oladimeji Abudu, Anella Saviano, Christopher Mahony, Jonathan W. Lewis, Thomas A. Nicholson, Anna Schettino, Noemi Marigliano, Kathryn Frost, Jenefa Begum, Alyssa M. Urbanowski, Marion Limo, Rakesh Jha, Sandra Martinez Jarquin, Laleh Pezhman, Abbie E. A. Degan, Amy E. Anderson, Charlotte G. Smith, Armaiti Batki, Holly Adams, Francesco Caso, Raffaele Scarpa, Iain McInnes, Stefan Siebert, Arthur G. Pratt, Andrew Filer, Karim Raza, Adam P. Croft, Myriam Chimen, Felicity de Cogan, G. Ed Rainger, Asif J. Iqbal, Francesco Maione, Helen M. McGettrick

**Affiliations:** ^1^ Rheumatology Research Group, Department of Inflammation and Ageing University of Birmingham Birmingham UK; ^2^ ImmunoPharmaLab, Department of Pharmacy, School of Medicine and Surgery University of Naples Federico II Naples Italy; ^3^ Department of Cardiovascular Sciences University of Birmingham Birmingham UK; ^4^ Nanoscale and Microscale Research Centre University of Nottingham Nottingham UK; ^5^ Translational and Clinical Research Institute Newcastle University Newcastle Upon Tyne UK; ^6^ NIHR Newcastle Biomedical Research Centre Newcastle Upon Tyne Hospitals, NHS Foundation Trust and Newcastle University Newcastle Upon Tyne UK; ^7^ Rheumatology Unit, Department of Clinical Medicine and Surgery University of Naples Federico II Naples Italy; ^8^ School of Infection & Immunity, College of Medical, Veterinary and Life Sciences University of Glasgow Glasgow UK; ^9^ NIHR Birmingham Biomedical Research Centre University of Birmingham Birmingham UK; ^10^ Department of Rheumatology Sandwell and West Birmingham, NHS Trust Birmingham UK

## Abstract

**Objective:**

Here we investigate the status of the adiponectin–PEPITEM pathway in early, treatment naive rheumatoid arthritis (RA) and psoriatic arthritis (PsA) and the therapeutic efficacy of PEPITEM administration in preclinical models.

**Methods:**

Peripheral blood was isolated from patients with clinical suspect arthralgia and suspected inflammatory arthritis and analyzed by flow cytometry or Western blot. Effect of PEPITEM treatment on inflammatory arthritis was assessed in mice by histology, single‐cell RNA sequencing, flow cytometry, or multiplex analysis.

**Results:**

Patients newly diagnosed with RA and PsA had significantly reduced expression of adiponectin receptor 2 and its downstream signaling adapter protein APPL‐1 on their peripheral‐blood mononuclear cells, resulting in diminished response to adiponectin and local synovial concentrations of PEPITEM. Building on these observations, treatment with PEPITEM in three distinct inflammatory arthritis animal models significantly reduced arthritis severity, joint swelling, leukocyte infiltration, and expression of several pro‐inflammatory mediators (eg, JE [CCL2], RANTES, interleukin‐16) in the synovium. Mechanistically, PEPITEM treatment suppressed the cyclooxygenase 2 and NF‐κB signaling pathways. Moreover, PEPITEM altered the composition of leukocyte subsets recruited into the joint.

**Conclusion:**

Collectively, these findings underscore the importance of understanding the dysregulation of the adiponectin–PEPITEM pathway in different immune‐mediated inflammatory diseases (IMIDs), such as RA and PsA. The observed differences in expression and downstream signaling through adiponectin receptors suggest potential targets for therapeutic intervention to restore the balance of this regulatory pathway to mitigate chronic inflammation and disease progression in these patients, paving the way for its clinical use as an alternative and/or combination therapy for early IMIDs.

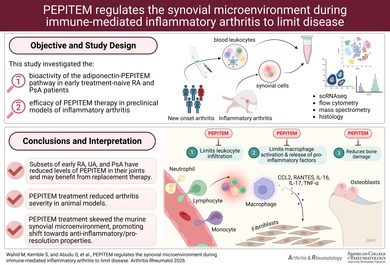

## Graphical abstract

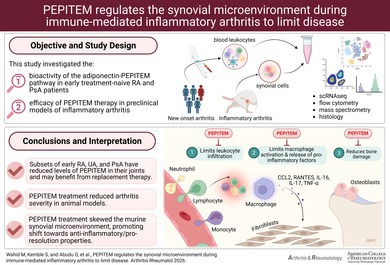

## INTRODUCTION

The inappropriate accumulation and activation of leukocytes is a shared pathologic feature of all immune‐mediated inflammatory diseases (IMIDs) and autoinflammatory diseases, including several arthropathies, such as, rheumatoid arthritis (RA),^1^ psoriatic arthritis (PsA),^2^ and gout, and is therefore an attractive target for therapeutic intervention.^3^, ^4^ However, attempts to target leukocyte accumulation within the joint by modulating their recruitment and retention, for example with chemokine receptor inhibitors, have not been successful to date.^5^ Although the current suite of biologic or targeted synthetic disease‐modifying antirheumatic drugs (b/tsDMARDs) targets pathogenic leukocyte populations and/or their cytokines, providing some patient benefit, there is still no cure for RA or PsA. Crucially, no current treatments aim to restore endogenous regulatory pathways. PEPITEM is an immunopeptide responsible for limiting leukocyte trafficking into sites of acute inflammation.^6^ Levels of PEPITEM are diminished in patients with some IMIDs (eg, type‐1 diabetes [T1D]^6^), but importantly, supplementation can limit migration of patient leukocytes ex vivo and disease severity in several murine models of IMIDs (eg, systemic lupus erythematosus [SLE],^7^ Sjögren disease,^6^ obesity,^8^ psoriasis^9^). This offers an alternative potential therapeutic avenue in the clinical management of IMIDs through restoration of homeostatic regulatory pathways.

PEPITEM is a 14 amino acid proteolytic cleavage product of the 14‐3‐3ζδ released from B cells in response to adiponectin.^6^ Patients with RA have elevated levels of adiponectin in plasma^10^, ^11^ and synovial fluid,^12^, ^13^ and these findings correlate with radiographic erosions,^12^, ^13^ suggesting a role for adiponectin in disease pathology. However, preclinical animal studies of adiponectin have been inconclusive, reporting both exacerbation^14^ and resolution^15^, ^16^ of arthritis following adiponectin therapy. Furthermore, adiponectin exists in different isoforms that may have divergent actions,^17^, ^18^ making it challenging to target therapeutically. We have previously shown that B cells from patients with established RA and T1D fail to respond to adiponectin, secreting less PEPITEM, and are unable to suppress effector memory T cell migration ex vivo.^6^ Given this, there is a distinct possibility that patients with RA may benefit from PEPITEM replacement therapy. Herein, we have explored the changes in the status of the adiponectin–PEPITEM pathway in patients across the different phases of disease from clinically suspect arthralgia (CSA) to newly diagnosed treatment naive RA and established PsA. Furthermore, we investigated the therapeutic efficacy and mechanism of action of PEPITEM to limit disease severity and modify the composition of the synovial microenvironment in multiple murine models of inflammatory arthritis. This approach provides valuable insights into the potential benefits of PEPITEM replacement (or in combination) therapy for patients with RA, along with a deeper understanding of its therapeutic mechanisms in modulating inflammation and disease progression in inflammatory arthritis.

## PATIENTS AND METHODS

### Human samples

Whole blood was obtained from consenting patients >18 years of age with suspected inflammatory arthritis seen in the Birmingham Early Arthritis Cohort (BEACON; Sandwell and West Birmingham NHS Trust and University Hospitals Birmingham NHS Foundation Trust) or the Newcastle Early Arthritis Clinic, UK (NEAC; Newcastle upon Tyne NHS Foundation Trust) or age‐ and biologic sex–matched healthy donors obtained from the 1000 Elders Cohort at the University of Birmingham between 2017 and 2024. All participants were DMARD and glucocorticoid naive at the time of enrollment (topical or inhaled glucocorticoids permitted). Patients were classified based on established criteria: the 2010 American College of Rheumatology (ACR)/EULAR^19^ or 1987 ACR classification for RA^20^; or Classification Criteria for Psoriatic Arthritis (CASPAR) Criteria for PsA.^21^ Patients with SLE, gout, spondyloarthritis, reactive arthritis (as defined^22^, ^23^, ^24^, ^25^), septic arthritis, pseudo‐gout, and sarcoidosis (classified based on clinical diagnosis) were excluded from the study. CSA was defined as the presence of symptoms that, in the opinion of a consultant rheumatologist, were suggestive of inflammatory arthralgia at risk of the development of RA (eg, prolonged morning stiffness, peripheral joint pain, a history of previous joint swelling) in the absence of clinically apparent joint swelling.^26^ All other patients were considered to have unclassified arthritis (UA). Demographic and clinical data related to the cohort are shown in Supplementary Table 1.

### Isolation of peripheral blood mononuclear cells

Peripheral blood mononuclear cells (PBMCs) were isolated from whole blood collected into EDTA‐coated VACUETTEs by centrifugation using a two‐step Histopaque (Sigma‐Aldrich) density gradient, as previously described.^27^ The PBMC pellet was resuspended in fetal bovine serum (FBS; F7524‐500ML, Sigma‐Aldrich) supplemented with 10% dimethyl sulfoxide and stored at −80°C until use. In some cases, PBMCs were treated with adiponectin (10 μg/mL; Enzo Life Sciences) for 15 minutes before messenger RNA (mRNA) extraction.^28^ Alternatively, naive T cells or CD4^+^CD127^low^CD49d^−^ T cells were purified using negative selection and treated with PEPITEM (15 ng/mL, unless otherwise stated; Cambridge Research Biochemicals Limited) immediately before polarization toward Th1 or Treg cells or addition to the migration assay.

### Migration assay

Human umbilical cord endothelial cells (HUVEC) were seeded as previously described^6^ and cultured overnight at 37°C, 5% CO_2_. HUVEC were stimulated with 100 U/mL tumor necrosis factor α (TNFα; R&D Systems) and 10 ng/mL interferon‐γ (PeproTech) for 24 hours before washing. PBMCs were isolated as described, and Treg cells were enriched using EasySep Human CD4^+^CD127^low^CD49^−^ Regulatory T cell Enrichment Kit according to manufacturer's protocol (19232, STEMCELL Technologies). Treg cells were either treated with vehicle (phosphate‐buffered saline [PBS]) or 15 ng/mL PEPITEM (Cambridge Research Biochemicals Limited, UK) immediately before the assay. Treg cells were allowed to adhere and migrate across the inflamed endothelial for seven minutes before removal of nonadherent cells by gentle washing with M199 supplemented with 0.15% bovine serum albumin (BSA). Treg cell behavior was assessed by acquiring images using phase‐contrast Olympus IX70 microscope (Olympus), which were analyzed offline using Image‐Pro 6.2 software (Media Cybernetics) as previously described.^8^ Treg cell transendothelial migration was calculated as a percentage of the total number of adhered cells and normalized to the vehicle control.

### T cell polarization

PBMCs were isolated as described, and CD4^+^ T cells were then enriched using EasySep Human Naive CD4^+^ T cell Isolation Kit II according to manufacturer's protocol (17555, STEMCELL Technologies) using Easy 50 EasySep Magnet, resuspended in Roswell Park Memorial Institute medium 1640 (RPMI1640) supplemented with 5% FBS, 50 U/mL penicillin, 50‐μg/mL streptomycin, and 2‐mM L‐glutamine (Sigma‐Aldrich) and cultured overnight as previously described. For T cell polarization, flat‐bottom 96‐well plates were coated with anti‐CD3 antibody (OKT3, 1 μg/mL, BioLegend) overnight at 4°C, before washing in PBS and addition of either ImmunoCult‐XF T Cell Expansion Medium (STEMCELL Technologies) or Treg cell polarization cocktail (1 μg/mL Ultra‐LEAF anti‐CD28 [CD28.2, BioLegend], 1 ng/mL transforming growth factor β1 [7754‐BH‐025/CF, R&D Systems], 20 U/mL interleukin‐2 [IL‐2; cat. # 111475208001 Roche]) alone or in combination with PEPITEM. Naive CD4^+^ T cells (8 × 10^4^ cells/well diluted in ImmunoCult‐XF T Cell Expansion Medium) were then cultured in wells for five days at 37°C in 5% CO_2_.

Polarization was assessed by flow cytometry. Cells were washed in fluorescence‐activated cell sorting (FACS) buffer (PBS containing 1% BSA) and stained for 20 minutes at 4°C with extracellular antibodies: anti‐CD25‐efluor 450 (BC96, 1:100), anti‐CD3‐PE‐Cy7 (OTK3, 1:100, both from Invitrogen), anti‐CD4‐Alexafluor 488 (SK3, 1:100), and Zombie Aqua (423101, 1:500, both from BioLegend). Following washing, cells were fixed and permeabilized using the Foxp3/Transcription Factor Staining Buffer Set (00‐5523‐00, eBioscience) and stained intracellularly with the following antibodies: anti‐Foxp3‐PE (259D, 1:50) and Ki67‐BV711 (350516, 1:100, both from BioLegend). A minimum of 1 × 10^4^ cells per sample were acquired on an LSRFortessa (BD Biosciences), and data was analyzed using FlowJo software (version 10). Data were plotted as percentage of CD4 cells expressing CD25 and Foxp3 for Treg cells.

### Gene expression analysis

Total PBMC mRNA was extracted using the RNeasy Minikit (QIAGEN) according to the manufacturer's instructions. For expression of *YWHAZ*, PBMCs were cultured in RPMI with L‐glutamine at 37°C for 24 hours and then treated with or without adiponectin (10 μg/mL; Enzo Life Sciences) for 1 hour at room temperature in magnetic‐activated cell sorting (MACS) buffer before RNA extraction. mRNA was converted to complementary DNA (cDNA) using the iScript cDNA synthesis kit according to the manufacturer's instructions (Bio‐Rad Laboratories Ltd). Gene expression was assessed using Assay on Demand kits for FAM‐labeled *ADIPOR1*, *ADIPOR2*, and *YWHAZ* (Hs01122445) primers, with *β_2_M* or *18S* run as housekeeping gene (Applied Biosystems). Samples were amplified in at least duplicate using the Bio‐Rad CFX384 Real‐Time PCR machine and analyzed using the software package Bio‐Rad CFX manager 3.1 (both from Applied Biosystems). Data were expressed as relative expression units (2^−ΔCt^) relative to *β_2_M* or *18S*

### Western blot

Synovial tissue homogenates (40 μg of protein) were analyzed by sodium dodecyl sulfate–polyacrylamide gel electrophoresis (10% gel) as previously described^7^ for expression of cyclooxygenase 2 (COX‐2) and NF‐κB. The proteins were transferred to nitrocellulose membrane (0.2 μm nitrocellulose membrane, Trans‐Blot TurboTM, Transfer Pack, Bio‐Rad Laboratories, RRID: SCR_008426) in transfer buffer (25 mM Tris‐HCl pH 7.4 containing 192 mM glycine and 20% volume/volume methanol) at 400 mA for two hours. Membranes were incubated with nonfat dry milk (5% wt/v) in PBS‐T (PBS supplemented with 0.1% [v/v] Tween 20) or BSA (5% wt/v) in TBS‐T (Tris buffered saline [TBS] supplemented with 0.1% [v/v] Tween 20) for two hours at RT, before incubated with the following appropriately diluted primary antibodies overnight at 4°C: rabbit monoclonal anti–COX‐2 (1:1,000; D5H5, Cell Signaling Technology), rabbit monoclonal anti–NF‐kB (1:1,000; D14E12, Cell Signaling Technology), mouse monoclonal anti–beta‐actin (0.01 μg/mL; MAB8929, R&D Systems). After washing three times with PBS‐T, membranes were incubated with a 1:3,000 dilution of related horseradish peroxidase–conjugated secondary antibody for two hours at RT and finally washed three times with PBS‐T. Protein bands were detected by using the enhanced chemiluminescence method (Clarity Western ECL Substrate, Bio‐Rad Laboratories) and ImageQuant 400 GE HealthCare software (GE HealthCare). Bands were quantified using the GS 800 Calibrated Imaging Densitometer software (Bio‐Rad) and normalized to the loading control (β‐actin; Supplementary Figure 9).

Alternatively, human PBMCs were suspended in radioimmunoprecipitation assay (RIPA) buffer (cat: R0278; Sigma‐Aldrich) containing 1X cOmplete Mini Protease Inhibitor Cocktail (cat: 11836153001; Merck) for 30 minutes on ice under constant agitation. Cell lysates were then centrifuged (12,000 g for 20 minutes) and supernatants stored at −80°C until needed. Protein quantification was performed using the Pierce BSA Protein Assay (cat: 23225; Thermo Fisher Scientific). PBMC cell lysates (10 μg) or protein ladder (5 μL) were diluted 1:1 with 2x Laemmli sample buffer (Bio‐Rad) and incubated at 95°C for five minutes. Samples were loaded onto a 10% Criterion Tris‐HCL protein gel (cat: 3450009; Bio‐Rad) and electrophoresed at 100 V for 90 minutes. The proteins were transferred to PVDF midi membrane (0.2 mm; Bio‐Rad Laboratories, RRID: SCR_008426) at 25 V for 10 minutes. Membranes were incubated with nonfat dry milk (5% wt/v) in TBS‐T for one hour at 4°C under agitation, before incubation with rabbit monoclonal anti–APPL‐1 antibody (1:4,000; 12639‐1‐AP, Proteintech) overnight at 4°C. After washing three times with TBS‐T, membranes were incubated with a 1:10,000 dilution of anti‐rabbit secondary antibody (7074S, Cell Signaling Technology) for one hour at 4°C and finally washed for three times with TBS‐T. Protein bands were detected by using the enhanced chemiluminescence (as described above in the first paragraph of this section) and ChemiDoc MP Imaging System (Bio‐Rad). Membranes were then stripped with ReBlot Plus Western Blot Recycling Kit (Millipore) for 30 minutes, before restain with mouse anti–β‐actin (1:4,000, A1978, Sigma‐Aldrich) or anti‐mouse IgG (1:5,000, 7076, Cell Signaling Technology) as described above. Bands were quantified using the ImageJ (version 2.1.0) and normalized to the loading control (β‐actin; Supplementary Figure 10).

### Mass spectrometry of PEPITEM

PEPITEM calibrations (0–100 nM) were prepared using heavy PEPITEM (S‐[U‐^13^C_5_,^15^N‐Val]‐TEQGAELSNEER, Cambridge Research Biochemicals Limited) in 4% BSA solution in PBS. Peptides from serum and synovial fluid, along with the calibrations, were purified using C18 3.5‐μm (3.0 × 100 mm) solid phase extraction columns from Waters XBridge. Separation was achieved with column temperature of 50°C and mobile phase consisting of (A) 5 mM ammonium formate buffer, pH 3.0 + 0.1% v/v formic acid and (B) 0.1% v/v formic acid in acetonitrile. The flow rate was 0.5 mL/min, injection volume 5 μL, using the following gradient: 0.00–4.50 minutes, 95% B; 5.00–5.05 minutes, 5% B; 5.20–15.60 minutes, 95% B; and 5.60–10.00 minutes, 95% B. Following solid phase extraction, samples and calibrations were dried using a Uniequip Univapo 100 ECH speedVac. Dry samples and calibration standards were then resuspended using 50 μL of solvent 0.1% formic acid in 2% acetonitrile. Liquid chromatography mass spectrometry/mass spectrometry (LC‐MS/MS) analysis was conducted using a SCIEX QTRAP 6500 LC‐MS/MS instrument in positive ion mode. Quantification was performed using multiple reaction monitoring mode. PEPITEM double‐charged molecular ion *m/z* 774.90 was selected as the most stable and high intensity precursor ion, as previously described.^6^ The product (fragment) ion transition selected as the qualifier (confirmation peak) was *m/z* 747.200, and the transition selected as the quantifier was *m/z* 634.200, which had highest peak intensity in all calibration standards. After each sample measurement, an extensive wash method was conducted for the autosampler, injection port, tubing, and column to ensure no carryover in between measurements. The wash method included solvents neat acetonitrile, 95% H_2_O and 5% acetonitrile, and re‐equilibration in 95% solvent B and 5% solvent A.

### Co‐immunoprecipitation

Identification of potential PEPITEM binding partners was assessed by treating endothelium or peripheral blood lymphocytes with biotinylated PEPITEM (Cambridge Research Biochemicals Limited) before lysing the cells, eluting the proteins and analyzing by mass spectrometry, as previously described.^6^, ^29^

### In silico PEPITEM interactions

Protein Uniprot sequences for intercellular adhesion molecule 1 (ICAM‐1; P05362) ran alongside PEPITEM (SVTEQGAELSNEER) to predict PEPITEM‐potential binding partner interactions in AlphaFold2_multimer using Google Colab using an unpaired_paired multiple sequence alignment mode to predict both peptide–protein interactions and estimate binding potential. The predicted local distance difference test (pLDDT) value and error plot were used to select the models of peptide–protein interaction with the highest predicted confidence. These models were three‐dimensionally rendered in ChimeraX software to find location and confidence of pseudobonds between the two proteins.

### Animals

Eight‐week‐old, male CD1 (strain code: 022), C57Bl/6J (strain code: 027) from Charles River Laboratories, or DBA‐1 (strain code: 1OlaHsd from Envigo) wild type mice were purchased. Animals were maintained in a specific pathogen free facility, with free access to food and water through long spout bottles.^30^ Mice were housed in our animal facility for at least two weeks before experimentation using soft bedding and nesting materials.^30^ Environmental conditions were at 21 ± 2°C, 55% ± 10% relative humidity, and had a 12‐hour light–dark cycle.

### Antigen‐induced arthritis

C57BL/6 mice were immunized by subcutaneous (SC) injections with methylated BSA (mBSA, 10 μg, Sigma‐Aldrich) in complete Freund's adjuvant (CFA; Fisher Scientific).^31^ On day 21, monoarthritis was induced by intra‐articular (i.a.) injection of mBSA (100 μg) into the knee. Mice were treated subclinically from day 21 by intraperitoneal (i.p.) injection with either vehicle control PBS, PEPITEM (300 μg, SVTEQGAELSNEER‐PEG [352]‐Amide; Cambridge Research Biochemicals Limited), or 50 μg infliximab (anti‐TNFα). Joint thickness (mm) was measured by calipers daily for up to seven days. Data are expressed as a percentage change from baseline measurement taken on day 21 for each mouse or as area under the curve (AUC).

### Collagen‐induced arthritis

DBA‐1 mice were immunized subcutaneously with bovine collagen II (CII; 100 μg, gift from Prof Richard Williams, University of Oxford, UK) in CFA (1:1). On day 21, polyarthritis was induced by SC injection of CII (100 μg) in incomplete Freund's adjuvant. Mice were treated subclinically from day 21 or therapeutically at the onset of arthritis by i.p. injection with either vehicle control (PBS) or PEPITEM‐PEG (300 μg). Mice were monitored daily to assess clinical score (amount of arthritis) and welfare as described.^30^ Ankle and footpad thickness (mm) were measured by calipers daily for up to seven days. Data are expressed as a percentage change from baseline measurement taken on day 21 for each mouse or as AUC.

### Monosodium urate–induced acute gouty arthritis

Monosodium urate (MSU) crystals were prepared as previously described.^32^ Briefly, 800 mg of MSU (U2875, Sigma‐Aldrich) were dissolved in 155 mL of boiling milli‐Q water containing 5 mL of NaOH and adjusted the pH to 7.2. The solution was cooled gradually by stirring at room temperature. Thereafter, crystals were collected after centrifugation at 3,000*g* for five minutes at 4°C. The obtained crystals were washed twice with 100% ethanol, dried, autoclaved (180°C for two hours), and weighed under sterile conditions. Endotoxin‐free crystals (Limulus polyphemus lysate assay: <0.01 EU/10 mg) were resuspended in PBS by sonication and stored in a sterile environment until use. CD1 mice were injected i.a. with MSU crystals (200 μg/20 μL) or vehicle (PBS) into the right tibiotarsal joint (ankle) followed by i.a. injection with either vehicle control (PBS) or PEPITEM (3 μg/15 μL/mouse) 30 minutes later. Ankle thickness (mm) was measured by calipers daily for up to two days. Data are expressed as a change from baseline measurement for each mouse or as AUC.

### Micro–computed tomography of joint damage

Limbs were placed vertically into the SKYSCAN 1172 micro–computed tomography (CT) scanner (Bruker) and imaged every 0.45° as previously described.^33^ Images were reconstructed using NRecon 1.6.1.5 (Bruker). Cross‐sectional images were reconstructed using the Feldkamp algorithm. Cross‐sectional images and 3D images were generated using the CTAN version 1.12.391 software (Computed Tomography Analyzer; Bruker) before images being “despeckled” in CTAN and then converted to a 3D mesh using a marching cube algorithm in CTAN, before meshes were analyzed in MeshLab version 1.3.2393 (ISTI‐CNR). Images were blinded and scored by two independent researchers for degree of erosion (0 = normal, 1 = roughness, 2 = pitting, or 3 = full thickness holes) and the extent of damage (0 = none, 1 = a few small areas, 2 = multiple small‐ to medium‐sized areas, or 3 = multiple medium‐ to large‐sized areas). Data were expressed as the average linear score per mouse per treatment group.

### Histopathological staining

Tibial formalin‐fixed paraffin‐embedded sections were analyzed by immunohistochemistry for hematoxylin (Harris, acidified) and 1% aqueous eosin (both from Pioneer Research Chemicals Ltd), safranin fast green staining solution (Abbexa), or tartrate‐resistant acidic phosphatase as previously described^34^ or according to the manufacturer's protocol. Slides were mounted using DPX‐Mount (Sigma‐Aldrich). Tissues were imaged using the ZEISS Axio Scan.Z1 and analyzed using ZEISS ZEN software (blue edition). Each image was blinded and scored by two independent researchers for the degree of inflammation (0 = no infiltrate detected, 1 = modest leukocyte infiltration in the synovial tissue, no fluid leukocyte, 2 = moderate leukocyte infiltration in synovial tissue, or 3 = gross leukocyte infiltration in the synovial membrane) and/or erosion of cartilage (0 = no abnormalities detected, 1 = fibrillation of cartilage and/or mild structural damage, 2 = moderate fibrillation and loss of cartilage, or 3 = significant loss of cartilage) as previously described.^35^

### Synovial tissue digest

Mice were culled by cervical dislocation, and the lower limbs were dissected. To isolate proteins, synovial tissue was sonicated in RIPA buffer (89900, Thermo Fisher Scientific) on ice and then centrifuged (4500*g* at 4°C) for 10 minutes before storing at −80°C until use. For collagen‐induced arthritis (CIA) and antigen‐induced arthritis (AIA), isolated joints were enzymatically digested for 45 minutes at 37°C in collagenase D (0.1 g/mL, Roche) and DNase I (0.01 g/mL, Sigma‐Aldrich) diluted in RPMI1640 containing 2% fetal calf serum (Biosera) (step 1), before filtering using 100‐μm cell strainer (vicedeal). Subsequently, joints were subjected to a further incubation with an enzyme mixture containing 0.1 g/mL Collagenase Dispase (Roche) and 0.01‐g/mL DNase diluted in RPMI1640 (Sigma‐Aldrich) containing 2% FBS at 37°C for 30 minutes (step 2). Cells from step 2 were filtered as described and combined with those from step 1 and centrifuged at 400*g* for 5 minutes. Samples were incubated in the RBC lysis buffer (Sigma‐Aldrich: R7757) for 10 minutes, followed by centrifugation at 400*g* in PBS and then resuspension in MACS buffer (0.1 mM EDTA [Sigma‐Aldrich] and 0.6% BSA [Gibco] in PBS) before analysis.

For gouty arthritis, isolated joints were enzymatically digested for one hour at 37°C in collagenase clostridium histolyticum (1 mg/mL, C2139‐Sigma) and hyaluronidase (2.4 mg/mL, H3506‐Sigma) diluted in RPMI1640 (all from Sigma‐Aldrich) containing 10% FBS (yourSial‐FBS‐SA) before filtering using 70 μm nylon mesh (Becton Dickinson). Cells were centrifuged in PBS at 400*g* for 5 minutes, resuspended with FACS buffer (PBS containing 1% BSA and 0.02% NaN_2_), and counted by TC20 automated cell counter (Bio‐Rad) using Bio‐Rad's disposable slides and TC20 trypan blue dye (0.4% trypan blue dye weight/volume in 0.81% sodium chloride and 0.06% PBS).

### Flow cytometry and cell sorting

All murine samples were blocked with FcR blocker (BioLegend) for 15 minutes before staining with the following antibodies for 20 minutes at 4°C; samples were then washed at 400*g* for 5 minutes at 4°C and fixed with 2% formaldehyde for 15 minutes on ice: anti‐CD45 BV605 (clone: 104), anti‐CD11b BV650 (clone: M1/70), LY6C FITC (clone: HK1.4), F4/80 APC‐efluro (clone: BM8, all from BioLegend), anti‐CD64 PE (clone: X454‐5/7.1), anti‐CD11c PE‐Cy 7 (clone: NH418, both from eBiosciences), and LY6G APC (clone: 1A8, BD Pharmingen). For cell sorting, synovial cells were stained as described in the previous sentence with the following antibodies: anti‐CD45 BV605, anti‐CD64 PE, anti‐CD3 AF700, F4/80 BV605, LY6G FITC, anti‐CD11b BV786, and anti‐CD31 BV450. For gouty arthritis samples, synovial cells were stained with the following antibodies for one hour: anti‐CD45 PE (clone: 30‐F11), anti‐CD3 PE/Cy7 (clone: 17A2), Ly6G APC (clone: 1A8, all from BioLegend), and Ly6B.2 FITC (clone: 7/4, Invitrogen).^6^ In some cases, compensation controls were generated using cells isolated from the spleen. Immediately, before analysis, CountBright beads (Thermo Fisher Scientific) with either Zombie Aqua (BioLegend) or Fixable Viability Dye eFluor (eBioscience) were added, and samples were acquired using Fortessa‐X20 or BriCyte E6 flow cytometer (Mindray Bio‐Medical Electronics). Cells were sorted for CD45 expression using FACS Aria (BD Biosciences) and subjected to single‐cell RNA sequencing (scRNA‐Seq). Data were analyzed offline using FlowJo (version 10.2.6) using the flow cytometry gating strategy depicted in Supplementary Figure 1.

For human samples, thawed PBMCs were recovered and resuspended in MACs buffer (PBS supplemented with 0.5% EDTA and 0.6% BSA). PBMCs were blocked with human FcR blocker (Miltenyi Biotec) for 2 minutes before staining with rabbit anti‐adipoR1 polyclonal antibody (G‐001‐44) or rabbit anti‐adipoR2 polyclonal antibody (G‐001‐23, Phoenix Pharmaceuticals) for 30 minutes at 4°C. Following washing by centrifugation in PBS, the samples were stained with Zombie Aqua (1:200, 43102, BioLegend) and the following antibodies for 30 minutes at 4°C: goat anti‐rabbit AF488 secondary (A11034, Invitrogen), anti‐CD56 PE (clone: 5.1H11), anti‐CD14 APC (clone: HCD14, both from BioLegend), anti‐CD4 AF700 (clone: RPA.T4), anti‐CD8 E450 (clone: OKT8), anti‐CD3 PerCPCy5.5 (clone: OKT3), and anti‐CD19 PE‐Cy7 (clone: HIB19, all from Thermo Fisher Scientific). PBMCs were used as compensation controls. Before acquisition, the Fortessa‐X20 was quality controlled using Spherotech 8 peak beads (Thermo Fisher Scientific). Samples were filtered and acquired using Fortessa‐X20. Data were analyzed offline using FlowJo (version 10.2.6) using the flow cytometry gating strategy depicted in Supplementary Figure 2.

### scRNA‐Seq and quality control preprocessing

cDNA libraries from FACS‐sorted CD45^+^ synovial digested cells were prepared using Chromium Next GEM Single Cell 3′ Kit version 3.1 (4 rxns PN‐1000269) according to the manufacturer's instructions, with the aim to capture about 10,000 cells/10 μL for each biologic replicate. Libraries were sequenced using a NovaSeq 6000 (Genomics Birmingham). Sequenced reads were aligned to mm10 using Cell Ranger (version 7.0.0, 10x Genomics). Ambient RNA was removed using SoupX (version 1.6.2), and doublets removed using DoubletFinder (version 2.0.3). Further quality control was performed using Seurat (version 4.3.0) Package in RStudio (version 4.3.1, Posit Software) to exclude cells with >10% mitochondrial gene content and keep only cells with 200 to 7,000 total genes. Downstream data process was performed in Seurat using the following functions: NormalizeData(), FindVaribleFeatures(), RunPCA(), RunUMAP(), FindAllMarkers(), and FindMarkers(). GoTerm analysis was completed using gsfisher (version 0.2) and differential abundance using scProportionTest (version 0.0.0.9), and heatmaps were plotted with ComplexHeatmap (version 2.10.0). Full analysis code is available at chrismahony/Wahid‐et‐al.‐PEPITEM (github.com) and sequencing data at GEO ‐ GSE324735.

### Enzyme‐linked immunosorbent assay and enzyme‐linked immunosorbent assay spot

Following joint digestion, cell lysates were assessed for expression of TREM‐2 and CCL2 (samples diluted 1:5), RANTES, IL‐6 (R&D Quantikine kits), PGE_2_ (KGE004B; R&D Systems), IL‐16, TNFα (both from Abcam), PGD_2_ (E‐EL‐0066; Elabscience), 5‐HETE (MBS2602657), and 12‐HETE (MBS265806; both from MyBioSource) were measured in triplicate, as per the manufacturer's instructions using a Synergy HT plate reader or Multiskan GO Microplate Spectrophotometer with absorbance set at 405 nm. Protein concentrations were expressed as a pg/mL or ng/mL.

Alternatively, fluids obtained from joint homogenates were pooled from eight mice per group and analyzed in technical duplicate using a proteome profiler mouse cytokine array kit (ARY006, R&D Systems), according to the manufacturer's instructions. Proteins were detected using the enhanced chemiluminescence detection kit and GE HealthCare Image Quant 400 software (GE HealthCare) and quantified using the GS 800 imaging densitometer software (Bio‐Rad).

Serum from healthy controls (HC) and patients attending early arthritis cohorts was diluted 1:5,000 and adiponectin levels were quantified using human adiponectin/Acrp30 DuoSet ELISA (DY1605, R&D Systems) according to the manufacturer's guidelines, using a Synergy HT plate reader with absorbance set at 405 nm. Protein concentrations were expressed as a μg/mL.

### Statistical analysis

Data were analyzed using GraphPad Prism (GraphPad Software) and presented as mean ± SEM for n mice or patients per group across n independent experiments. Normality was assessed using Shapiro–Wilk test. Univariate analysis was performed using unpaired *t*‐test, paired *t*‐test, or Mann–Whitney test. Multivariate analysis was performed using analysis of variance (ANOVA) or Kruskal–Wallis, with Dunnett test, Bonferroni test, or Dunn posttest. *P* < 0.05 was deemed statistically significant.

### Ethics approval statement

The study was conducted in compliance with the Declaration of Helsinki. All human samples were obtained with written informed consent and approval from the Human Biomaterials Resource Centre (Birmingham, UK), West Midlands and Black Country Research Ethics Committee (07/H1203/57), Newcastle and North Tyneside 2 Research Ethics Committee (12/NE/0251), or University of Birmingham Local Ethical Review Committee.

Animal studies were regulated by the Animals (Scientific Procedures) Act 1986 of the United Kingdom or the EU Directive 2010/63/EU for animal experiments and performed under Personal Project License PP0156739 (CIA and AIA) or Italian Ethical Authorization 507/2022‐PR (MSU). Approval was granted by the University of Birmingham's Animal Welfare and Ethical Review Body or the Italian Ministry of Health, respectively. All ethical guidelines were adhered to while carrying out this study with adherence to the 3Rs concept, in accordance with the Italian D.L.No.116 of January 27, 1992 and associated guidelines in the European Communities Council (86/609/ECC and 2010/63/UE).

### Data availability statement

Data are available upon reasonable request. All data relevant to the study are included in the article, uploaded as supplementary information, or available from cited open access repositories.

## RESULTS

### Differential expression of the adiponectin receptor/signaling pathway by patients with and at risk of inflammatory arthritis

**Figure 1:**
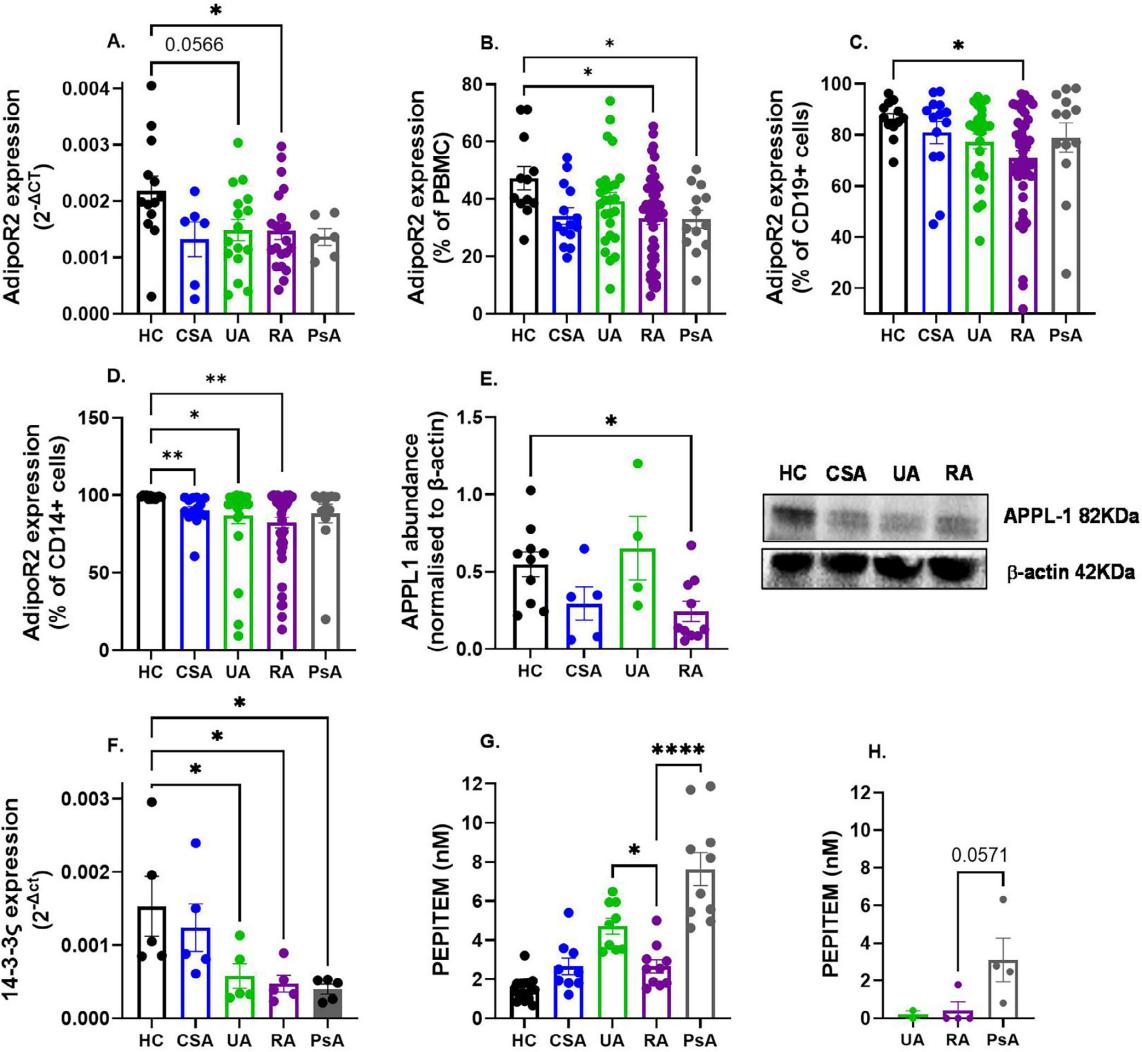
Patients with rheumatoid arthritis and PsA have a defect in the adiponectin–PEPITEM pathway. PBMCs were isolated from patients with CSA (n = 14), UA (n = 26), RA (n = 47), or PsA (n = 14) or age‐matched HC (n = 12). Adiponectin receptor 2 (A) gene or (B–D) protein expression was assessed on (A, B) PBMCs, (C) CD19^+^ B cells, or (D) monocytes by (A) qPCR or (B, D) flow cytometry. (A) Gene expression was expressed as 2^−ΔCT^ relative to the housekeeping gene *β*_*2*_ *M*. Protein expression was expressed as the percentage of (B) PBMCs or (C) B cells or (D) monocytes positive for adiponectin receptor staining. (A, B) ANOVA and (C, D) Kruskal–Wallis show a significant effect of patient group on adiponectin receptor 2 expression, *P* < 0.05. (E) Representative Western blot gel for APPL‐1 expression quantified relative to β‐actin loading control for n = 10 (HC), n = 5 (CSA), n = 4 (UA), and n = 8 (RA). (F) *14‐3‐3ζ* gene expression expressed as 2^−ΔCT^ relative to *18S*: n = 5 per group. ANOVA shows a significant effect of patient group on (E) APPL‐1 and (F) *14‐3‐3ζ* gene expression, *P* < 0.05. (G) Plasma and (H) synovial fluid PEPITEM concentrations expressed as μM for (G) n = 9–10 per group, with n = 17 for HC, and (H) n = 2–4 per group. Data are mean ± SEM. *P < 0.05, **P < 0.01, and ****P < 0.0001 by (A, B, E, F) Dunnett, (C, D) Dunn's posttest, and (H) Mann‐U. AdipoR, adiponectin receptor; ANOVA, analysis of variance; CSA, clinically suspect arthralgia; CT, computed tomography; HC, healthy controls; PBMC, peripheral blood mononuclear cell; PsA, psoriatic arthritis; qPCR, quantitative polymerase chain reaction; RA, early rheumatoid arthritis; UA, unclassified arthritis.

Dysregulation of the adiponectin–PEPITEM pathway in PBMCs from patients with T1D or aged donors (>65 years) has been previously associated with reductions in the expression of and/or the downstream signaling from adiponectin receptors.^6^ Initially, we assessed whether patients presenting with early inflammatory arthritis (typically less than six months symptom duration—Supplementary Table 1) exhibited alterations in the expression of adiponectin receptors. All patient groups showed reduced levels of *ADIPOR1* and *ADIPOR2* transcript when compared to age‐matched HC (Figure 1A; Supplementary Figure 3A). However, transcript levels were only significantly diminished in newly diagnosed treatment naive patients with RA for both genes and in patients with PsA for *ADIPOR1*, with borderline reductions in *ADIPOR2* transcript also seen in the UA group (Figure 1A; Supplementary Figure 3A). We observed considerable variability in the percentage of PBMCs and B cells from newly diagnosed treatment naive patients with RA, and to a lesser extent patients with UA, expressing ADIPOR1 on their surface when compared to HC (Supplementary Figure 3B, C) ‐albeit these changes were not statistically significant. Of the other leukocyte populations known to express the adiponectin receptors, there was no difference in the expression of ADIPOR1 on monocytes (Supplementary Figure 3D) or natural killer–cells (data not shown) across the patient groups. By contrast, frequency of PBMCs expressing ADIPOR2 was significantly reduced in the newly diagnosed RA and PsA groups (Figure 1B), with this reduction translating to a significant reduction in the frequency of RA B cells and monocytes expressing ADIPOR2 (Figure 1C, D).

To examine this further, we assessed the signaling machinery downstream of the adiponectin receptors in PBMCs by measuring levels of APPL‐1 in untreated cells by Western blot and the transcription of 14‐3‐3κ gene (*YWHAZ*—the parent protein for PEPITEM) following one hour of adiponectin stimulation. Patients newly diagnosed with RA had significantly lower levels of APPL‐1 protein and *14‐3‐3ζ* gene expression compared to HC (Figure 1E, F), indicating reduced ability to signal downstream of the adiponectin receptors, leading to diminished ability to produce the immunopeptide PEPITEM. Likewise, patients with UA or PsA also exhibited a significant reduction in *14‐3‐3ζ* induction following adiponectin treatment of PBMCs (Figure 1F). Similar observations are seen in aged individuals, in which PBMCs exhibit reduced capacity to respond to adiponectin leading to enhanced migration across inflamed endothelium.^28^ Indeed, we observed that PBMCs from patients with RA were unable to respond to adiponectin, but inhibition of migration could be rescued by addition of exogenous PEPITEM.^6^ Circulating levels of adiponectin remained unchanged in HC and patients with CSA, UA, or PsA—although levels were elevated in the treatment naive RA group, this was highly variable and was not statistically different (Supplementary Figure 3E). Collectively, these findings indicate that a proportion of patients with RA, and to a lesser extent UA and PsA, exhibit a defect in the adiponectin–PEPITEM pathway that contributes to aberrant leukocyte infiltration into the joint.

### Bioavailability of circulating PEPITEM hampered in RA

Based on the findings in the previous section, we anticipated that the dysregulation of adiponectin signaling would lead to reduced circulating levels of PEPITEM in patients with inflammatory arthritis. Although PEPITEM concentrations were significantly lower in the serum from patients with RA compared to UA, these levels were surprisingly much higher than those seen in age‐matched healthy donors (Figure 1G). Circulating proteins/peptides can be sequestered by plasma proteins, thereby limiting their bioavailability. To assess this, we used a biotin‐conjugated PEPITEM to “fish” for potential binding partners that are known to exist in membrane and soluble forms using mass spectrometry. Comparative analysis allowed exclusion of molecules identified in both control peptide and PEPITEM samples, with top hits including ICAM‐1 and thrombospondin—both reported to be elevated in RA.^36^, ^37^, ^38^ Further analysis using AlphaFold2_Multimer neural network^39^ coupled with ChimeraX^40^ generated a predicted 3D model of PEPITEM binding/interacting with ICAM‐1 (Supplementary Figure 4A–D). Predicted model 1 indicates PEPITEM binding to the Ig‐like C2‐type 4 domain within the extracellular portion of ICAM‐1 (Supplementary Figure 4B–D).

We also created 3D models of PEPITEM interacting with the soluble form of ICAM‐1, observing higher predicted residue interactions compared to those obtained with the membrane‐bound ICAM‐1 protein (Supplementary Figure 4E). In this model, PEPITEM is interacting with the Ig‐like C2‐type 4 domain, with higher alignment scores (pLDDT score > 70) than seen when PEPITEM is modeled with full‐length ICAM‐1 (Supplementary Figure 4F–H). The combination of the predictive 3D models, serum PEPITEM, and reported soluble ICAM‐1 levels strongly suggest that serum PEPITEM is not bioavailable to mediate its immunoregulatory functions and that patients with a defect in adiponectin signaling could benefit from supplementation of PEPITEM to allow restoration of the adiponectin–PEPITEM pathway. To further support this, we detected very low amounts of PEPITEM within the synovial fluid from patients with RA compared to patients with PsA (Figure 1H), further suggesting diminished local functionality to modulate immune cell infiltration into the joint.

### PEPITEM reduces severity of inflammatory arthritis in animal models

**Figure 2:**
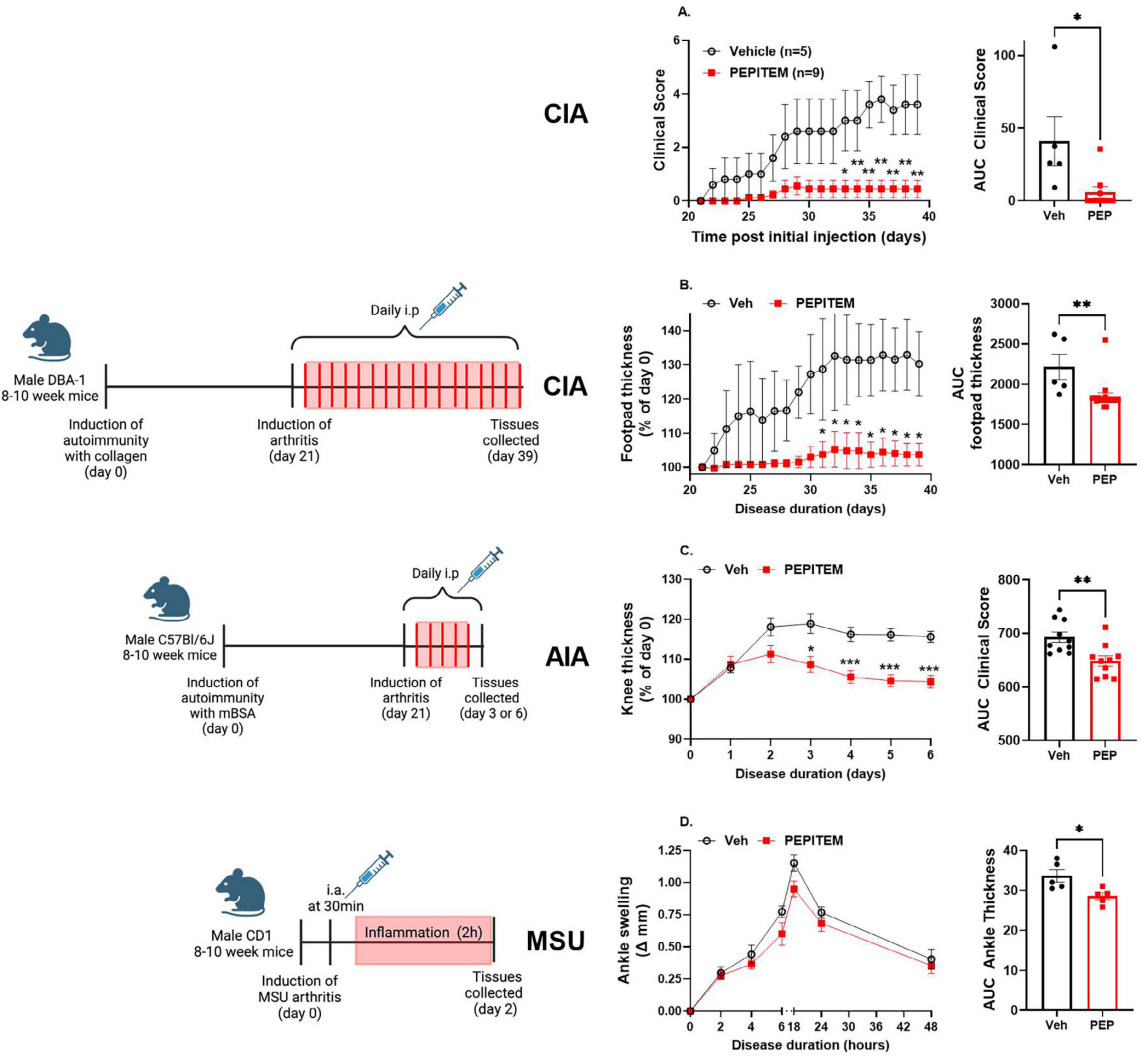
PEPITEM regulates the onset and severity of inflammatory arthritis. Mice with (A, B) CIA, (C) AIA, or (D) MSU arthritis were treated with Veh (black) or PEP (red) and assessed for clinical signs of arthritis—schematic images created in biorender.com. (A) CIA clinical score over time and expressed as AUC (n = 5–9). (B) CIA‐induced footpad, (C) AIA‐induced knee thickness (n = 14), or (D) MSU‐induced ankle thickness (n = 5) expressed as percentage change from baseline for each animal over time and AUC. In all, ANOVA shows a significant effect of treatment on clinical score or joint thickness, *P* < 0.05. Data are mean ± SEM from at least 2 independent experiments. *P < 0.05, **P < 0.01, and ***P < 0.001 by Bonferroni compared to Veh for the same time point or on AUC data unpaired *t*‐test. AIA, antigen‐induced arthritis; ANOVA, analysis of variance; AUC, area under the curve; CIA, collagen‐induced arthritis; i.a., intra‐articular; i.p., intraperitoneal; mBSA, methylated bovine serum albumin; MSU, monosodium urate; PEP, PEPITEM‐PEG; Veh, vehicle control.

**Figure 3:**
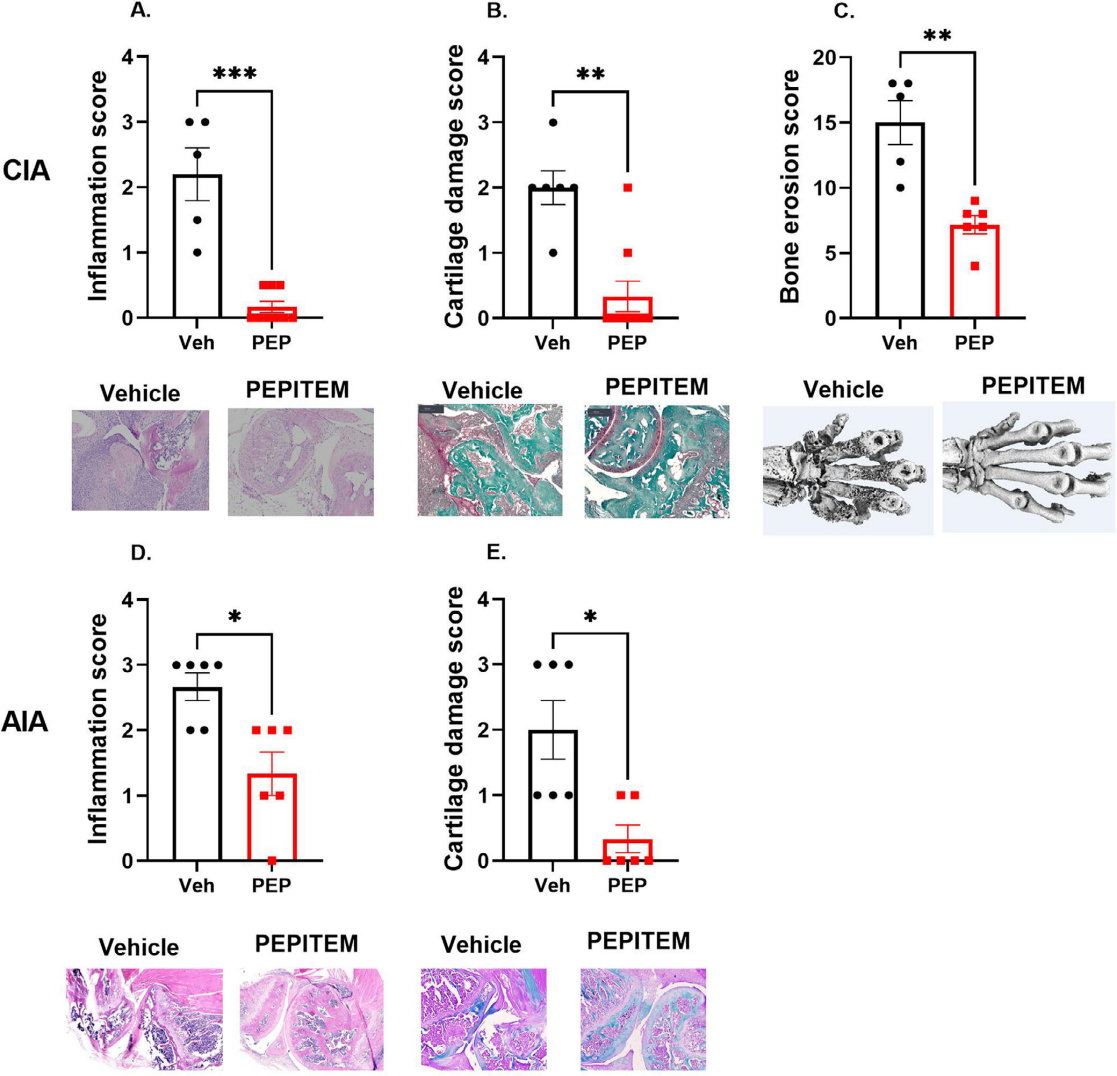
PEPITEM reduces joint inflammation and damage. Mice with (A–C) CIA or (D, E) AIA were treated with Veh (black) or PEP (red) before analysis of the synovial tissue by immunohistochemistry. (A, D) Degree of inflammation determined by H&E score. (B, E) Cartilage damage assessed by safranin O. (C) Bone erosion measured by micro‐CT. Data are expressed as a numerical score per parameter. Data are mean ± SEM for n = 5–6 mice per condition from at least 2 independent experiments. *P < 0.05, **P < 0.01, and ***P < 0.001 by Mann‐Whitney test or (C) unpaired *t*‐test. AIA, antigen‐induced arthritis; CIA, collagen‐induced arthritis; CT, computed tomography; H&E, hematoxylin and eosin; PEP, PEPITEM‐PEG; Veh, vehicle control.

Current therapeutics largely target pathogenic leukocytes or the cytokines they produce, that is, treating the existing inflammation to alleviate symptoms of the disease. Few/no drugs are currently available that restore endogenous regulatory mechanisms to switch the balance between inflammation and resolution. Here, we examined whether administration of the immunopeptide, PEPITEM, modulated disease onset and clinical severity using three different murine models of inflammatory arthritis representative of different arthropathies. Administration of synthetic PEPITEM before signs of disease onset prevented onset of CIA, significantly reducing disease incidence (with only two out of nine mice developing arthritis), clinical score, and footpad swelling (Figure 2A, B). Importantly, PEPITEM had no impact on autoantibody production in mice with CIA when compared to the untreated arthritic controls (Supplementary Figure 5A), confirming loss of tolerance had occurred before PEPITEM administration. Similar reductions in joint swelling were also observed when mice with monoarthritis (AIA) or gouty (MSU) arthritis were treated with PEPITEM before disease onset compared to control groups (Figure 2C, D). This reduction was comparable to the current standard of care anti‐TNFα therapy (infliximab) in the AIA model (Supplementary Figure 5B). As anticipated, these changes were mirrored within the synovial tissue, with significantly less joint inflammation, cartilage damage, and bone erosion observed in the PEPITEM‐treated mice compared to the vehicle controls for both the CIA and AIA models (Figure 3). Collectively, these data reveal the ability of PEPITEM to suppress arthritis onset and progression.

### PEPITEM limits leukocyte infiltration into inflamed joints

**Figure 4:**
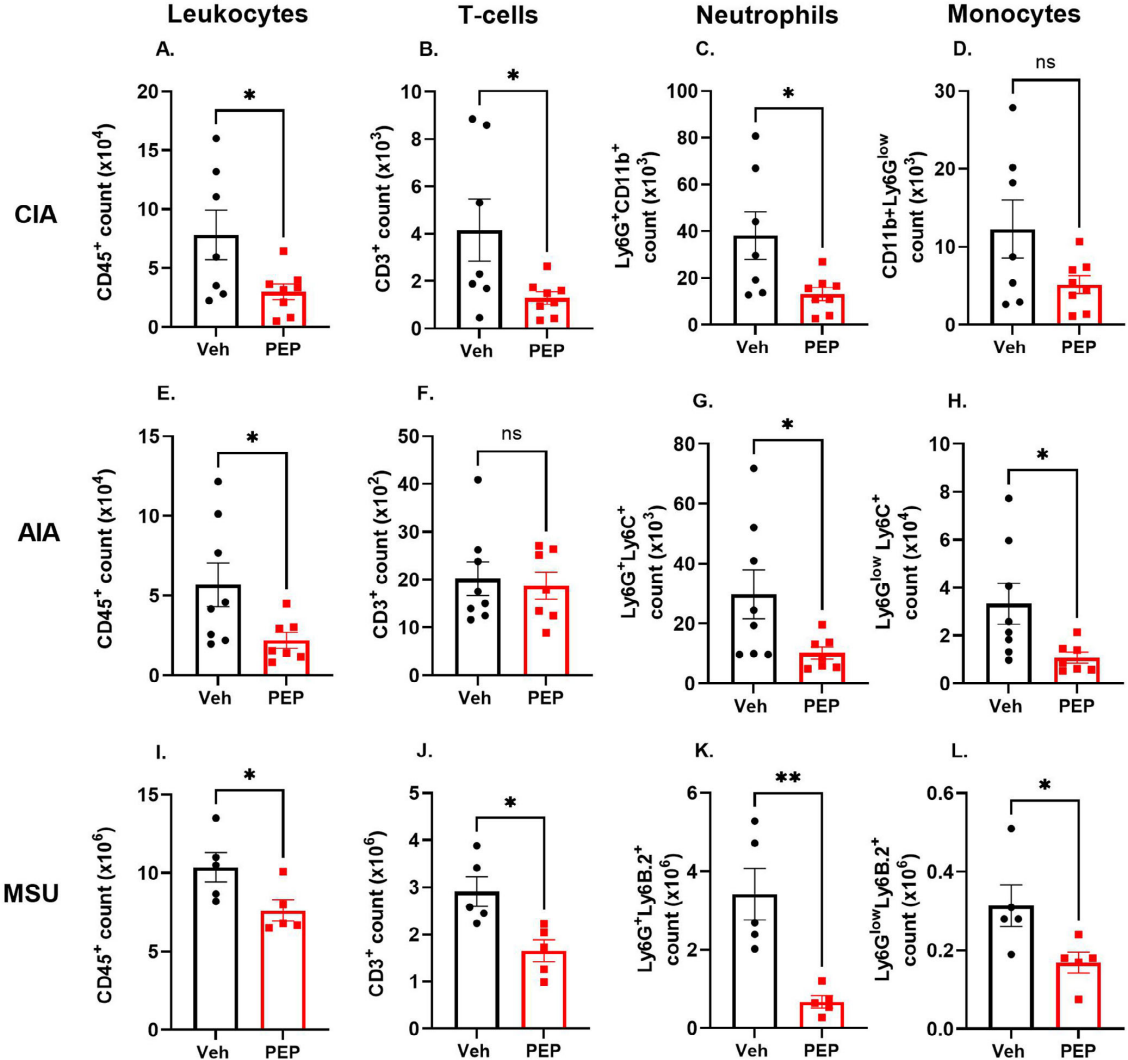
PEPITEM reduces immune cell infiltration. Mice with (A–D) CIA, (E–H) AIA, or (I–L) MSU arthritis treated with Veh (black) or PEP (red) before analysis of the synovial tissue by flow cytometry. Total number of (A, E, I) CD45^+^, (B, F, J) CD3^+^ T cells, (C, G, K) Ly6G^+^ neutrophils, and (D, H, L) Ly6G^−^ monocytes. Data are mean ± SEM for n = 5–8 mice per condition from at least 2 independent experiments. *P < 0.05 and **P < 0.01 by unpaired *t*‐test. AIA, antigen‐induced arthritis; CIA, collagen‐induced arthritis; MSU, monosodium urate; ns, not significant; PEP, PEPITEM; Veh, vehicle control.

PEPITEM regulates trafficking of T cells and monocytes/macrophages into tissues upon infection and ischemic/reperfusion injury^6^ and also in models of chronic human diseases, including obesity^8^ and SLE.^7^ We found that PEPITEM significantly reduces the number of CD45^+^ leukocytes infiltrating into the joints in all three arthritis models (Figure 4A, E, I). Specifically, we observed reduced numbers of CD3^+^ T cells in the joints from CIA and MSU arthritic mice (Figure 4B, J) but not in the AIA model (Figure 4F), in which reductions were seen in B220^+^ B cells (Supplementary Figure 5D). Although CIA and AIA are broadly driven by T cells, myeloid cells also play a significant role in pathology in both models and in MSU arthritis.^30^, ^32^ Accordingly, we also observed a significant reduction of Ly6G^+^ neutrophils and Ly6G^−/low^ monocytes in AIA (Figure 4G, H) and MSU arthritis (Figure 4K, L) but not in CIA (Figure 4C, D). Similar reductions were observed when infliximab was administered to mice with AIA.^31^ Of note, PEPITEM had no impact on B220^+^ B cell levels in the synovial joint or CD45^+^ leukocyte levels in neighboring draining lymph nodes in CIA when compared to the untreated control (Supplementary Figure 5C, E). Taken together, these data indicate that PEPITEM regulates leukocyte migration into peripherally inflamed tissues (eg, arthritic joint) without affecting trafficking through lymphoid tissues.

### PEPITEM alters the composition of the synovial microenvironment

**Figure 5:**
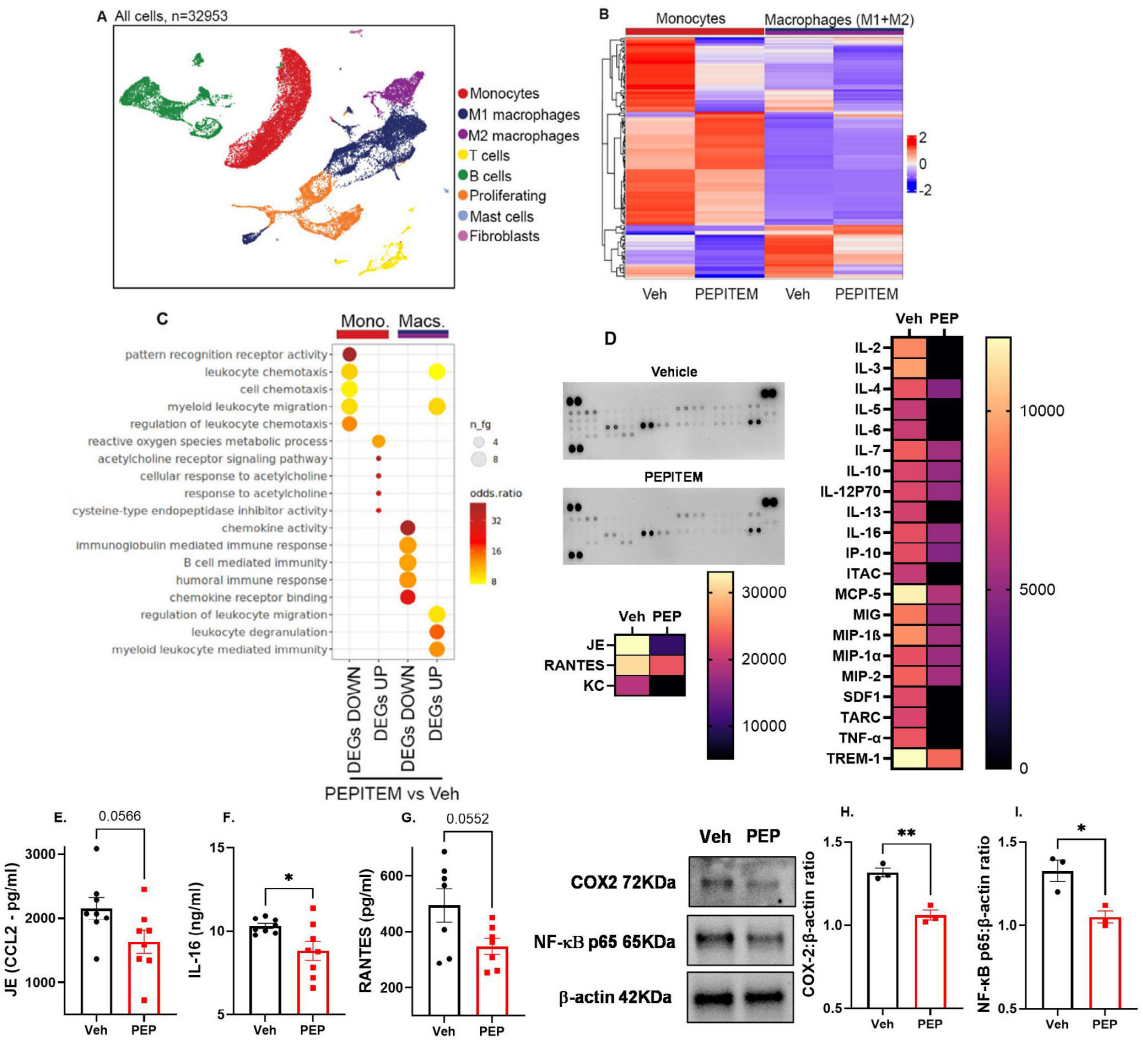
PEPITEM alters the inflammatory status of the synovial microenvironment. Synovial tissue from mice with AIA treated with Veh (black) or PEP (red) was collected at the peak of inflammation (day 3). (A–C) CD45^+^ cells were analyzed by scRNA‐Seq and (A) UMAP of leukocyte cells and cluster annotations. (B) Heatmap denoting the genes differentially regulated [adjusted *P* value < 0.05, calculated using Wilcoxon Rank Sum test Bonferroni correction in Seurat::FindMarkers()] following PEPITEM treatment in monocytes and macrophage populations compared to the vehicle. Color denotes the average scaled expression. (C) GO term pathway analysis of DEGs up‐ or down‐regulated by PEPITEM in either monocytes or macrophages. (D–I) After tissue digestion, the acellular fraction was analyzed by (D) multiplex ELISAspot, ELISA for (E) CCL2, (F) IL‐16, or (G) RANTES or Western blot for (H) COX‐2 or (I) NF‐κB and expressed as (D) heatmap (n = 2 technical replicates from 8 pooled mice per group), (E–G) ng/mL (n = 7–8 mice per condition), or (H, I) relative to β‐actin loading control (n = 3, where at least 4 mice were pooled per sample). Data are mean ± SEM from at least 2 independent experiments. *P < 0.05 and **P < 0.01 by unpaired *t*‐test. AIA, antigen‐induced arthritis; COX‐2, cyclooxygenase 2; DEG, differentially expressed gene; ELISA, enzyme‐linked immunosorbent assay; GO, gene ontology; IL‐16, interleukin‐16; Macs., macrophage; Mono., monocyte; n_fg, number of foreground genes; PEP, PEPITEM‐PEG; scRNA‐Seq, single‐cell RNA sequencing; UMAP, Uniform Manifold Approximation and Projection; Veh, vehicle control.

To ascertain the cellular and molecular mechanism underpinning the bioactivity of PEPITEM during inflammatory arthritis, we undertook scRNA‐Seq analysis on the CD45^+^ cellular component of the joints at the peak of inflammation in mice with AIA—as an exemplar model of autoimmunity and resolving inflammation involving multiple leukocyte subsets. After integrating our six samples (Supplementary Figure 6A), we then resolved the main cell types present (monocytes, macrophages, T cells, B cells, proliferating cells, mast cells, and fibroblasts) in the 32,953 cells that passed quality control based on the main marker gene expression (Figure 5A, Supplementary Figure 6B, Supplementary Table 2). Differential abundance analysis showed a significant decrease in the number of M2‐like regulatory macrophages, fibroblasts, and mast cells and an increase in proliferating cell numbers in the PEPITEM mice (Supplementary Figure 6C–D). Subsequent analysis revealed significant transcriptional differences in the monocyte and macrophage populations at the peak of inflammation in PEPITEM treatment when compared to vehicle controls expression (Figure 5B, Supplementary Table 2 and 3). Interestingly, PEPITEM treatment down‐regulated genes related to chemotaxis, leukocyte migration, and leukocyte function in these two populations (Figure 5C).

Further validating these findings, we observed substantial down‐regulation in the protein expression for 24 pro‐inflammatory cytokines and chemokines in the synovial tissue from mice treated with PEPITEM compared to control animals (Figure 5D). Of note, in depth analysis revealed less JE (CCL2), RANTES, IL‐16, and TREM‐1 proteins detected in mice treated with PEPITEM than in the control group, albeit TREM‐1 levels were borderline significant (Figure 5E–G, Supplementary Figure 7). By contrast, we observed no change in TNFα, IL‐6, or members of the prostaglandin family (PGD_2_, PGE_2_, 5‐HETE, 12‐HETE) upon PEPITEM treatment (Supplementary Figure 7). Expression of such cyto‐chemokines is largely driven through NF‐κB, among other pathways like COX‐1/2. To investigate the importance of NF‐κB and/or COX‐2 in the protective effects of PEPITEM, we analyzed their expression within the synovial microenvironment by Western blot. PEPITEM treatment significantly reduced the levels of both NF‐κB and COX‐2 protein within the joint when compared to control mice. (Figure 5H, I). Collectively, our data strongly indicate that PEPITEM can dampen the global inflammatory response within the synovial microenvironment impacting cellular migration to limit arthritis severity/pathology.

### PEPITEM therapy reduces disease severity and joint swelling

**Figure 6:**
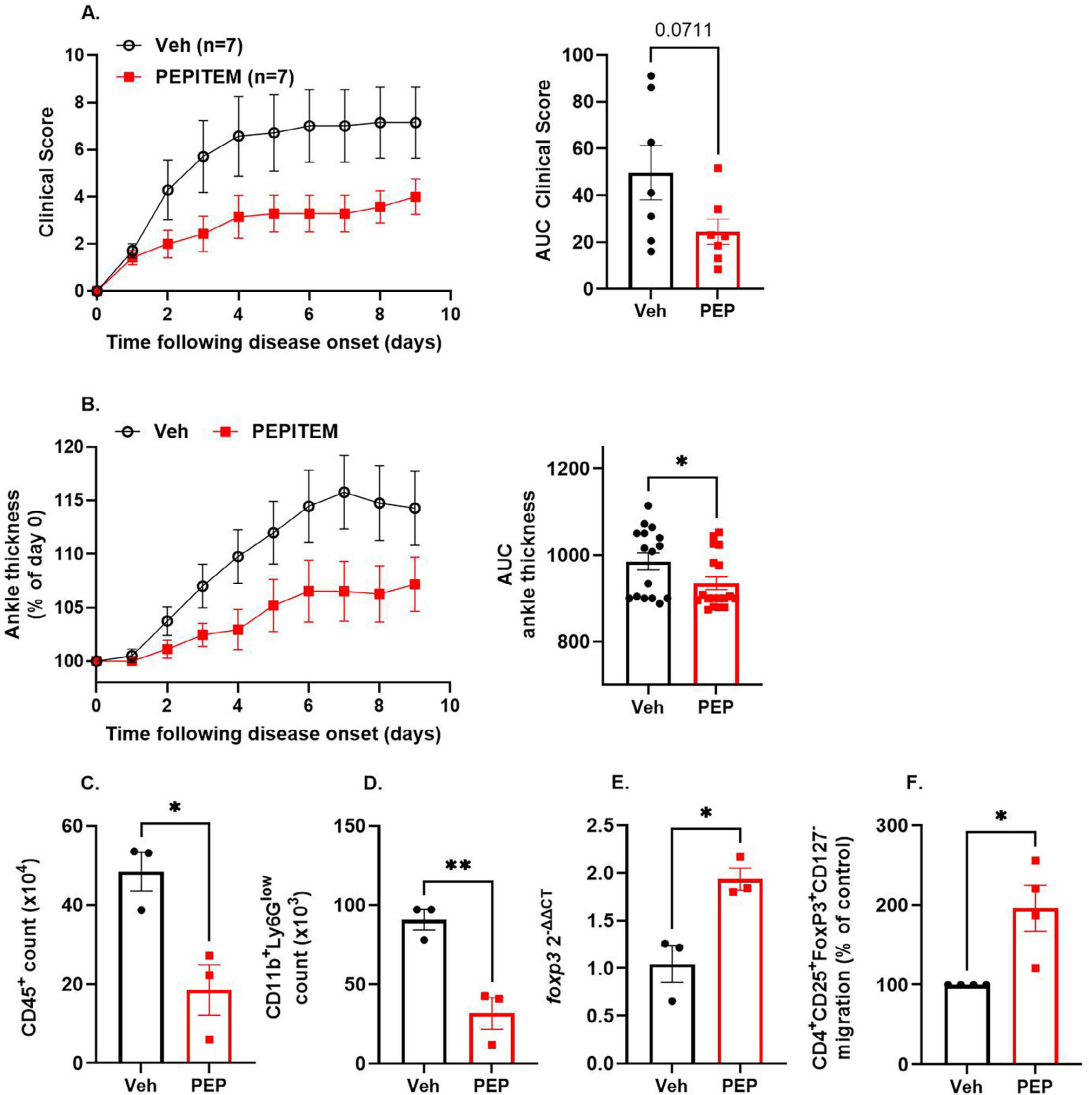
PEPITEM therapy reduces disease symptoms in mice with CIA. Mice with CIA were treated with Veh (black) or PEP (red) at first signs of inflammation and assessed for clinical signs of arthritis. (A) CIA clinical score over time and expressed as AUC, n = 8 mice per group. (B) CIA‐induced ankle thickness expressed as percentage change from baseline for each animal over time and AUC, n = 16 limbs from 8 mice per group. In A and B, ANOVA shows a significant effect of time (*P* < 0.05) and treatment (*P* < 0.001) on clinical score or joint thickness. Total number of synovial (C) CD45^+^ cells and (D) CD11b^+^Ly6G^−^ monocytes analyzed by flow cytometry, n = 3. (E) FACS‐sorted CD45^+^ cells were analyzed for foxp3 transcript by qPCR and plotted as 2^−ΔΔCT^ relative to Veh, n = 3. (F) Percentage of CD4^+^CD25^+^CD127^−^CD49d^−^ sorted Treg cells migration through inflamed endothelial cells normalized to Veh, n = 4. Data are mean ± SEM from at least 2 independent experiments. *P < 0.05 and **P < 0.01 by (C–E) unpaired or (F) paired *t*‐test compared to Veh. ANOVA, analysis of variance; AUC, area under the curve; CIA, collagen‐induced arthritis; CT, computed tomography; FACS, fluorescence‐activated cell sorting; PEP, PEPITEM‐PEG; qPCR, quantitative polymerase chain reaction; Veh, vehicle control.

Finally, we therapeutically administered PEPITEM at the onset of inflammation in mice with CIA to ascertain its utility as a therapeutic agent. PEPITEM therapy reduced the clinical severity of CIA over the treatment regimen, although this was borderline significant when comparing AUC to vehicle‐treated mice (Figure 6A). This reduction in clinical score was mirrored by a significant reduction in joint swelling/ankle thickness and total CD45^+^ leukocyte infiltration into the joint compared to control group (Figure 6B, C). We also observed a significant reduction of Ly6G^−/low^ monocytes (Figure 6D), with a tendency for reduced numbers of CD3^+^ T cells, B220^+^ B cells, and Ly6G^+^ neutrophils—albeit these were not statistically significant probably due to low n (data not shown). Interestingly, we observed a significant increase in the *foxp3* transcript in CD45^+^ cells retrieved from the inflamed joints of mice treated with PEPITEM compared to vehicle control (Figure 6E)—indicative of increased Treg cells within these joints.

To further address this, the impact of PEPITEM treatment on the migratory potential of purified human Treg cells (CD4^+^CD25^+^CD127^−^FOXP3^+^) was assessed using an in vitro migratory assay incorporating inflamed blood vascular endothelial cells.^6^ We observed significant increase in the percentage of Treg cells undergoing transendothelial migration when cells were treated with PEPITEM compared to vehicle control (Figure 6F); this is in contrast to PEPITEM suppressing the migration of CD4^+^ effector and central memory T cells.^6^ Of note, PEPITEM had no direct effect on the proliferation or polarization of CD4^+^ T cells into Treg cells (Supplementary Figure 8)—indicating specific effects on cell motility rather than effector function—thus, raising the possibility that PEPITEM could limit the migration of pathogenic leukocytes while simultaneously promoting the making of regulatory immune cells. These data collectively reveal that PEPITEM therapy has the potential to act in an immunosuppressive manner to limit disease severity and progression, thus offering a potential alternative therapeutic approach for patients.

## DISCUSSION

Current approved b/tsDMARDs target pathologic leukocytes (eg, rituximab), their cytokines (TNF inhibitor [TNFi]), or downstream signaling (JAK inhibitor^41^), but none restore endogenous regulatory pathways or reset tissue homeostasis to drive resolution. We reveal for the first time that a subset of treatment naive patients with early RA, as well as some patients with UA or PsA, have defects in the endogenous regulatory adiponectin–PEPITEM pathway and, as such, may benefit from replacement therapy aimed at the earliest phases of disease. Administration of synthetic PEPITEM in at risk mice (before onset of clinically apparent inflammation) limited disease onset, severity, and/or progression in multiple models of murine inflammatory arthritis—whereby PEPITEM was comparable to TNFi therapy without causing immunosuppression. Specifically, PEPITEM induced notable alterations in the leukocyte population and soluble mediator profile within the synovial microenvironment, promoting a shift toward a milieu characterized by anti‐inflammatory and proresolution properties, thereby attenuating the effector functions of M1‐like macrophages. Therapeutic treatment of mice with arthritis with PEPITEM at the first signs of inflammation (akin to patients with newly presenting early arthritis) was clinically beneficial. These proof‐of‐concept data reveal the possibility that a PEPITEM‐based agent could have clinical utility as an alternative and/or combinational therapy alongside current standard of care treatments for rheumatologic patients with defects in the endogenous adiponectin–PEPITEM pathway.

In humans, circulating plasma adiponectin levels positively correlate with adiponectin receptor expression on immune cells,^42^ among others (reviewed by Chimen et al^6^). Both smoking and obesity reduce plasma levels of adiponectin and its receptors^43^, ^44^ and are risk factors for onset of inflammatory arthritis. By contrast, women typically have higher adiponectin levels than men, linked to presence of estrogen‐regulating adipose tissue functionality,^45^ yet have increased risk of developing IMIDs. Few studies have examined circulating adiponectin levels in patients with early RA, whereby it has been reported that levels are elevated in patients with either less than 6‐month^46^ or less than 12‐month symptom^47^ duration when compared to age‐, sex‐, and body mass index (BMI)–matched HC—although adiponectin receptor levels were not analyzed in either of these studies. Indeed, we see a tendency for higher levels of adiponectin in some of the treatment naive patients with RA when compared to UA and CSA—although this was not statistically significant and may reflect increased BMI rather than a direct link to disease pathogenesis. However, no differences were reported in adiponectin concentrations between patients with UA and patients with RA with similar BMIs and symptom durations (six months),^48^ despite our data indicating differential adiponectin receptor expression/signaling between these groups. Age also plays a significant role in diminishing adiponectin receptor expression,^6^ along with reducing the activation and/or phosphorylation status of several downstream signaling molecules, including APPL‐1.^28^ These results in populations over 65 years old are akin to the results presented herein for patients with early RA or PsA with a median age of 58 years—although we see no age‐related correlation with adiponectin receptor expression (data not shown). Therefore, patients with early RA or PsA showing defects in adiponectin receptor expression and/or signaling are likely to exhibit reduced ability to produce PEPITEM, especially given the significant reductions seen in the expression of its parent protein *14‐3‐3ζ* by leukocytes.

Despite this, patients with RA and PsA exhibit normal or high levels of circulating PEPITEM, raising the question as to why these concentrations are unable to limit infiltration into the inflamed joint. Circulating proteins/peptides can be sequestered by plasma proteins, thereby limiting their bioavailability. Combination of predictive 3D models and co‐immunoprecipitation studies suggest that circulating PEPITEM can be sequestered by soluble ICAM‐1, which is known to be elevated in RA.^36^, ^37^ Moreover, local levels of PEPITEM are diminished in RA synovium, suggesting limited bioavailability to regulate lymphocyte migration, highlighting the potential utility of developing novel therapies aimed at restoring the local functionality of the adiponectin–PEPITEM pathway to treat patients at the earliest phases of arthritis, in which disease is more amenable to intervention.^49^ Indeed, we have previously shown that lymphocytes from patients with established RA were unable to respond to adiponectin but that this defect could be bypassed ex vivo with PEPITEM supplementation, restoring control over lymphocyte migration.^6^ First‐in‐man studies are now required to truly elucidate the therapeutic benefit of PEPITEM in such patients.

Patients with RA can be subdivided based on their synovial leukocyte pathotype, whereby aberrant trafficking and accumulation of different leukocyte subpopulations (lymphocyte, myeloid) into distinct anatomic regions within the synovium differentiates pathotypes.^50^ Moreover, a specific subset of sublining fibroblasts (FAP^+^THY1^+^) have been shown to drive leukocyte infiltration^34^ through cross‐talk with blood vascular endothelial cells.^51^ This bidirectional communication evolves as RA progresses and is distinct from that seen by fibroblasts in acutely resolving synovitis.^52^ A series of studies have established PEPITEM as an endogenous negative regulator of both lymphocyte^6^, ^7^, ^8^ and monocyte/macrophage migration^7^, ^8^ in murine models of aging and IMIDs,^6^, ^7^, ^8^ including Sjögren syndrome,^6^ SLE,^7^ and psoriasis.^9^ Indeed, our data further extend these observations into murine models of arthritis and indicate that the actions of PEPITEM are synovial leukocyte pathotype agnostic at least in preclinical models. Considering the different arthritis models employed in this study are driven by different underlying disease mechanisms (acute inflammation [MSU] vs autoimmune [CIA, AIA]), our findings further highlight the fundamental role PEPITEM plays in regulating immune‐mediated inflammation, irrespective of the mechanistic trigger. Beyond the immune system, PEPITEM has been shown to directly suppress fibroblast function in vitro, reducing the secretion of both IL‐6 and TNFα.^9^ This raises the possibility that PEPITEM could regulate leukocyte trafficking by direct actions on endothelium,^6^ as well as indirectly by altering fibroblast–endothelial cross‐talk. Moreover, its direct actions on fibroblasts have the potential to reset the synovial microenvironment through other fibroblast–cell communication pathways.

Our latest data indicate that PEPITEM differentially regulates the migration of pathogenic effector cells versus regulatory immune cells to promote resolution of inflammation. Of note, this appears to be restricted to influencing migration capacity, with PEPITEM exhibiting no impact on T cell proliferation or polarization responses. At least two putative receptors/binding partners have been identified for PEPITEM, cadherin‐15 on inflamed endothelial cells^6^ and NCAM‐1 (CD56) on osteoblasts.^29^ Although a subset of CD3^+^CD56^+^ Treg cells exist that could directly bind PEPITEM, it is unclear whether the promigratory activity of PEPITEM on Treg cells is due to its direct binding of Treg cells or through an indirect mechanism such as differential sphingosine 1‐phosphate (S1P) receptor engagement.^6^ Further studies are now urgently required to dissect the molecular mechanism by which PEPITEM can promote Treg cell migration—therapeutically mimicking this response could have significant clinical benefit across a multitude of conditions.

PEPITEM has been reported to promote leukocyte trafficking through secondary lymphoid tissues as a means to counteract the negative effects of obesity.^8^ Importantly, PEPITEM had no impact on lymph node function (leukocyte numbers or autoantibody levels) in the arthritis models, highlighting that leukocyte trafficking into and out of the draining lymph nodes remains functional while trafficking into peripheral tissues (inflamed joint) is perturbed. Given PEPITEM can induce S1P signaling, this represents a therapeutic advantage over current S1P modulators (eg, fingolimod), which cause lymphopenia^53^, ^54^ by trapping cells in the lymph node^55^, ^56^, ^57^ and indirectly limiting their availability to traffic normally to peripheral sites. By contrast, PEPITEM allows for normal transiting through lymphoid structures, only restricting leukocyte entry into sites of inflammation.^6^, ^7^, ^8^ Moreover, it promotes migration of Treg cells into sites of inflammation, further supporting tissue resolution. Although approved by the US Food and Drug Administration (FDA) for the treatment of relapsing–remitting multiple sclerosis, S1P modulators have a myriad of serious and systemic side effects, including cardio‐toxicity, increased susceptibility to opportunistic infections, and malignancy. In the context of rheumatic diseases, they have the potential to stabilize tertiary lymphoid structures, thereby conceivably exacerbating disease. Although further comprehensive immunologic studies are warranted to ascertain the precise impact of PEPITEM on lymphoid biology, our findings strongly suggest PEPITEM is unlikely to impede host responses against infection or diminish vaccine responses, thus offering a potential advantage over existing b/tsDMARDs and S1P modulators.

Numerous synovial monocyte and/or macrophage subpopulations have been identified in humans^58^, ^59^ and murine^60^, ^61^ models of inflammatory arthritis using scRNA‐Seq. Of particular importance has been the description of two MerTK^pos^ synovial macrophage subpopulations found in patients with RA in sustained remission that, when activated in vitro, secrete high levels of IL‐10 and resolvin‐D1,^58^ creating an anti‐inflammatory/proresolving milieu within the joint. Furthermore, a positive feedback loop driving M2‐like macrophage polarization in both RA and murine CIA through SIRT‐1 mediated inhibition of CCL2 (JE) and NF‐κB activity has been reported.^62^ Although similar decreases in JE and NF‐κB activity were seen in the synovium from PEPITEM‐treated mice, this was not associated with an elevation in M2‐like macrophage numbers. Likewise, PEPITEM administration reduced COX‐2, and to a lesser extent NF‐κB, activity in psoriatic murine skin,^9^ indicating broad immunoregulatory efficacy across tissues. As seen in a model of obesity,^8^ PEPITEM had the tendency to reduce synovial macrophage numbers—potentially by influencing monocyte differentiation to macrophages or by enhancing exit of synovial macrophages (as has been described in other tissue microenvironments^63^). At the level of the synovial monocyte, PEPITEM down‐regulated numerous functional and migration‐related pathways, which was reflected more broadly in the diminished pro‐inflammatory cyto/chemokine levels within the synovium microenvironment. Indeed, we found PEPITEM to negatively regulate synovial levels of IL‐16 and, to a lesser extent, TREM‐1, both of which are described to be induced during an exacerbated IL‐17–driven inflammatory response linked to tissue infiltration by inflammatory monocytes.^64^ Moreover, similar reductions in monocyte infiltration and pro‐inflammatory mediators, including TREM‐1, were observed in psoriatic skin from mice treated with PEPITEM when compared to untreated controls.^9^ Recent new insights have revealed that PEPITEM can act directly on macrophages in vitro, limiting their ability to release pro‐inflammatory mediators in response to stimuli,^9^ presumably through reductions in COX‐2 and NF‐κB seen in whole‐tissue level. Furthermore, the reduction in synovial monocyte numbers following PEPITEM are comparable to those seen with current standard of care (infliximab) treatment.^31^ Collectively, these studies further support a role for PEPITEM in dampening monocyte and macrophage function. Additional investigations are now needed to dissect the mechanism of action of PEPITEM on macrophage function and differentiation.

Our analysis herein has largely focused on monocyte/macrophage function, but that cannot rule out the possibility that PEPITEM might be regulating the functional responses of several different immune cell and stromal cell subpopulations. Given PEPITEM is known to regulate T cell and neutrophil trafficking in a multitude of preclinical models, it would be important to conduct further studies to assess the direct impact of PEPITEM on T cell activation and polarization, along with neutrophil effector functions to ascertain how these collectively contribute to amelioration of inflammatory arthritis. Key to this is understanding the direct effects of PEPITEM on the cells within the synovial microenvironment through in vitro and time‐course analysis, versus changes that are a consequence of reduced inflammation. Furthermore, a deeper understanding of the mechanism by which PEPITEM promotes Treg cell infiltration and whether it is also able to influence the behavior of other regulatory immune cells is urgently required. A limitation of our mechanistic study is the measurement of total NF‐κB (p65) protein levels rather than phosphorylated p65 (pp65) as a more direct marker of NF‐κB activation status, reflecting translocation and transcriptional activity.

Even when inflammation is therapeutically well controlled, current DMARDs do not reverse joint damage. In addition to its actions on immune cells and fibroblasts, PEPITEM also exerts novel anabolic osteogenic properties, enhancing bone formation and strength, while simultaneously limiting bone resorption.^29^ The dual immunomodulatory, osteogenic action of PEPITEM has the potential to re‐establish the intricate balance required for tissue homeostasis, and it may have the potential to reduce the need for polypharmacy. Specifically, the possibility of influencing disease trajectory in the subclinical/preclinical phases of RA is highly attractive, especially as there are no therapies approved for preclinical RA and, unlike PEPTIEM, current b/tsDMARDs are immunosuppressive in an otherwise healthy population. Importantly, the risk of toxicity from a natural peptide like PEPITEM is extremely low, as it typically circulates in the plasma to achieve systemic distribution. Moreover, our latest data indicate that endogenous PEPITEM appears to be sequestered by pathogenically high levels of circulating proteins, including soluble ICAM‐1, preventing it from being bioavailable to mediate its immunomodulatory effects. As such, its therapeutic role would be akin to that of replacement therapies, which focus on restoring the function of impaired homeostatic mechanisms. This type of approach contrasts what is employed by the majority of standard of care therapies currently available, which aim to limit factors associated with disease symptoms, such as neutralizing proinflammatory cytokines (eg, TNFα and IL‐6). Additionally, PEPITEM displays similar efficacy as standard of care steroids (clobetasol) in treating murine psoriasis without the long‐term systemic adverse effects.^65^, ^66^ This opens the possibility of combining PEPITEM with approved drugs to boost treatment efficacy, provided synergistic effects are present. Such an approach could also reduce reliance on steroids in the earliest phases of RA/PsA, thus minimizing the associated severe side effects or enabling lower, longer‐term dosing without sacrificing efficacy. Overall, conducting more detailed studies across different disease stages and patient populations will help elucidate the therapeutic potential and optimal use of PEPITEM therapy in managing inflammatory arthritis and related conditions.

In conclusion, dysregulation of the endogenous regulatory adiponectin–PEPITEM pathway manifests in certain patients during inception RA and PsA, potentially preceding formal clinical classification. Subclinical and therapeutic modulation of the pathway using PEPITEM yields observable reversal of clinical disease manifestations in mice, concurrently influencing the immunologic milieu and inflammatory profile within the synovial microenvironment. Crucially the multimodal activity of PEPITEM—suppressing immune cell and fibroblast function while simultaneously promoting osteoblastogenesis—enables it to regulate all aspects of cellular pathology within arthritis to alleviate symptoms and restore homeostasis. Consequently, pharmaceutical agents based on PEPITEM hold promise for clinical application among rheumatologic patients exhibiting deficiencies in this endogenous regulatory pathway, offering prospects for repairing arthritis‐induced bone damage.

## AUTHOR CONTRIBUTIONS

All authors contributed to at least one of the following manuscript preparation roles: conceptualization AND/OR methodology, software, investigation, formal analysis, data curation, visualization, and validation AND drafting or reviewing/editing the final draft. As corresponding author, Pro McGettrick confirms that all authors have provided the final approval of the version to be published and takes responsibility for the affirmations regarding article submission (eg, not under consideration by another journal), the integrity of the data presented, and the statements regarding compliance with institutional review board/Declaration of Helsinki requirements.

## Supporting information


**Disclosure form**.

**Supplementary Figure 1: Gating strategy for identifying immune cell populations in murine models of arthritis**. Leukocytes were identified using forward (FSC) and side scatter (SSC), prior to defining single and then live CD45^+^ leukocytes. Gating strategy to identify CD3^+^ T‐cells, Ly6G^+^CD11b^+^Ly6C^+^ neutrophils, CD11b^+^Ly6C^+^ monocytes and CD11b^+^F4/80^+^ macrophages.
**Supplementary Figure 2: Gating strategy for adiponectin receptor expression on human peripheral blood mononuclear cells**. The flow cytometry gating strategy used to phenotype adiponectin receptor expression on human peripheral mononuclear cells. Gating strategy to defined PBMC based on FSC and SSC, prior to determine single cells and then live PBMC based on exclusion of a live/dead marker prior to identifying CD19^+^ positive B‐cells and CD14^+^ monocytes. The percentage of PBMC, B‐cells or monocytes positively staining for adiponectin receptor was determined by gating to exclude any cells falling within the isotype region. Overlay histograms show expression of adiponectin receptor 1 and 2 (ADIPOR1, ADIPOR2, respectively) for each patient group.
**Supplementary Figure 3: Expression of adiponectin receptor 1 on leukocytes from patients with RA and PsA**. Peripheral blood mononuclear cells (PBMC) were isolated from patients with clinically suspect arthralgia (CSA, n=14), unclassified arthritis (UA, n=26), early RA (RA, n=47) or PsA (n=14) or age‐matched healthy controls (HC, n=12). Adiponectin receptor 1 **(A)** gene or **(B‐D)** protein expression was assessed on (A‐B) PBMC, (C) CD19^+^ B‐cells or (D) monocytes by (A) qPCR or (B‐D) flow cytometry. **(A)** Gene expression was expressed as 2^−ΔCT^ relative to the housekeeping gene ‐ β_2_M. ANOVA shows a significant effect of patient group on adiponectin receptor 1 expression, p<0.05. Protein expression was expressed as the percentage of **(B)** PBMC, or **(C)** B‐cells or **(D)** monocytes positive for adiponectin receptor staining. **(E)** Plasma adiponectin concentrations expressed as μg/ml – n=10 per group, with n=17 for HC. Data are mean ± SEM. *= p<0.05 by Dunnett.
**Supplementary Figure 4: PEPITEM bioavailability is influenced by interactions with sICAM‐1. (A‐D)** Full length ICAM‐1 or **(E‐H)** soluble ICAM‐1 were ran with PEPITEM in (A, E) AlphaFold‐Multimer followed by analysed of rank 1 model (B‐D, F‐H) using ChimeraX. Predicted aligned error heat map for one model of **(B)** ICAM‐1 or **(F)** sICAM‐1 domain and PEPITEM, where blue and yellow indicate low or high error, respectively. ChimeraX modelling of rank 1 model for PEPITEM interactions with **(C‐D)** ICAM‐1 or **(G‐H)** sICAM‐1 coloured by pLDDT. **(C‐D, G‐H)** Magnified view of binding location of PEPITEM on ICAM‐1 revealing **(C)** 7 and **(G)** 6 predicted hydrogen bonds ‐ high likelihood (blue), low (orange) ‐ and **(D)** 32 and **(H)** 37 pseudobonds based on a distance of 5Å, confidence indicated by pLDDT colour as above.
**Supplementary Figure 5: PEPITEM inhibitory activity is comparable to that of infliximab, but has no effect on lymph node responses. (A, C, E)** In CIA, mice were treated with vehicle control (Veh, black) or PEPITEM‐PEG (PEP, red) from day 21 onwards. **(A)** Autoantibody production, **(C)** CD19^+^ cells in the synovium and **(E)** CD45^+^ cells in the draining lymph node from CIA mice. **(B, D)** In the AIA model, mice were treated with vehicle control (Veh, black) or PEPITEM‐PEG (PEP, red) or **(B)** infliximab (IFX, blue) from day 21 onwards. AIA‐induced knee thickness (n=6) was expressed as AUC for the percentage change from baseline for each animal. In B, ANOVA shows a significant effect of treatment on joint thickness, p<0.05. **(D)** CD45^+^ cells in the draining lymph node from AIA mice. Data are mean ± SEM from at least n=2 independent experiment. *= p<0.05 by (B) Bonferroni post‐test or (D) unpaired t‐test compared to vehicle control.
**Supplementary Figure 6: scRNAseq quality control and data extraction (A)** UMAP of cells labelled by sample. **(B)** Heatmap of top genes per cluster. **(C‐D)** Differential abundance analysis of cell types in vehicle or PEPITEM treated cells.
**Supplementary Figure 7: PEPITEM had no effect on TNFα or prostanoid levels**. Synovial tissue from AIA mice were treated with vehicle control (Veh, black) or PEPITEM (PEP, red) was collected at the peak of inflammation (day 3). After tissue digestion, the acellular fraction was analysed by ELISA for **(A)** TREM‐1, **(B)** TNFα, **(C)** PGD_2_, **(D)** PGE_2_, **(E)** 5‐HETE and **(F)** 12‐HETE expressed as (B, F) ng/ml or (A, C‐E) pg/ml n=7‐8 mice per condition. Data are mean ± SEM from at least n=1 independent experiment, analysed using unpaired t‐test.
**Supplementary Figure 8: PEPITEM had no effect on T‐cell activation or polarisation**. CD4+ native T‐cells were polarised towards Treg or T_H1_ using a combination of antibody cocktails and differentiation media, treated in the presence of absence of PEPITEM. Gating strategy to defined T‐cells based on FSC and SSC, prior to determine single cells and then live T‐cells based on exclusion of a live/dead marker. Gating strategy used CD3^+^CD4^+^ to identify T‐cells, **(B)** CD25+Foxp3+ to denote Tregs and **(A)** Ki67 was used to assess T‐cell proliferation. Data are mean ± SEM from at least n=3 independent experiment.
**Supplementary Figure 9: Western blot gels for COX2, NF‐κB and β‐actin**. COX‐2, NF‐κB p65 and β‐actin protein in whole knee joint homogenates from vehicle or PEPITEM treated AIA mice was assessed at the peak of inflammation by western blot analysis. Images represent three separate independent experiments run each with n=4‐8 mice per group pooled.
**Supplementary Figure 10: Western blot gels for APPL‐1, and β‐actin**. APPL‐1 and β‐actin protein in peripheral blood mononuclear cells (PBMC) from patients with clinically suspect arthralgia (CSA), unclassified arthritis (UA), early RA (RA) or age‐matched healthy controls (HC) was assessed by western blot analysis. Images represent two separate independent experiments run each with n=2 patients per gel.

**Supplementary Table 1** Patient Clinical Characteristics
**Supplementary Table 2:** DEG in monocytes from PEPITEM vs vehicle treated mice
**Supplementary Table 3:** DEG in macrophages from PEPITEM vs vehicle treated mice
