# Supplementary material for: PEPITEM Regulates the Synovial Microenvironment During Immune‐Mediated Inflammatory Arthritis to Limit Disease
Source: Arthritis Rheumatol. 2026 Apr 13;78(7):1446–64. doi: 10.1002/art.70108 (PMC13313099; doi:10.1002/art.70108)
Supplement: Supplementary file 1 — Disclosure form. [file ART-78-1446-s002.pdf]

ICMJE DISCLOSURE FORM

Date:_03/06/2025
Your Name: Mussarat Wahid
Manuscript Title: PEPITEM regulates the synovial microenvironment during immune-mediated inflammatory arthritis to limit disease
Manuscript number (if known): ar-25-0320

In the interest of transparency, we ask you to disclose all relationships/activities/interests listed below that are related to the content of your manuscript. "Related" means any relation with for-profit or not-for-profit third parties whose interests may be affected by the content of the manuscript. Disclosure represents a commitment to transparency and does not necessarily indicate a bias. If you are in doubt about whether to list a relationship/activity/interest, it is preferable that you do so.

The following questions apply to the author's relationships/activities/interests as they relate to the current manuscript only.

The author's relationships/activities/interests should be defined broadly. For example, if your manuscript pertains to the epidemiology of hypertension, you should declare all relationships with manufacturers of antihypertensive medication, even if that medication is not mentioned in the manuscript.

In item #1 below, report all support for the work reported in this manuscript without time limit. For all other items, the time frame for disclosure is the past 36 months.

		**Name all entities with whom you have this relationship or indicate none (add rows as needed)**	**Specifications/Comments (e.g., if payments were made to you or to your institution)**
		**Time frame: Since the initial planning of the work**	
1	All support for the present manuscript (e.g., funding, provision of study materials, medical writing, article processing charges, etc.) **No time limit for this item.**	X	
		**Time frame: past 36 months**	
2	Grants or contracts from any entity (if not indicated in item #1 above).	X	
3	Royalties or licenses	X	
4	Consulting fees	X	

5	Payment or honoraria for lectures, presentations, speakers bureaus, manuscript writing or educational events	X	
6	Payment for expert testimony	X	
7	Support for attending meetings and/or travel	X	
8	Patents planned, issued or pending	X	
9	Participation on a Data Safety Monitoring Board or Advisory Board	X	
10	Leadership or fiduciary role in other board, society, committee or advocacy group, paid or unpaid	X	
11	Stock or stock options	X	
12	Receipt of equipment, materials, drugs, medical writing, gifts or other services	X	
13	Other financial or non-financial interests	X	

Please place an "X" next to the following statement to indicate your agreement:

X I certify that I have answered every question and have not altered the wording of any of the questions on this form.

ICMJE DISCLOSURE FORM

Date:_03/06/2025
Your Name: Samuel Kemble
Manuscript Title: PEPITEM regulates the synovial microenvironment during immune-mediated inflammatory arthritis to limit disease
Manuscript number (if known): ar-25-0320

In the interest of transparency, we ask you to disclose all relationships/activities/interests listed below that are related to the content of your manuscript. "Related" means any relation with for-profit or not-for-profit third parties whose interests may be affected by the content of the manuscript. Disclosure represents a commitment to transparency and does not necessarily indicate a bias. If you are in doubt about whether to list a relationship/activity/interest, it is preferable that you do so.

The following questions apply to the author's relationships/activities/interests as they relate to the current manuscript only.

The author's relationships/activities/interests should be defined broadly. For example, if your manuscript pertains to the epidemiology of hypertension, you should declare all relationships with manufacturers of antihypertensive medication, even if that medication is not mentioned in the manuscript.

In item #1 below, report all support for the work reported in this manuscript without time limit. For all other items, the time frame for disclosure is the past 36 months.

		**Name all entities with whom you have this relationship or indicate none (add rows as needed)**	**Specifications/Comments (e.g., if payments were made to you or to your institution)**
		**Time frame: Since the initial planning of the work**	
1	All support for the present manuscript (e.g., funding, provision of study materials, medical writing, article processing charges, etc.) **No time limit for this item.**	X	
		**Time frame: past 36 months**	
2	Grants or contracts from any entity (if not indicated in item #1 above).	X	
3	Royalties or licenses	X	
4	Consulting fees	X	

5	Payment or honoraria for lectures, presentations, speakers bureaus, manuscript writing or educational events	X	
6	Payment for expert testimony	X	
7	Support for attending meetings and/or travel	X	
8	Patents planned, issued or pending	X	
9	Participation on a Data Safety Monitoring Board or Advisory Board	X	
10	Leadership or fiduciary role in other board, society, committee or advocacy group, paid or unpaid	X	
11	Stock or stock options	X	
12	Receipt of equipment, materials, drugs, medical writing, gifts or other services	X	
13	Other financial or non-financial interests	X	

Please place an "X" next to the following statement to indicate your agreement:

X I certify that I have answered every question and have not altered the wording of any of the questions on this form.

ICMJE DISCLOSURE FORM

Date:_03/06/2025
Your Name: Oladimeji Abudu
Manuscript Title: PEPITEM regulates the synovial microenvironment during immune-mediated inflammatory arthritis to limit disease
Manuscript number (if known): ar-25-0320

In the interest of transparency, we ask you to disclose all relationships/activities/interests listed below that are related to the content of your manuscript. "Related" means any relation with for-profit or not-for-profit third parties whose interests may be affected by the content of the manuscript. Disclosure represents a commitment to transparency and does not necessarily indicate a bias. If you are in doubt about whether to list a relationship/activity/interest, it is preferable that you do so.

The following questions apply to the author's relationships/activities/interests as they relate to the current manuscript only.

The author's relationships/activities/interests should be defined broadly. For example, if your manuscript pertains to the epidemiology of hypertension, you should declare all relationships with manufacturers of antihypertensive medication, even if that medication is not mentioned in the manuscript.

In item #1 below, report all support for the work reported in this manuscript without time limit. For all other items, the time frame for disclosure is the past 36 months.

		**Name all entities with whom you have this relationship or indicate none (add rows as needed)**	**Specifications/Comments (e.g., if payments were made to you or to your institution)**
**Time frame: Since the initial planning of the work**			
1	All support for the present manuscript (e.g., funding, provision of study materials, medical writing, article processing charges, etc.) **No time limit for this item.**	X	
**Time frame: past 36 months**			
2	Grants or contracts from any entity (if not indicated in item #1 above).	X	
3	Royalties or licenses	X	
4	Consulting fees	X	

5	Payment or honoraria for lectures, presentations, speakers bureaus, manuscript writing or educational events	X	
6	Payment for expert testimony	X	
7	Support for attending meetings and/or travel	X	
8	Patents planned, issued or pending	X	
9	Participation on a Data Safety Monitoring Board or Advisory Board	X	
10	Leadership or fiduciary role in other board, society, committee or advocacy group, paid or unpaid	X	
11	Stock or stock options	X	
12	Receipt of equipment, materials, drugs, medical writing, gifts or other services	X	
13	Other financial or non-financial interests	X	

Please place an "X" next to the following statement to indicate your agreement:

X I certify that I have answered every question and have not altered the wording of any of the questions on this form.

ICMJE DISCLOSURE FORM

Date:_03/06/2025
Your Name: Anella Saviano
Manuscript Title: PEPITEM regulates the synovial microenvironment during immune-mediated inflammatory arthritis to limit disease
Manuscript number (if known): ar-25-0320

In the interest of transparency, we ask you to disclose all relationships/activities/interests listed below that are related to the content of your manuscript. "Related" means any relation with for-profit or not-for-profit third parties whose interests may be affected by the content of the manuscript. Disclosure represents a commitment to transparency and does not necessarily indicate a bias. If you are in doubt about whether to list a relationship/activity/interest, it is preferable that you do so.

The following questions apply to the author's relationships/activities/interests as they relate to the current manuscript only.

The author's relationships/activities/interests should be defined broadly. For example, if your manuscript pertains to the epidemiology of hypertension, you should declare all relationships with manufacturers of antihypertensive medication, even if that medication is not mentioned in the manuscript.

In item #1 below, report all support for the work reported in this manuscript without time limit. For all other items, the time frame for disclosure is the past 36 months.

		**Name all entities with whom you have this relationship or indicate none (add rows as needed)**	**Specifications/Comments (e.g., if payments were made to you or to your institution)**
**Time frame: Since the initial planning of the work**			
1	All support for the present manuscript (e.g., funding, provision of study materials, medical writing, article processing charges, etc.) **No time limit for this item.**	X	
**Time frame: past 36 months**			
2	Grants or contracts from any entity (if not indicated in item #1 above).	X	
3	Royalties or licenses	X	
4	Consulting fees	X	

5	Payment or honoraria for lectures, presentations, speakers bureaus, manuscript writing or educational events	X	
6	Payment for expert testimony	X	
7	Support for attending meetings and/or travel	X	
8	Patents planned, issued or pending	X	
9	Participation on a Data Safety Monitoring Board or Advisory Board	X	
10	Leadership or fiduciary role in other board, society, committee or advocacy group, paid or unpaid	X	
11	Stock or stock options	X	
12	Receipt of equipment, materials, drugs, medical writing, gifts or other services	X	
13	Other financial or non-financial interests	X	

Please place an "X" next to the following statement to indicate your agreement:

X I certify that I have answered every question and have not altered the wording of any of the questions on this form.

ICMJE DISCLOSURE FORM

Date:_03/06/2025
Your Name: Christopher Mahony
Manuscript Title: PEPITEM regulates the synovial microenvironment during immune-mediated inflammatory arthritis to limit disease
Manuscript number (if known): ar-25-0320

In the interest of transparency, we ask you to disclose all relationships/activities/interests listed below that are related to the content of your manuscript. "Related" means any relation with for-profit or not-for-profit third parties whose interests may be affected by the content of the manuscript. Disclosure represents a commitment to transparency and does not necessarily indicate a bias. If you are in doubt about whether to list a relationship/activity/interest, it is preferable that you do so.

The following questions apply to the author's relationships/activities/interests as they relate to the current manuscript only.

The author's relationships/activities/interests should be defined broadly. For example, if your manuscript pertains to the epidemiology of hypertension, you should declare all relationships with manufacturers of antihypertensive medication, even if that medication is not mentioned in the manuscript.

In item #1 below, report all support for the work reported in this manuscript without time limit. For all other items, the time frame for disclosure is the past 36 months.

		**Name all entities with whom you have this relationship or indicate none (add rows as needed)**	**Specifications/Comments (e.g., if payments were made to you or to your institution)**
		**Time frame: Since the initial planning of the work**	
1	All support for the present manuscript (e.g., funding, provision of study materials, medical writing, article processing charges, etc.) **No time limit for this item.**	X	
		**Time frame: past 36 months**	
2	Grants or contracts from any entity (if not indicated in item #1 above).	X	
3	Royalties or licenses	X	
4	Consulting fees	X	

5	Payment or honoraria for lectures, presentations, speakers bureaus, manuscript writing or educational events	X	
6	Payment for expert testimony	X	
7	Support for attending meetings and/or travel	X	
8	Patents planned, issued or pending	X	
9	Participation on a Data Safety Monitoring Board or Advisory Board	X	
10	Leadership or fiduciary role in other board, society, committee or advocacy group, paid or unpaid	X	
11	Stock or stock options	X	
12	Receipt of equipment, materials, drugs, medical writing, gifts or other services	X	
13	Other financial or non-financial interests	X	

Please place an "X" next to the following statement to indicate your agreement:

X I certify that I have answered every question and have not altered the wording of any of the questions on this form.

ICMJE DISCLOSURE FORM

Date:_03/06/2025
Your Name: Jonathan W Lewis
Manuscript Title: PEPITEM regulates the synovial microenvironment during immune-mediated inflammatory arthritis to limit disease
Manuscript number (if known): ar-25-0320

In the interest of transparency, we ask you to disclose all relationships/activities/interests listed below that are related to the content of your manuscript. "Related" means any relation with for-profit or not-for-profit third parties whose interests may be affected by the content of the manuscript. Disclosure represents a commitment to transparency and does not necessarily indicate a bias. If you are in doubt about whether to list a relationship/activity/interest, it is preferable that you do so.

The following questions apply to the author's relationships/activities/interests as they relate to the current manuscript only.

The author's relationships/activities/interests should be defined broadly. For example, if your manuscript pertains to the epidemiology of hypertension, you should declare all relationships with manufacturers of antihypertensive medication, even if that medication is not mentioned in the manuscript.

In item #1 below, report all support for the work reported in this manuscript without time limit. For all other items, the time frame for disclosure is the past 36 months.

		**Name all entities with whom you have this relationship or indicate none (add rows as needed)**	**Specifications/Comments (e.g., if payments were made to you or to your institution)**
		**Time frame: Since the initial planning of the work**	
1	All support for the present manuscript (e.g., funding, provision of study materials, medical writing, article processing charges, etc.) **No time limit for this item.**	X	
		**Time frame: past 36 months**	
2	Grants or contracts from any entity (if not indicated in item #1 above).	X	
3	Royalties or licenses	X	
4	Consulting fees	X	

5	Payment or honoraria for lectures, presentations, speakers bureaus, manuscript writing or educational events	X	
6	Payment for expert testimony	X	
7	Support for attending meetings and/or travel	X	
8	Patents planned, issued or pending	X	
9	Participation on a Data Safety Monitoring Board or Advisory Board	X	
10	Leadership or fiduciary role in other board, society, committee or advocacy group, paid or unpaid	X	
11	Stock or stock options	X	
12	Receipt of equipment, materials, drugs, medical writing, gifts or other services	X	
13	Other financial or non-financial interests	X	

Please place an "X" next to the following statement to indicate your agreement:

X I certify that I have answered every question and have not altered the wording of any of the questions on this form.

ICMJE DISCLOSURE FORM

Date:_03/06/2025
Your Name: Thomas Nicholson
Manuscript Title: PEPITEM regulates the synovial microenvironment during immune-mediated inflammatory arthritis to limit disease
Manuscript number (if known): ar-25-0320

In the interest of transparency, we ask you to disclose all relationships/activities/interests listed below that are related to the content of your manuscript. "Related" means any relation with for-profit or not-for-profit third parties whose interests may be affected by the content of the manuscript. Disclosure represents a commitment to transparency and does not necessarily indicate a bias. If you are in doubt about whether to list a relationship/activity/interest, it is preferable that you do so.

The following questions apply to the author's relationships/activities/interests as they relate to the current manuscript only.

The author's relationships/activities/interests should be defined broadly. For example, if your manuscript pertains to the epidemiology of hypertension, you should declare all relationships with manufacturers of antihypertensive medication, even if that medication is not mentioned in the manuscript.

In item #1 below, report all support for the work reported in this manuscript without time limit. For all other items, the time frame for disclosure is the past 36 months.

		**Name all entities with whom you have this relationship or indicate none (add rows as needed)**	**Specifications/Comments (e.g., if payments were made to you or to your institution)**
**Time frame: Since the initial planning of the work**			
1	All support for the present manuscript (e.g., funding, provision of study materials, medical writing, article processing charges, etc.) **No time limit for this item.**	X	
**Time frame: past 36 months**			
2	Grants or contracts from any entity (if not indicated in item #1 above).	X	
3	Royalties or licenses	X	
4	Consulting fees	X	

5	Payment or honoraria for lectures, presentations, speakers bureaus, manuscript writing or educational events	X	
6	Payment for expert testimony	X	
7	Support for attending meetings and/or travel	X	
8	Patents planned, issued or pending	X	
9	Participation on a Data Safety Monitoring Board or Advisory Board	X	
10	Leadership or fiduciary role in other board, society, committee or advocacy group, paid or unpaid	X	
11	Stock or stock options	X	
12	Receipt of equipment, materials, drugs, medical writing, gifts or other services	X	
13	Other financial or non-financial interests	X	

Please place an "X" next to the following statement to indicate your agreement:

X I certify that I have answered every question and have not altered the wording of any of the questions on this form.

ICMJE DISCLOSURE FORM

Date:_03/06/2025
Your Name: Anna Schettino
Manuscript Title: PEPITEM regulates the synovial microenvironment during immune-mediated inflammatory arthritis to limit disease
Manuscript number (if known): ar-25-0320

In the interest of transparency, we ask you to disclose all relationships/activities/interests listed below that are related to the content of your manuscript. "Related" means any relation with for-profit or not-for-profit third parties whose interests may be affected by the content of the manuscript. Disclosure represents a commitment to transparency and does not necessarily indicate a bias. If you are in doubt about whether to list a relationship/activity/interest, it is preferable that you do so.

The following questions apply to the author's relationships/activities/interests as they relate to the current manuscript only.

The author's relationships/activities/interests should be defined broadly. For example, if your manuscript pertains to the epidemiology of hypertension, you should declare all relationships with manufacturers of antihypertensive medication, even if that medication is not mentioned in the manuscript.

In item #1 below, report all support for the work reported in this manuscript without time limit. For all other items, the time frame for disclosure is the past 36 months.

		**Name all entities with whom you have this relationship or indicate none (add rows as needed)**	**Specifications/Comments (e.g., if payments were made to you or to your institution)**
**Time frame: Since the initial planning of the work**			
1	All support for the present manuscript (e.g., funding, provision of study materials, medical writing, article processing charges, etc.) **No time limit for this item.**	X	
**Time frame: past 36 months**			
2	Grants or contracts from any entity (if not indicated in item #1 above).	X	
3	Royalties or licenses	X	
4	Consulting fees	X	

5	Payment or honoraria for lectures, presentations, speakers bureaus, manuscript writing or educational events	X	
6	Payment for expert testimony	X	
7	Support for attending meetings and/or travel	X	
8	Patents planned, issued or pending	X	
9	Participation on a Data Safety Monitoring Board or Advisory Board	X	
10	Leadership or fiduciary role in other board, society, committee or advocacy group, paid or unpaid	X	
11	Stock or stock options	X	
12	Receipt of equipment, materials, drugs, medical writing, gifts or other services	X	
13	Other financial or non-financial interests	X	

Please place an "X" next to the following statement to indicate your agreement:

X I certify that I have answered every question and have not altered the wording of any of the questions on this form.

ICMJE DISCLOSURE FORM

Date:_03/06/2025
Your Name: Kathryn Frost
Manuscript Title: PEPITEM regulates the synovial microenvironment during immune-mediated inflammatory arthritis to limit disease
Manuscript number (if known): ar-25-0320

In the interest of transparency, we ask you to disclose all relationships/activities/interests listed below that are related to the content of your manuscript. "Related" means any relation with for-profit or not-for-profit third parties whose interests may be affected by the content of the manuscript. Disclosure represents a commitment to transparency and does not necessarily indicate a bias. If you are in doubt about whether to list a relationship/activity/interest, it is preferable that you do so.

The following questions apply to the author's relationships/activities/interests as they relate to the current manuscript only.

The author's relationships/activities/interests should be defined broadly. For example, if your manuscript pertains to the epidemiology of hypertension, you should declare all relationships with manufacturers of antihypertensive medication, even if that medication is not mentioned in the manuscript.

In item #1 below, report all support for the work reported in this manuscript without time limit. For all other items, the time frame for disclosure is the past 36 months.

		**Name all entities with whom you have this relationship or indicate none (add rows as needed)**	**Specifications/Comments (e.g., if payments were made to you or to your institution)**
		**Time frame: Since the initial planning of the work**	
1	All support for the present manuscript (e.g., funding, provision of study materials, medical writing, article processing charges, etc.) **No time limit for this item.**	X	
		**Time frame: past 36 months**	
2	Grants or contracts from any entity (if not indicated in item #1 above).	X	
3	Royalties or licenses	X	
4	Consulting fees	X	

5	Payment or honoraria for lectures, presentations, speakers bureaus, manuscript writing or educational events	X	
6	Payment for expert testimony	X	
7	Support for attending meetings and/or travel	X	
8	Patents planned, issued or pending	X	
9	Participation on a Data Safety Monitoring Board or Advisory Board	X	
10	Leadership or fiduciary role in other board, society, committee or advocacy group, paid or unpaid	X	
11	Stock or stock options	X	
12	Receipt of equipment, materials, drugs, medical writing, gifts or other services	X	
13	Other financial or non-financial interests	X	

Please place an "X" next to the following statement to indicate your agreement:

X I certify that I have answered every question and have not altered the wording of any of the questions on this form.

ICMJE DISCLOSURE FORM

Date:_14/11/2025
Your Name: Jenefa Begum
Manuscript Title: PEPITEM regulates the synovial microenvironment during immune-mediated inflammatory arthritis to limit disease
Manuscript number (if known): ar-25-0320

In the interest of transparency, we ask you to disclose all relationships/activities/interests listed below that are related to the content of your manuscript. "Related" means any relation with for-profit or not-for-profit third parties whose interests may be affected by the content of the manuscript. Disclosure represents a commitment to transparency and does not necessarily indicate a bias. If you are in doubt about whether to list a relationship/activity/interest, it is preferable that you do so.

The following questions apply to the author's relationships/activities/interests as they relate to the current manuscript only.

The author's relationships/activities/interests should be defined broadly. For example, if your manuscript pertains to the epidemiology of hypertension, you should declare all relationships with manufacturers of antihypertensive medication, even if that medication is not mentioned in the manuscript.

In item #1 below, report all support for the work reported in this manuscript without time limit. For all other items, the time frame for disclosure is the past 36 months.

		**Name all entities with whom you have this relationship or indicate none (add rows as needed)**	**Specifications/Comments (e.g., if payments were made to you or to your institution)**
		**Time frame: Since the initial planning of the work**	
1	All support for the present manuscript (e.g., funding, provision of study materials, medical writing, article processing charges, etc.) **No time limit for this item.**	X	
		**Time frame: past 36 months**	
2	Grants or contracts from any entity (if not indicated in item #1 above).	X	
3	Royalties or licenses	X	
4	Consulting fees	X	

5	Payment or honoraria for lectures, presentations, speakers bureaus, manuscript writing or educational events	X	
6	Payment for expert testimony	X	
7	Support for attending meetings and/or travel	X	
8	Patents planned, issued or pending	X	
9	Participation on a Data Safety Monitoring Board or Advisory Board	X	
10	Leadership or fiduciary role in other board, society, committee or advocacy group, paid or unpaid	X	
11	Stock or stock options	X	
12	Receipt of equipment, materials, drugs, medical writing, gifts or other services	X	
13	Other financial or non-financial interests	X	

Please place an "X" next to the following statement to indicate your agreement:

X I certify that I have answered every question and have not altered the wording of any of the questions on this form.

ICMJE DISCLOSURE FORM

Date:_14/11/2025
Your Name: Alyssa M Urbanowski
Manuscript Title: PEPITEM regulates the synovial microenvironment during immune-mediated inflammatory arthritis to limit disease
Manuscript number (if known): ar-25-0320

In the interest of transparency, we ask you to disclose all relationships/activities/interests listed below that are related to the content of your manuscript. "Related" means any relation with for-profit or not-for-profit third parties whose interests may be affected by the content of the manuscript. Disclosure represents a commitment to transparency and does not necessarily indicate a bias. If you are in doubt about whether to list a relationship/activity/interest, it is preferable that you do so.

The following questions apply to the author's relationships/activities/interests as they relate to the current manuscript only.

The author's relationships/activities/interests should be defined broadly. For example, if your manuscript pertains to the epidemiology of hypertension, you should declare all relationships with manufacturers of antihypertensive medication, even if that medication is not mentioned in the manuscript.

In item #1 below, report all support for the work reported in this manuscript without time limit. For all other items, the time frame for disclosure is the past 36 months.

		**Name all entities with whom you have this relationship or indicate none (add rows as needed)**	**Specifications/Comments (e.g., if payments were made to you or to your institution)**
**Time frame: Since the initial planning of the work**			
1	All support for the present manuscript (e.g., funding, provision of study materials, medical writing, article processing charges, etc.) **No time limit for this item.**	X	
**Time frame: past 36 months**			
2	Grants or contracts from any entity (if not indicated in item #1 above).	X	
3	Royalties or licenses	X	
4	Consulting fees	X	

5	Payment or honoraria for lectures, presentations, speakers bureaus, manuscript writing or educational events	X	
6	Payment for expert testimony	X	
7	Support for attending meetings and/or travel	X	
8	Patents planned, issued or pending	X	
9	Participation on a Data Safety Monitoring Board or Advisory Board	X	
10	Leadership or fiduciary role in other board, society, committee or advocacy group, paid or unpaid	X	
11	Stock or stock options	X	
12	Receipt of equipment, materials, drugs, medical writing, gifts or other services	X	
13	Other financial or non-financial interests	X	

Please place an "X" next to the following statement to indicate your agreement:

X I certify that I have answered every question and have not altered the wording of any of the questions on this form.

ICMJE DISCLOSURE FORM

Date:_14/11/2025
Your Name: Marion Limo
Manuscript Title: PEPITEM regulates the synovial microenvironment during immune-mediated inflammatory arthritis to limit disease
Manuscript number (if known): ar-25-0320

In the interest of transparency, we ask you to disclose all relationships/activities/interests listed below that are related to the content of your manuscript. "Related" means any relation with for-profit or not-for-profit third parties whose interests may be affected by the content of the manuscript. Disclosure represents a commitment to transparency and does not necessarily indicate a bias. If you are in doubt about whether to list a relationship/activity/interest, it is preferable that you do so.

The following questions apply to the author's relationships/activities/interests as they relate to the current manuscript only.

The author's relationships/activities/interests should be defined broadly. For example, if your manuscript pertains to the epidemiology of hypertension, you should declare all relationships with manufacturers of antihypertensive medication, even if that medication is not mentioned in the manuscript.

In item #1 below, report all support for the work reported in this manuscript without time limit. For all other items, the time frame for disclosure is the past 36 months.

		**Name all entities with whom you have this relationship or indicate none (add rows as needed)**	**Specifications/Comments (e.g., if payments were made to you or to your institution)**
**Time frame: Since the initial planning of the work**			
1	All support for the present manuscript (e.g., funding, provision of study materials, medical writing, article processing charges, etc.) **No time limit for this item.**	X	
**Time frame: past 36 months**			
2	Grants or contracts from any entity (if not indicated in item #1 above).	X	
3	Royalties or licenses	X	
4	Consulting fees	X	

5	Payment or honoraria for lectures, presentations, speakers bureaus, manuscript writing or educational events	X	
6	Payment for expert testimony	X	
7	Support for attending meetings and/or travel	X	
8	Patents planned, issued or pending	X	
9	Participation on a Data Safety Monitoring Board or Advisory Board	X	
10	Leadership or fiduciary role in other board, society, committee or advocacy group, paid or unpaid	X	
11	Stock or stock options	X	
12	Receipt of equipment, materials, drugs, medical writing, gifts or other services	X	
13	Other financial or non-financial interests	X	

Please place an "X" next to the following statement to indicate your agreement:

X I certify that I have answered every question and have not altered the wording of any of the questions on this form.

ICMJE DISCLOSURE FORM

Date:_14/11/2025
Your Name: Rakesh Jha
Manuscript Title: PEPITEM regulates the synovial microenvironment during immune-mediated inflammatory arthritis to limit disease
Manuscript number (if known): ar-25-0320

In the interest of transparency, we ask you to disclose all relationships/activities/interests listed below that are related to the content of your manuscript. "Related" means any relation with for-profit or not-for-profit third parties whose interests may be affected by the content of the manuscript. Disclosure represents a commitment to transparency and does not necessarily indicate a bias. If you are in doubt about whether to list a relationship/activity/interest, it is preferable that you do so.

The following questions apply to the author's relationships/activities/interests as they relate to the current manuscript only.

The author's relationships/activities/interests should be defined broadly. For example, if your manuscript pertains to the epidemiology of hypertension, you should declare all relationships with manufacturers of antihypertensive medication, even if that medication is not mentioned in the manuscript.

In item #1 below, report all support for the work reported in this manuscript without time limit. For all other items, the time frame for disclosure is the past 36 months.

		**Name all entities with whom you have this relationship or indicate none (add rows as needed)**	**Specifications/Comments (e.g., if payments were made to you or to your institution)**
		**Time frame: Since the initial planning of the work**	
1	All support for the present manuscript (e.g., funding, provision of study materials, medical writing, article processing charges, etc.) **No time limit for this item.**	X	
		**Time frame: past 36 months**	
2	Grants or contracts from any entity (if not indicated in item #1 above).	X	
3	Royalties or licenses	X	
4	Consulting fees	X	

5	Payment or honoraria for lectures, presentations, speakers bureaus, manuscript writing or educational events	X	
6	Payment for expert testimony	X	
7	Support for attending meetings and/or travel	X	
8	Patents planned, issued or pending	X	
9	Participation on a Data Safety Monitoring Board or Advisory Board	X	
10	Leadership or fiduciary role in other board, society, committee or advocacy group, paid or unpaid	X	
11	Stock or stock options	X	
12	Receipt of equipment, materials, drugs, medical writing, gifts or other services	X	
13	Other financial or non-financial interests	X	

Please place an "X" next to the following statement to indicate your agreement:

X I certify that I have answered every question and have not altered the wording of any of the questions on this form.

ICMJE DISCLOSURE FORM

Date:_14/11/2025
Your Name: Sandra Martinez Jarquin
Manuscript Title: PEPITEM regulates the synovial microenvironment during immune-mediated inflammatory arthritis to limit disease
Manuscript number (if known): ar-25-0320

In the interest of transparency, we ask you to disclose all relationships/activities/interests listed below that are related to the content of your manuscript. "Related" means any relation with for-profit or not-for-profit third parties whose interests may be affected by the content of the manuscript. Disclosure represents a commitment to transparency and does not necessarily indicate a bias. If you are in doubt about whether to list a relationship/activity/interest, it is preferable that you do so.

The following questions apply to the author's relationships/activities/interests as they relate to the current manuscript only.

The author's relationships/activities/interests should be defined broadly. For example, if your manuscript pertains to the epidemiology of hypertension, you should declare all relationships with manufacturers of antihypertensive medication, even if that medication is not mentioned in the manuscript.

In item #1 below, report all support for the work reported in this manuscript without time limit. For all other items, the time frame for disclosure is the past 36 months.

		**Name all entities with whom you have this relationship or indicate none (add rows as needed)**	**Specifications/Comments (e.g., if payments were made to you or to your institution)**
**Time frame: Since the initial planning of the work**			
1	All support for the present manuscript (e.g., funding, provision of study materials, medical writing, article processing charges, etc.) **No time limit for this item.**	X	
**Time frame: past 36 months**			
2	Grants or contracts from any entity (if not indicated in item #1 above).	X	
3	Royalties or licenses	X	
4	Consulting fees	X	

5	Payment or honoraria for lectures, presentations, speakers bureaus, manuscript writing or educational events	X	
6	Payment for expert testimony	X	
7	Support for attending meetings and/or travel	X	
8	Patents planned, issued or pending	X	
9	Participation on a Data Safety Monitoring Board or Advisory Board	X	
10	Leadership or fiduciary role in other board, society, committee or advocacy group, paid or unpaid	X	
11	Stock or stock options	X	
12	Receipt of equipment, materials, drugs, medical writing, gifts or other services	X	
13	Other financial or non-financial interests	X	

Please place an "X" next to the following statement to indicate your agreement:

X I certify that I have answered every question and have not altered the wording of any of the questions on this form.

ICMJE DISCLOSURE FORM

Date:_03/06/2025
Your Name: Noemi Marigliano
Manuscript Title: PEPITEM regulates the synovial microenvironment during immune-mediated inflammatory arthritis to limit disease
Manuscript number (if known): ar-25-0320

In the interest of transparency, we ask you to disclose all relationships/activities/interests listed below that are related to the content of your manuscript. "Related" means any relation with for-profit or not-for-profit third parties whose interests may be affected by the content of the manuscript. Disclosure represents a commitment to transparency and does not necessarily indicate a bias. If you are in doubt about whether to list a relationship/activity/interest, it is preferable that you do so.

The following questions apply to the author's relationships/activities/interests as they relate to the current manuscript only.

The author's relationships/activities/interests should be defined broadly. For example, if your manuscript pertains to the epidemiology of hypertension, you should declare all relationships with manufacturers of antihypertensive medication, even if that medication is not mentioned in the manuscript.

In item #1 below, report all support for the work reported in this manuscript without time limit. For all other items, the time frame for disclosure is the past 36 months.

		**Name all entities with whom you have this relationship or indicate none (add rows as needed)**	**Specifications/Comments (e.g., if payments were made to you or to your institution)**
**Time frame: Since the initial planning of the work**			
1	All support for the present manuscript (e.g., funding, provision of study materials, medical writing, article processing charges, etc.) **No time limit for this item.**	X	
**Time frame: past 36 months**			
2	Grants or contracts from any entity (if not indicated in item #1 above).	X	
3	Royalties or licenses	X	
4	Consulting fees	X	

5	Payment or honoraria for lectures, presentations, speakers bureaus, manuscript writing or educational events	X	
6	Payment for expert testimony	X	
7	Support for attending meetings and/or travel	X	
8	Patents planned, issued or pending	X	
9	Participation on a Data Safety Monitoring Board or Advisory Board	X	
10	Leadership or fiduciary role in other board, society, committee or advocacy group, paid or unpaid	X	
11	Stock or stock options	X	
12	Receipt of equipment, materials, drugs, medical writing, gifts or other services	X	
13	Other financial or non-financial interests	X	

Please place an "X" next to the following statement to indicate your agreement:

X I certify that I have answered every question and have not altered the wording of any of the questions on this form.

ICMJE DISCLOSURE FORM

Date:_03/06/2025
Your Name: Laleh Pezhman
Manuscript Title: PEPITEM regulates the synovial microenvironment during immune-mediated inflammatory arthritis to limit disease
Manuscript number (if known): ar-25-0320

In the interest of transparency, we ask you to disclose all relationships/activities/interests listed below that are related to the content of your manuscript. "Related" means any relation with for-profit or not-for-profit third parties whose interests may be affected by the content of the manuscript. Disclosure represents a commitment to transparency and does not necessarily indicate a bias. If you are in doubt about whether to list a relationship/activity/interest, it is preferable that you do so.

The following questions apply to the author's relationships/activities/interests as they relate to the current manuscript only.

The author's relationships/activities/interests should be defined broadly. For example, if your manuscript pertains to the epidemiology of hypertension, you should declare all relationships with manufacturers of antihypertensive medication, even if that medication is not mentioned in the manuscript.

In item #1 below, report all support for the work reported in this manuscript without time limit. For all other items, the time frame for disclosure is the past 36 months.

		**Name all entities with whom you have this relationship or indicate none (add rows as needed)**	**Specifications/Comments (e.g., if payments were made to you or to your institution)**
**Time frame: Since the initial planning of the work**			
1	All support for the present manuscript (e.g., funding, provision of study materials, medical writing, article processing charges, etc.) **No time limit for this item.**	X	
**Time frame: past 36 months**			
2	Grants or contracts from any entity (if not indicated in item #1 above).	X	
3	Royalties or licenses	X	
4	Consulting fees	X	

5	Payment or honoraria for lectures, presentations, speakers bureaus, manuscript writing or educational events	X	
6	Payment for expert testimony	X	
7	Support for attending meetings and/or travel	X	
8	Patents planned, issued or pending	X	
9	Participation on a Data Safety Monitoring Board or Advisory Board	X	
10	Leadership or fiduciary role in other board, society, committee or advocacy group, paid or unpaid	X	
11	Stock or stock options	X	
12	Receipt of equipment, materials, drugs, medical writing, gifts or other services	X	
13	Other financial or non-financial interests	X	

Please place an "X" next to the following statement to indicate your agreement:

X I certify that I have answered every question and have not altered the wording of any of the questions on this form.

ICMJE DISCLOSURE FORM

Date:_03/06/2025
Your Name: Abbie EA Degan
Manuscript Title: PEPITEM regulates the synovial microenvironment during immune-mediated inflammatory arthritis to limit disease
Manuscript number (if known): ar-25-0320

In the interest of transparency, we ask you to disclose all relationships/activities/interests listed below that are related to the content of your manuscript. "Related" means any relation with for-profit or not-for-profit third parties whose interests may be affected by the content of the manuscript. Disclosure represents a commitment to transparency and does not necessarily indicate a bias. If you are in doubt about whether to list a relationship/activity/interest, it is preferable that you do so.

The following questions apply to the author's relationships/activities/interests as they relate to the current manuscript only.

The author's relationships/activities/interests should be defined broadly. For example, if your manuscript pertains to the epidemiology of hypertension, you should declare all relationships with manufacturers of antihypertensive medication, even if that medication is not mentioned in the manuscript.

In item #1 below, report all support for the work reported in this manuscript without time limit. For all other items, the time frame for disclosure is the past 36 months.

		**Name all entities with whom you have this relationship or indicate none (add rows as needed)**	**Specifications/Comments (e.g., if payments were made to you or to your institution)**
**Time frame: Since the initial planning of the work**			
1	All support for the present manuscript (e.g., funding, provision of study materials, medical writing, article processing charges, etc.) **No time limit for this item.**	X	
**Time frame: past 36 months**			
2	Grants or contracts from any entity (if not indicated in item #1 above).	X	
3	Royalties or licenses	X	
4	Consulting fees	X	

5	Payment or honoraria for lectures, presentations, speakers bureaus, manuscript writing or educational events	X	
6	Payment for expert testimony	X	
7	Support for attending meetings and/or travel	X	
8	Patents planned, issued or pending	X	
9	Participation on a Data Safety Monitoring Board or Advisory Board	X	
10	Leadership or fiduciary role in other board, society, committee or advocacy group, paid or unpaid	X	
11	Stock or stock options	X	
12	Receipt of equipment, materials, drugs, medical writing, gifts or other services	X	
13	Other financial or non-financial interests	X	

Please place an "X" next to the following statement to indicate your agreement:

X I certify that I have answered every question and have not altered the wording of any of the questions on this form.

ICMJE DISCLOSURE FORM

Date:_03/06/2025__
Your Name:_Amy Anderson_______
Manuscript Title:_ PEPITEM regulates the synovial microenvironment during immune-mediated inflammatory arthritis to limit disease___
Manuscript number (if known):_ ar-25-0320_______________________________________

In the interest of transparency, we ask you to disclose all relationships/activities/interests listed below that are related to the content of your manuscript. "Related" means any relation with for-profit or not-for-profit third parties whose interests may be affected by the content of the manuscript. Disclosure represents a commitment to transparency and does not necessarily indicate a bias. If you are in doubt about whether to list a relationship/activity/interest, it is preferable that you do so.

The following questions apply to the author's relationships/activities/interests as they relate to the current manuscript only.

The author's relationships/activities/interests should be defined broadly. For example, if your manuscript pertains to the epidemiology of hypertension, you should declare all relationships with manufacturers of antihypertensive medication, even if that medication is not mentioned in the manuscript.

In item #1 below, report all support for the work reported in this manuscript without time limit. For all other items, the time frame for disclosure is the past 36 months.

		**Name all entities with whom you have this relationship or indicate none (add rows as needed)**	**Specifications/Comments (e.g., if payments were made to you or to your institution)**
**Time frame: Since the initial planning of the work**			
1	All support for the present manuscript (e.g., funding, provision of study materials, medical writing, article processing charges, etc.) **No time limit for this item.**	X	
2		X	

	Grants or contracts from any entity (if not indicated in item #1 above).		
3	Royalties or licenses	X	
4	Consulting fees	X	
5	Payment or honoraria for lectures, presentations, speakers bureaus, manuscript writing or educational events	X	
6	Payment for expert testimony	X	
7	Support for attending meetings and/or travel	X	
8	Patents planned, issued or pending	X	
9	Participation on a Data Safety Monitoring Board or Advisory Board	X	
10	Leadership or fiduciary role in other board, society, committee or advocacy group, paid or unpaid	X	
11	Stock or stock options	X	
12	Receipt of equipment, materials, drugs, medical writing, gifts or other services	X	
13	Other financial or non-financial interests	X	

Please place an "X" next to the following statement to indicate your agreement:

_X__ I certify that I have answered every question and have not altered the wording of any of the questions on this form.

ICMJE DISCLOSURE FORM

Date:_03/06/2025____
Your Name:__Charlotte G Smith____
Manuscript Title:_ PEPITEM regulates the synovial microenvironment during immune-mediated inflammatory arthritis to limit disease
Manuscript number (if known):__ ar-25-0320

In the interest of transparency, we ask you to disclose all relationships/activities/interests listed below that are related to the content of your manuscript. "Related" means any relation with for-profit or not-for-profit third parties whose interests may be affected by the content of the manuscript. Disclosure represents a commitment to transparency and does not necessarily indicate a bias. If you are in doubt about whether to list a relationship/activity/interest, it is preferable that you do so.

The following questions apply to the author's relationships/activities/interests as they relate to the <u>current manuscript only</u>.

The author's relationships/activities/interests should be <u>defined broadly</u>. For example, if your manuscript pertains to the epidemiology of hypertension, you should declare all relationships with manufacturers of antihypertensive medication, even if that medication is not mentioned in the manuscript.

In item #1 below, report all support for the work reported in this manuscript without time limit. For all other items, the time frame for disclosure is the past 36 months.

		**Name all entities with whom you have this relationship or indicate none (add rows as needed)**	**Specifications/Comments (e.g., if payments were made to you or to your institution)**
		**Time frame: Since the initial planning of the work**	
1	All support for the present manuscript (e.g., funding, provision of study materials, medical writing, article processing charges, etc.) **No time limit for this item.**	X	N/A
		**Time frame: past 36 months**	
2	Grants or contracts from any entity (if not indicated in item #1 above).	X	N/A
3	Royalties or licenses	X	N/A
4	Consulting fees	X	N/A

5	Payment or honoraria for lectures, presentations, speakers bureaus, manuscript writing or educational events	X	N/A
6	Payment for expert testimony	X	N/A
7	Support for attending meetings and/or travel	X	N/A
8	Patents planned, issued or pending	X	N/A
9	Participation on a Data Safety Monitoring Board or Advisory Board	X	N/A
10	Leadership or fiduciary role in other board, society, committee or advocacy group, paid or unpaid	X	N/A
11	Stock or stock options	X	N/A
12	Receipt of equipment, materials, drugs, medical writing, gifts or other services	X	N/A
13	Other financial or non-financial interests	X	N/A

Please place an "X" next to the following statement to indicate your agreement:

__X_ I certify that I have answered every question and have not altered the wording of any of the questions on this form.

ICMJE DISCLOSURE FORM

Date:_03/06/2025____
Your Name:__Armaiti Batki
Manuscript Title:_ PEPITEM regulates the synovial microenvironment during immune-mediated inflammatory arthritis to limit disease
Manuscript number (if known):__ ar-25-0320

In the interest of transparency, we ask you to disclose all relationships/activities/interests listed below that are related to the content of your manuscript. "Related" means any relation with for-profit or not-for-profit third parties whose interests may be affected by the content of the manuscript. Disclosure represents a commitment to transparency and does not necessarily indicate a bias. If you are in doubt about whether to list a relationship/activity/interest, it is preferable that you do so.

The following questions apply to the author's relationships/activities/interests as they relate to the current manuscript only.

The author's relationships/activities/interests should be defined broadly. For example, if your manuscript pertains to the epidemiology of hypertension, you should declare all relationships with manufacturers of antihypertensive medication, even if that medication is not mentioned in the manuscript.

In item #1 below, report all support for the work reported in this manuscript without time limit. For all other items, the time frame for disclosure is the past 36 months.

		**Name all entities with whom you have this relationship or indicate none (add rows as needed)**	**Specifications/Comments (e.g., if payments were made to you or to your institution)**
		**Time frame: Since the initial planning of the work**	
1	All support for the present manuscript (e.g., funding, provision of study materials, medical writing, article processing charges, etc.) **No time limit for this item.**	X	N/A
		**Time frame: past 36 months**	
2	Grants or contracts from any entity (if not indicated in item #1 above).	X	N/A
3	Royalties or licenses	X	N/A
4	Consulting fees	X	N/A

5	Payment or honoraria for lectures, presentations, speakers bureaus, manuscript writing or educational events	X	N/A
6	Payment for expert testimony	X	N/A
7	Support for attending meetings and/or travel	X	N/A
8	Patents planned, issued or pending	X	N/A
9	Participation on a Data Safety Monitoring Board or Advisory Board	X	N/A
10	Leadership or fiduciary role in other board, society, committee or advocacy group, paid or unpaid	X	N/A
11	Stock or stock options	X	N/A
12	Receipt of equipment, materials, drugs, medical writing, gifts or other services	X	N/A
13	Other financial or non-financial interests	X	N/A

Please place an "X" next to the following statement to indicate your agreement:

__X_ I certify that I have answered every question and have not altered the wording of any of the questions on this form.

ICMJE DISCLOSURE FORM

Date:_15/07/2025____
Your Name:__Holly Adams
Manuscript Title:_ PEPITEM regulates the synovial microenvironment during immune-mediated inflammatory arthritis to limit disease
Manuscript number (if known):__ ar-25-0320

In the interest of transparency, we ask you to disclose all relationships/activities/interests listed below that are related to the content of your manuscript. "Related" means any relation with for-profit or not-for-profit third parties whose interests may be affected by the content of the manuscript. Disclosure represents a commitment to transparency and does not necessarily indicate a bias. If you are in doubt about whether to list a relationship/activity/interest, it is preferable that you do so.

The following questions apply to the author's relationships/activities/interests as they relate to the current manuscript only.

The author's relationships/activities/interests should be defined broadly. For example, if your manuscript pertains to the epidemiology of hypertension, you should declare all relationships with manufacturers of antihypertensive medication, even if that medication is not mentioned in the manuscript.

In item #1 below, report all support for the work reported in this manuscript without time limit. For all other items, the time frame for disclosure is the past 36 months.

		**Name all entities with whom you have this relationship or indicate none (add rows as needed)**	**Specifications/Comments (e.g., if payments were made to you or to your institution)**
**Time frame: Since the initial planning of the work**			
1	All support for the present manuscript (e.g., funding, provision of study materials, medical writing, article processing charges, etc.) **No time limit for this item.**	X	N/A
**Time frame: past 36 months**			
2	Grants or contracts from any entity (if not indicated in item #1 above).	X	N/A
3	Royalties or licenses	X	N/A
4	Consulting fees	X	N/A

5	Payment or honoraria for lectures, presentations, speakers bureaus, manuscript writing or educational events	X	N/A
6	Payment for expert testimony	X	N/A
7	Support for attending meetings and/or travel	X	N/A
8	Patents planned, issued or pending	X	N/A
9	Participation on a Data Safety Monitoring Board or Advisory Board	X	N/A
10	Leadership or fiduciary role in other board, society, committee or advocacy group, paid or unpaid	X	N/A
11	Stock or stock options	X	N/A
12	Receipt of equipment, materials, drugs, medical writing, gifts or other services	X	N/A
13	Other financial or non-financial interests	X	N/A

Please place an "X" next to the following statement to indicate your agreement:

__X_ I certify that I have answered every question and have not altered the wording of any of the questions on this form.

Date:_15/07/2025
Your Name: Francesco Caso
Manuscript Title: PEPITEM regulates the synovial microenvironment during immune-mediated inflammatory arthritis to limit disease
Manuscript number (if known): ar-25-0320

In the interest of transparency, we ask you to disclose all relationships/activities/interests listed below that are related to the content of your manuscript. "Related" means any relation with for-profit or not-for-profit third parties whose interests may be affected by the content of the manuscript. Disclosure represents a commitment to transparency and does not necessarily indicate a bias. If you are in doubt about whether to list a relationship/activity/interest, it is preferable that you do so.

The following questions apply to the author's relationships/activities/interests as they relate to the current manuscript only.

The author's relationships/activities/interests should be defined broadly. For example, if your manuscript pertains to the epidemiology of hypertension, you should declare all relationships with manufacturers of antihypertensive medication, even if that medication is not mentioned in the manuscript.

In item #1 below, report all support for the work reported in this manuscript without time limit. For all other items, the time frame for disclosure is the past 36 months.

		**Name all entities with whom you have this relationship or indicate none (add rows as needed)**	**Specifications/Comments (e.g., if payments were made to you or to your institution)**
		**Time frame: Since the initial planning of the work**	
1	All support for the present manuscript (e.g., funding, provision of study materials, medical writing, article processing charges, etc.) **No time limit for this item.**	X	
		**Time frame: past 36 months**	
2	Grants or contracts from any entity (if not indicated in item #1 above).	X	
3	Royalties or licenses	X	
4	Consulting fees	X	
5		X	

	Payment or honoraria for lectures, presentations, speakers bureaus, manuscript writing or educational events		
6	Payment for expert testimony	X	
7	Support for attending meetings and/or travel	X	
8	Patents planned, issued or pending	X	
9	Participation on a Data Safety Monitoring Board or Advisory Board	X	
10	Leadership or fiduciary role in other board, society, committee or advocacy group, paid or unpaid	X	
11	Stock or stock options	X	
12	Receipt of equipment, materials, drugs, medical writing, gifts or other services	X	
13	Other financial or non-financial interests	X	

Please place an "X" next to the following statement to indicate your agreement:

X I certify that I have answered every question and have not altered the wording of any of the questions on this form.

Date:_15/07/2025
Your Name: Raffaele Scarpa
Manuscript Title: PEPITEM regulates the synovial microenvironment during immune-mediated inflammatory arthritis to limit disease
Manuscript number (if known): ar-25-0320

In the interest of transparency, we ask you to disclose all relationships/activities/interests listed below that are related to the content of your manuscript. "Related" means any relation with for-profit or not-for-profit third parties whose interests may be affected by the content of the manuscript. Disclosure represents a commitment to transparency and does not necessarily indicate a bias. If you are in doubt about whether to list a relationship/activity/interest, it is preferable that you do so.

The following questions apply to the author's relationships/activities/interests as they relate to the current manuscript only.

The author's relationships/activities/interests should be defined broadly. For example, if your manuscript pertains to the epidemiology of hypertension, you should declare all relationships with manufacturers of antihypertensive medication, even if that medication is not mentioned in the manuscript.

In item #1 below, report all support for the work reported in this manuscript without time limit. For all other items, the time frame for disclosure is the past 36 months.

		**Name all entities with whom you have this relationship or indicate none (add rows as needed)**	**Specifications/Comments (e.g., if payments were made to you or to your institution)**
		**Time frame: Since the initial planning of the work**	
1	All support for the present manuscript (e.g., funding, provision of study materials, medical writing, article processing charges, etc.) **No time limit for this item.**	X	
		**Time frame: past 36 months**	
2	Grants or contracts from any entity (if not indicated in item #1 above).	X	
3	Royalties or licenses	X	
4	Consulting fees	X	
5		X	

	Payment or honoraria for lectures, presentations, speakers bureaus, manuscript writing or educational events		
6	Payment for expert testimony	X	
7	Support for attending meetings and/or travel	X	
8	Patents planned, issued or pending	X	
9	Participation on a Data Safety Monitoring Board or Advisory Board	X	
10	Leadership or fiduciary role in other board, society, committee or advocacy group, paid or unpaid	X	
11	Stock or stock options	X	
12	Receipt of equipment, materials, drugs, medical writing, gifts or other services	X	
13	Other financial or non-financial interests	X	

Please place an "X" next to the following statement to indicate your agreement:

X I certify that I have answered every question and have not altered the wording of any of the questions on this form.

Date:_15/07/2025
Your Name: Iain McInnes
Manuscript Title: PEPITEM regulates the synovial microenvironment during immune-mediated inflammatory arthritis to limit disease
Manuscript number (if known): ar-25-0320

In the interest of transparency, we ask you to disclose all relationships/activities/interests listed below that are related to the content of your manuscript. "Related" means any relation with for-profit or not-for-profit third parties whose interests may be affected by the content of the manuscript. Disclosure represents a commitment to transparency and does not necessarily indicate a bias. If you are in doubt about whether to list a relationship/activity/interest, it is preferable that you do so.

The following questions apply to the author's relationships/activities/interests as they relate to the current manuscript only.

The author's relationships/activities/interests should be defined broadly. For example, if your manuscript pertains to the epidemiology of hypertension, you should declare all relationships with manufacturers of antihypertensive medication, even if that medication is not mentioned in the manuscript.

In item #1 below, report all support for the work reported in this manuscript without time limit. For all other items, the time frame for disclosure is the past 36 months.

		**Name all entities with whom you have this relationship or indicate none (add rows as needed)**	**Specifications/Comments (e.g., if payments were made to you or to your institution)**
**Time frame: Since the initial planning of the work**			
1	All support for the present manuscript (e.g., funding, provision of study materials, medical writing, article processing charges, etc.) **No time limit for this item.**	X	
**Time frame: past 36 months**			
2	Grants or contracts from any entity (if not indicated in item #1 above).	X	
3	Royalties or licenses	X	
4	Consulting fees	X	
5		X	

	Payment or honoraria for lectures, presentations, speakers bureaus, manuscript writing or educational events		
6	Payment for expert testimony	X	
7	Support for attending meetings and/or travel	X	
8	Patents planned, issued or pending	X	
9	Participation on a Data Safety Monitoring Board or Advisory Board	X	
10	Leadership or fiduciary role in other board, society, committee or advocacy group, paid or unpaid	X	
11	Stock or stock options	X	
12	Receipt of equipment, materials, drugs, medical writing, gifts or other services	X	
13	Other financial or non-financial interests	X	

Please place an "X" next to the following statement to indicate your agreement:

X I certify that I have answered every question and have not altered the wording of any of the questions on this form.

ICMJE DISCLOSURE FORM

Date: 08/06/2025
Your Name: Stefan Siebert
Manuscript Title: PEPITEM regulates the synovial microenvironment during immune-mediated inflammatory arthritis to limit disease.
Manuscript number (if known):____

In the interest of transparency, we ask you to disclose all relationships/activities/interests listed below that are related to the content of your manuscript. "Related" means any relation with for-profit or not-for-profit third parties whose interests may be affected by the content of the manuscript. Disclosure represents a commitment to transparency and does not necessarily indicate a bias. If you are in doubt about whether to list a relationship/activity/interest, it is preferable that you do so.

The following questions apply to the author's relationships/activities/interests as they relate to the current manuscript only.

The author's relationships/activities/interests should be defined broadly. For example, if your manuscript pertains to the epidemiology of hypertension, you should declare all relationships with manufacturers of antihypertensive medication, even if that medication is not mentioned in the manuscript.

In item #1 below, report all support for the work reported in this manuscript without time limit. For all other items, the time frame for disclosure is the past 36 months.

		Name all entities with whom you have this relationship or indicate none (add rows as needed)	Specifications/Comments (e.g., if payments were made to you or to your institution)
**Time frame: Since the initial planning of the work**			
1	All support for the present manuscript (e.g., funding, provision of study materials, medical writing, article processing charges, etc.) No time limit for this item.	☒ None	
**Time frame: past 36 months**			
2	Grants or contracts from any entity (if not indicated in item #1 above).	☒ None	

3	Royalties or licenses	☒ None	
4	Consulting fees	☒ None	
5	Payment or honoraria for lectures, presentations, speakers bureaus, manuscript writing or educational events	☒ None	
6	Payment for expert testimony	☒ None	
7	Support for attending meetings and/or travel	☒ None	
8	Patents planned, issued or pending	☒ None	
9	Participation on a Data Safety Monitoring Board or Advisory Board	☒ None	
10	Leadership or fiduciary role in other board, society, committee or advocacy group, paid or unpaid	☒ None	
11	Stock or stock options	☒ None	
12	Receipt of equipment, materials, drugs, medical writing, gifts or other services	☒ None	
13	Other financial or non-financial interests	☒ None	

Please place an "X" next to the following statement to indicate your agreement:

X_ I certify that I have answered every question and have not altered the wording of any of the questions on this form.

ICMJE DISCLOSURE FORM

Date: 03-JUN-2025
Your Name: Arthur G Pratt
Manuscript Title: PEPITEM regulates the synovial microenvironment during immune-mediated inflammatory arthritis to limit disease.
Manuscript number (if known): ar-25-0320

In the interest of transparency, we ask you to disclose all relationships/activities/interests listed below that are related to the content of your manuscript. "Related" means any relation with for-profit or not-for-profit third parties whose interests may be affected by the content of the manuscript. Disclosure represents a commitment to transparency and does not necessarily indicate a bias. If you are in doubt about whether to list a relationship/activity/interest, it is preferable that you do so.

The following questions apply to the author's relationships/activities/interests as they relate to the current manuscript only.

The author's relationships/activities/interests should be defined broadly. For example, if your manuscript pertains to the epidemiology of hypertension, you should declare all relationships with manufacturers of antihypertensive medication, even if that medication is not mentioned in the manuscript.

In item #1 below, report all support for the work reported in this manuscript without time limit. For all other items, the time frame for disclosure is the past 36 months.

		**Name all entities with whom you have this relationship or indicate none (add rows as needed)**	**Specifications/Comments (e.g., if payments were made to you or to your institution)**
**Time frame: Since the initial planning of the work**			
1	All support for the present manuscript (e.g., funding, provision of study materials, medical writing, article processing charges, etc.) **No time limit for this item.**	Versus Arthritis	Research into Inflammatory Arthritis Centre Versus Arthritis (RACE) (grant number 22072)
		National Institute of Health and Care Research	NIHR Newcastle Biomedical Research Centre
**Time frame: past 36 months**			
2	Grants or contracts from any entity (if not indicated in item #1 above).	GSK	In-kind payment to Newcastle University as part of EMINENT (GSK/MRC) funding award.
3	Royalties or licenses	X	

4	Consulting fees	X	
5	Payment or honoraria for lectures, presentations, speakers bureaus, manuscript writing or educational events	X	
6	Payment for expert testimony	X	
7	Support for attending meetings and/or travel	X	
8	Patents planned, issued or pending	X	
9	Participation on a Data Safety Monitoring Board or Advisory Board	X	
10	Leadership or fiduciary role in other board, society, committee or advocacy group, paid or unpaid	X	
11	Stock or stock options	X	
12	Receipt of equipment, materials, drugs, medical writing, gifts or other services	X	
13	Other financial or non-financial interests	X	

Please place an "X" next to the following statement to indicate your agreement:

X I certify that I have answered every question and have not altered the wording of any of the questions on this form.

ICMJE DISCLOSURE FORM

Date: 15.07.2025
Your Name: Andrew Filer
Manuscript Title: PEPITEM regulates the synovial microenvironment during immune-mediated inflammatory arthritis to limit disease
Manuscript number (if known): ar-25-0320

In the interest of transparency, we ask you to disclose all relationships/activities/interests listed below that are related to the content of your manuscript. "Related" means any relation with for-profit or not-for-profit third parties whose interests may be affected by the content of the manuscript. Disclosure represents a commitment to transparency and does not necessarily indicate a bias. If you are in doubt about whether to list a relationship/activity/interest, it is preferable that you do so.

The following questions apply to the author's relationships/activities/interests as they relate to the current manuscript only.

The author's relationships/activities/interests should be defined broadly. For example, if your manuscript pertains to the epidemiology of hypertension, you should declare all relationships with manufacturers of antihypertensive medication, even if that medication is not mentioned in the manuscript.

In item #1 below, report all support for the work reported in this manuscript without time limit. For all other items, the time frame for disclosure is the past 36 months.

		**Name all entities with whom you have this relationship or indicate none (add rows as needed)**	**Specifications/Comments (e.g., if payments were made to you or to your institution)**
		**Time frame: Since the initial planning of the work**	
1	All support for the present manuscript (e.g., funding, provision of study materials, medical writing, article processing charges, etc.) **No time limit for this item.**	MRC	Grant paid to the University
		Research into Inflammatory Arthritis Centre Versus Arthritis (RACE)	Grant paid to the University
		NIHR Birmingham Biomedical Research Centres	Grant paid to the University
		**Time frame: past 36 months**	
2	Grants or contracts from any entity (if not indicated in item #1 above).	X	

3	Royalties or licenses	X	
4	Consulting fees	X	
5	Payment or honoraria for lectures, presentations, speakers bureaus, manuscript writing or educational events	X	
6	Payment for expert testimony	X	
7	Support for attending meetings and/or travel	X	
8	Patents planned, issued or pending	X	
9	Participation on a Data Safety Monitoring Board or Advisory Board	X	
10	Leadership or fiduciary role in other board, society, committee or advocacy group, paid or unpaid	X	
11	Stock or stock options	X	
12	Receipt of equipment, materials, drugs, medical writing, gifts or other services	X	
13	Other financial or non-financial interests	X	

Please place an "X" next to the following statement to indicate your agreement:

_X_ I certify that I have answered every question and have not altered the wording of any of the questions on this form.

ICMJE DISCLOSURE FORM

Date: 04.06.2025
Your Name: Karim Raza
Manuscript Title: PEPITEM regulates the synovial microenvironment during immune-mediated inflammatory arthritis to limit disease
Manuscript number (if known): ar-25-0320

In the interest of transparency, we ask you to disclose all relationships/activities/interests listed below that are related to the content of your manuscript. "Related" means any relation with for-profit or not-for-profit third parties whose interests may be affected by the content of the manuscript. Disclosure represents a commitment to transparency and does not necessarily indicate a bias. If you are in doubt about whether to list a relationship/activity/interest, it is preferable that you do so.

The following questions apply to the author's relationships/activities/interests as they relate to the <u>current manuscript only</u>.

The author's relationships/activities/interests should be <u>defined broadly</u>. For example, if your manuscript pertains to the epidemiology of hypertension, you should declare all relationships with manufacturers of antihypertensive medication, even if that medication is not mentioned in the manuscript.

In item #1 below, report all support for the work reported in this manuscript without time limit. For all other items, the time frame for disclosure is the past 36 months.

		**Name all entities with whom you have this relationship or indicate none (add rows as needed)**	**Specifications/Comments (e.g., if payments were made to you or to your institution)**
		**Time frame: Since the initial planning of the work**	
1	All support for the present manuscript (e.g., funding, provision of study materials, medical writing, article processing charges, etc.) **No time limit for this item.**	MRC	Grant paid to the University
		Research into Inflammatory Arthritis Centre Versus Arthritis (RACE)	Grant paid to the University
		NIHR Birmingham Biomedical Research Centres	Grant paid to the University
		**Time frame: past 36 months**	
2	Grants or contracts from any entity (if not indicated in item #1 above).	X	

3	Royalties or licenses	X	
4	Consulting fees	X	
5	Payment or honoraria for lectures, presentations, speakers bureaus, manuscript writing or educational events	X	
6	Payment for expert testimony	X	
7	Support for attending meetings and/or travel	X	
8	Patents planned, issued or pending	X	
9	Participation on a Data Safety Monitoring Board or Advisory Board	X	
10	Leadership or fiduciary role in other board, society, committee or advocacy group, paid or unpaid	X	
11	Stock or stock options	X	
12	Receipt of equipment, materials, drugs, medical writing, gifts or other services	X	
13	Other financial or non-financial interests	X	

Please place an "X" next to the following statement to indicate your agreement:

_X_ I certify that I have answered every question and have not altered the wording of any of the questions on this form.

ICMJE DISCLOSURE FORM

Date:_14/11/2025
Your Name: Adam P Croft
Manuscript Title: PEPITEM regulates the synovial microenvironment during immune-mediated inflammatory arthritis to limit disease
Manuscript number (if known): ar-25-0320

In the interest of transparency, we ask you to disclose all relationships/activities/interests listed below that are related to the content of your manuscript. "Related" means any relation with for-profit or not-for-profit third parties whose interests may be affected by the content of the manuscript. Disclosure represents a commitment to transparency and does not necessarily indicate a bias. If you are in doubt about whether to list a relationship/activity/interest, it is preferable that you do so.

The following questions apply to the author's relationships/activities/interests as they relate to the current manuscript only.

The author's relationships/activities/interests should be defined broadly. For example, if your manuscript pertains to the epidemiology of hypertension, you should declare all relationships with manufacturers of antihypertensive medication, even if that medication is not mentioned in the manuscript.

In item #1 below, report all support for the work reported in this manuscript without time limit. For all other items, the time frame for disclosure is the past 36 months.

		**Name all entities with whom you have this relationship or indicate none (add rows as needed)**	**Specifications/Comments (e.g., if payments were made to you or to your institution)**
**Time frame: Since the initial planning of the work**			
1	All support for the present manuscript (e.g., funding, provision of study materials, medical writing, article processing charges, etc.) **No time limit for this item.**	X	
**Time frame: past 36 months**			
2	Grants or contracts from any entity (if not indicated in item #1 above).	X	
3	Royalties or licenses	X	
4	Consulting fees	X	

5	Payment or honoraria for lectures, presentations, speakers bureaus, manuscript writing or educational events	X	
6	Payment for expert testimony	X	
7	Support for attending meetings and/or travel	X	
8	Patents planned, issued or pending	X	
9	Participation on a Data Safety Monitoring Board or Advisory Board	X	
10	Leadership or fiduciary role in other board, society, committee or advocacy group, paid or unpaid	X	
11	Stock or stock options	X	
12	Receipt of equipment, materials, drugs, medical writing, gifts or other services	X	
13	Other financial or non-financial interests	X	

Please place an "X" next to the following statement to indicate your agreement:

_X_ I certify that I have answered every question and have not altered the wording of any of the questions on this form.

ICMJE DISCLOSURE FORM

Date:_14/11/2025
Your Name: Myriam Chimen
Manuscript Title: PEPITEM regulates the synovial microenvironment during immune-mediated inflammatory arthritis to limit disease
Manuscript number (if known): ar-25-0320

In the interest of transparency, we ask you to disclose all relationships/activities/interests listed below that are related to the content of your manuscript. "Related" means any relation with for-profit or not-for-profit third parties whose interests may be affected by the content of the manuscript. Disclosure represents a commitment to transparency and does not necessarily indicate a bias. If you are in doubt about whether to list a relationship/activity/interest, it is preferable that you do so.

The following questions apply to the author's relationships/activities/interests as they relate to the current manuscript only.

The author's relationships/activities/interests should be defined broadly. For example, if your manuscript pertains to the epidemiology of hypertension, you should declare all relationships with manufacturers of antihypertensive medication, even if that medication is not mentioned in the manuscript.

In item #1 below, report all support for the work reported in this manuscript without time limit. For all other items, the time frame for disclosure is the past 36 months.

		**Name all entities with whom you have this relationship or indicate none (add rows as needed)**	**Specifications/Comments (e.g., if payments were made to you or to your institution)**
**Time frame: Since the initial planning of the work**			
1	All support for the present manuscript (e.g., funding, provision of study materials, medical writing, article processing charges, etc.) **No time limit for this item.**	X	
**Time frame: past 36 months**			
2	Grants or contracts from any entity (if not indicated in item #1 above).	X	
3	Royalties or licenses	X	
4	Consulting fees	X	

5	Payment or honoraria for lectures, presentations, speakers bureaus, manuscript writing or educational events	X	
6	Payment for expert testimony	X	
7	Support for attending meetings and/or travel	X	
8	Patents planned, issued or pending	X	
9	Participation on a Data Safety Monitoring Board or Advisory Board	X	
10	Leadership or fiduciary role in other board, society, committee or advocacy group, paid or unpaid	X	
11	Stock or stock options	X	
12	Receipt of equipment, materials, drugs, medical writing, gifts or other services	X	
13	Other financial or non-financial interests	X	

Please place an "X" next to the following statement to indicate your agreement:

_X_ I certify that I have answered every question and have not altered the wording of any of the questions on this form.

ICMJE DISCLOSURE FORM

Date:_14/11/2025
Your Name: Felicity de Cogan
Manuscript Title: PEPITEM regulates the synovial microenvironment during immune-mediated inflammatory arthritis to limit disease
Manuscript number (if known): ar-25-0320

In the interest of transparency, we ask you to disclose all relationships/activities/interests listed below that are related to the content of your manuscript. "Related" means any relation with for-profit or not-for-profit third parties whose interests may be affected by the content of the manuscript. Disclosure represents a commitment to transparency and does not necessarily indicate a bias. If you are in doubt about whether to list a relationship/activity/interest, it is preferable that you do so.

The following questions apply to the author's relationships/activities/interests as they relate to the current manuscript only.

The author's relationships/activities/interests should be defined broadly. For example, if your manuscript pertains to the epidemiology of hypertension, you should declare all relationships with manufacturers of antihypertensive medication, even if that medication is not mentioned in the manuscript.

In item #1 below, report all support for the work reported in this manuscript without time limit. For all other items, the time frame for disclosure is the past 36 months.

		**Name all entities with whom you have this relationship or indicate none (add rows as needed)**	**Specifications/Comments (e.g., if payments were made to you or to your institution)**
**Time frame: Since the initial planning of the work**			
1	All support for the present manuscript (e.g., funding, provision of study materials, medical writing, article processing charges, etc.) **No time limit for this item.**	X	
**Time frame: past 36 months**			
2	Grants or contracts from any entity (if not indicated in item #1 above).	X	
3	Royalties or licenses	X	
4	Consulting fees	X	

5	Payment or honoraria for lectures, presentations, speakers bureaus, manuscript writing or educational events	X	
6	Payment for expert testimony	X	
7	Support for attending meetings and/or travel	X	
8	Patents planned, issued or pending	X	
9	Participation on a Data Safety Monitoring Board or Advisory Board	X	
10	Leadership or fiduciary role in other board, society, committee or advocacy group, paid or unpaid	X	
11	Stock or stock options	X	
12	Receipt of equipment, materials, drugs, medical writing, gifts or other services	X	
13	Other financial or non-financial interests	X	

Please place an "X" next to the following statement to indicate your agreement:

_X_ I certify that I have answered every question and have not altered the wording of any of the questions on this form.

ICMJE DISCLOSURE FORM

Date:_______03/06/2025____
Your Name: ____George Edward Rainger____
Manuscript Title:____
Manuscript number (if known):______ ar-25-0320 ____

In the interest of transparency, we ask you to disclose all relationships/activities/interests listed below that are related to the content of your manuscript. "Related" means any relation with for-profit or not-for-profit third parties whose interests may be affected by the content of the manuscript. Disclosure represents a commitment to transparency and does not necessarily indicate a bias. If you are in doubt about whether to list a relationship/activity/interest, it is preferable that you do so.

The following questions apply to the author's relationships/activities/interests as they relate to the current manuscript only.

The author's relationships/activities/interests should be defined broadly. For example, if your manuscript pertains to the epidemiology of hypertension, you should declare all relationships with manufacturers of antihypertensive medication, even if that medication is not mentioned in the manuscript.

In item #1 below, report all support for the work reported in this manuscript without time limit. For all other items, the time frame for disclosure is the past 36 months.

		**Name all entities with whom you have this relationship or indicate none (add rows as needed)**	**Specifications/Comments (e.g., if payments were made to you or to your institution)**
		**Time frame: Since the initial planning of the work**	
1	All support for the present manuscript (e.g., funding, provision of study materials, medical writing, article processing charges, etc.) **No time limit for this item.**	MRC	Grant paid to the University
		NIHR Birmingham Biomedical Research Centres	Grant paid to the University
		**Time frame: past 36 months**	
2	Grants or contracts from any entity (if not indicated in item #1 above).	X	
3	Royalties or licenses	X	
4	Consulting fees	X	

5	Payment or honoraria for lectures, presentations, speakers bureaus, manuscript writing or educational events	X	
6	Payment for expert testimony	X	
7	Support for attending meetings and/or travel	X	
8	Patents planned, issued or pending	Patents	Composition of matter patent for PEPITEM awarded to University of Birmingham
9	Participation on a Data Safety Monitoring Board or Advisory Board	X	
10	Leadership or fiduciary role in other board, society, committee or advocacy group, paid or unpaid	X	
11	Stock or stock options	X	
12	Receipt of equipment, materials, drugs, medical writing, gifts or other services	X	
13	Other financial or non-financial interests	X	

Please place an "X" next to the following statement to indicate your agreement:

__X_ I certify that I have answered every question and have not altered the wording of any of the questions on this form.

ICMJE DISCLOSURE FORM

Date: 04.06.2025
Your Name: Asif J Iqbal
Manuscript Title: PEPITEM regulates the synovial microenvironment during immune-mediated inflammatory arthritis to limit disease
Manuscript number (if known): ar-25-0320

In the interest of transparency, we ask you to disclose all relationships/activities/interests listed below that are related to the content of your manuscript. "Related" means any relation with for-profit or not-for-profit third parties whose interests may be affected by the content of the manuscript. Disclosure represents a commitment to transparency and does not necessarily indicate a bias. If you are in doubt about whether to list a relationship/activity/interest, it is preferable that you do so.

The following questions apply to the author's relationships/activities/interests as they relate to the current manuscript only.

The author's relationships/activities/interests should be defined broadly. For example, if your manuscript pertains to the epidemiology of hypertension, you should declare all relationships with manufacturers of antihypertensive medication, even if that medication is not mentioned in the manuscript.

In item #1 below, report all support for the work reported in this manuscript without time limit. For all other items, the time frame for disclosure is the past 36 months.

		**Name all entities with whom you have this relationship or indicate none (add rows as needed)**	**Specifications/Comments (e.g., if payments were made to you or to your institution)**
**Time frame: Since the initial planning of the work**			
1	All support for the present manuscript (e.g., funding, provision of study materials, medical writing, article processing charges, etc.) **No time limit for this item.**	NIHR Birmingham Biomedical Research Centres	Grant paid to the University
**Time frame: past 36 months**			
2	Grants or contracts from any entity (if not indicated in item #1 above).	X	
3	Royalties or licenses	X	

4	Consulting fees	X	
5	Payment or honoraria for lectures, presentations, speakers bureaus, manuscript writing or educational events	X	
6	Payment for expert testimony	X	
7	Support for attending meetings and/or travel	X	
8	Patents planned, issued or pending	Patents	Composition of matter patent for PEPITEM awarded to University of Birmingham
9	Participation on a Data Safety Monitoring Board or Advisory Board	X	
10	Leadership or fiduciary role in other board, society, committee or advocacy group, paid or unpaid	X	
11	Stock or stock options	X	
12	Receipt of equipment, materials, drugs, medical writing, gifts or other services	X	
13	Other financial or non-financial interests	X	

Please place an "X" next to the following statement to indicate your agreement:

_X_ I certify that I have answered every question and have not altered the wording of any of the questions on this form.

ICMJE DISCLOSURE FORM

Date:_03/06/2025
Your Name: Francesco Maione
Manuscript Title: PEPITEM regulates the synovial microenvironment during immune-mediated inflammatory arthritis to limit disease
Manuscript number (if known): ar-25-0320

In the interest of transparency, we ask you to disclose all relationships/activities/interests listed below that are related to the content of your manuscript. "Related" means any relation with for-profit or not-for-profit third parties whose interests may be affected by the content of the manuscript. Disclosure represents a commitment to transparency and does not necessarily indicate a bias. If you are in doubt about whether to list a relationship/activity/interest, it is preferable that you do so.

The following questions apply to the author's relationships/activities/interests as they relate to the current manuscript only.

The author's relationships/activities/interests should be defined broadly. For example, if your manuscript pertains to the epidemiology of hypertension, you should declare all relationships with manufacturers of antihypertensive medication, even if that medication is not mentioned in the manuscript.

In item #1 below, report all support for the work reported in this manuscript without time limit. For all other items, the time frame for disclosure is the past 36 months.

		**Name all entities with whom you have this relationship or indicate none (add rows as needed)**	**Specifications/Comments (e.g., if payments were made to you or to your institution)**
		**Time frame: Since the initial planning of the work**	
1	All support for the present manuscript (e.g., funding, provision of study materials, medical writing, article processing charges, etc.) **No time limit for this item.**	X	
		**Time frame: past 36 months**	
2	Grants or contracts from any entity (if not indicated in item #1 above).	X	
3	Royalties or licenses	X	
4	Consulting fees	X	

5	Payment or honoraria for lectures, presentations, speakers bureaus, manuscript writing or educational events	X	
6	Payment for expert testimony	X	
7	Support for attending meetings and/or travel	X	
8	Patents planned, issued or pending	X	
9	Participation on a Data Safety Monitoring Board or Advisory Board	X	
10	Leadership or fiduciary role in other board, society, committee or advocacy group, paid or unpaid	X	
11	Stock or stock options	X	
12	Receipt of equipment, materials, drugs, medical writing, gifts or other services	X	
13	Other financial or non-financial interests	X	

Please place an "X" next to the following statement to indicate your agreement:

X I certify that I have answered every question and have not altered the wording of any of the questions on this form.

ICMJE DISCLOSURE FORM

Date:____03rd June 2025
Your Name:_ Helen M. McGettrick_
Manuscript Title:___ PEPITEM regulates the synovial microenvironment during immune-mediated inflammatory arthritis to limit disease
Manuscript number (if known):________ ar-25-0320

In the interest of transparency, we ask you to disclose all relationships/activities/interests listed below that are related to the content of your manuscript. "Related" means any relation with for-profit or not-for-profit third parties whose interests may be affected by the content of the manuscript. Disclosure represents a commitment to transparency and does not necessarily indicate a bias. If you are in doubt about whether to list a relationship/activity/interest, it is preferable that you do so.

The following questions apply to the author's relationships/activities/interests as they relate to the current manuscript only.

The author's relationships/activities/interests should be defined broadly. For example, if your manuscript pertains to the epidemiology of hypertension, you should declare all relationships with manufacturers of antihypertensive medication, even if that medication is not mentioned in the manuscript.

In item #1 below, report all support for the work reported in this manuscript without time limit. For all other items, the time frame for disclosure is the past 36 months.

		**Name all entities with whom you have this relationship or indicate none (add rows as needed)**	**Specifications/Comments (e.g., if payments were made to you or to your institution)**
		**Time frame: Since the initial planning of the work**	
1	All support for the present manuscript (e.g., funding, provision of study materials, medical writing, article processing charges, etc.) **No time limit for this item.**	MRC	Grant paid to the University
		Pfizer	Grant paid to the University
		Research into Inflammatory Arthritis Centre Versus Arthritis (RACE)	Grant paid to the University
		NIHR Birmingham Biomedical Research Centres	Grant paid to the University
		Chernajovsky Foundation and Versus Arthritis Connect Immune	Grant paid to the University
		**Time frame: past 36 months**	
2	Grants or contracts from any entity (if not indicated in item #1 above).	MRC	Grant paid to the University
3	Royalties or licenses		
		X	
4	Consulting fees		

		X	
5	Payment or honoraria for lectures, presentations, speakers bureaus, manuscript writing or educational events		
		X	
6	Payment for expert testimony		
		X	
7	Support for attending meetings and/or travel		
		X	
8	Patents planned, issued or pending	Patents	Composition of matter patent for PEPITEM awarded to University of Birmingham
9	Participation on a Data Safety Monitoring Board or Advisory Board		
		X	
10	Leadership or fiduciary role in other board, society, committee or advocacy group, paid or unpaid		
		X	
11	Stock or stock options		
		X	
12	Receipt of equipment, materials, drugs, medical writing, gifts or other services		
		X	
13	Other financial or non-financial interests		
		X	

Please place an "X" next to the following statement to indicate your agreement:

X- I certify that I have answered every question and have not altered the wording of any of the questions on this form.
