# Supplementary material for: PEPITEM Regulates the Synovial Microenvironment During Immune‐Mediated Inflammatory Arthritis to Limit Disease
Source: Arthritis Rheumatol. 2026 Apr 13;78(7):1446–64. doi: 10.1002/art.70108 (PMC13313099; doi:10.1002/art.70108)
Supplement: Supplementary file 2 — Supplementary Figure 1: Gating strategy for identifying immune cell populations in murine models of arthritis. Leukocytes were identified using forward (FSC) and side scatter (SSC), prior to defining single and then live CD45+ leukocytes. Gating strategy to identify CD3+ T‐cells, Ly6G+CD11b+Ly6C+ neutrophils, CD11b+Ly6C+ monocytes and CD11b+F4/80+ macrophages. Supplementary Figure 2: Gating strategy for adiponectin receptor expression on human peripheral blood mononuclear cells. The flow cytometry gating strategy used to phenotype adiponectin receptor expression on human peripheral mononuclear cells. Gating strategy to defined PBMC based on FSC and SSC, prior to determine single cells and then live PBMC based on exclusion of a live/dead marker prior to identifying CD19+ positive B‐cells and CD14+ monocytes. The percentage of PBMC, B‐cells or monocytes positively staining for adiponectin receptor was determined by gating to exclude any cells falling within the isotype region. Overlay histograms show expression of adiponectin receptor 1 and 2 (ADIPOR1, ADIPOR2, respectively) for each patient group. Supplementary Figure 3: Expression of adiponectin receptor 1 on leukocytes from patients with RA and PsA. Peripheral blood mononuclear cells (PBMC) were isolated from patients with clinically suspect arthralgia (CSA, n=14), unclassified arthritis (UA, n=26), early RA (RA, n=47) or PsA (n=14) or age‐matched healthy controls (HC, n=12). Adiponectin receptor 1 (A) gene or (B‐D) protein expression was assessed on (A‐B) PBMC, (C) CD19+ B‐cells or (D) monocytes by (A) qPCR or (B‐D) flow cytometry. (A) Gene expression was expressed as 2−ΔCT relative to the housekeeping gene ‐ β2M. ANOVA shows a significant effect of patient group on adiponectin receptor 1 expression, p<0.05. Protein expression was expressed as the percentage of (B) PBMC, or (C) B‐cells or (D) monocytes positive for adiponectin receptor staining. (E) Plasma adiponectin concentrations expressed as μg/ml – n=10 per g [file ART-78-1446-s001.docx]

***
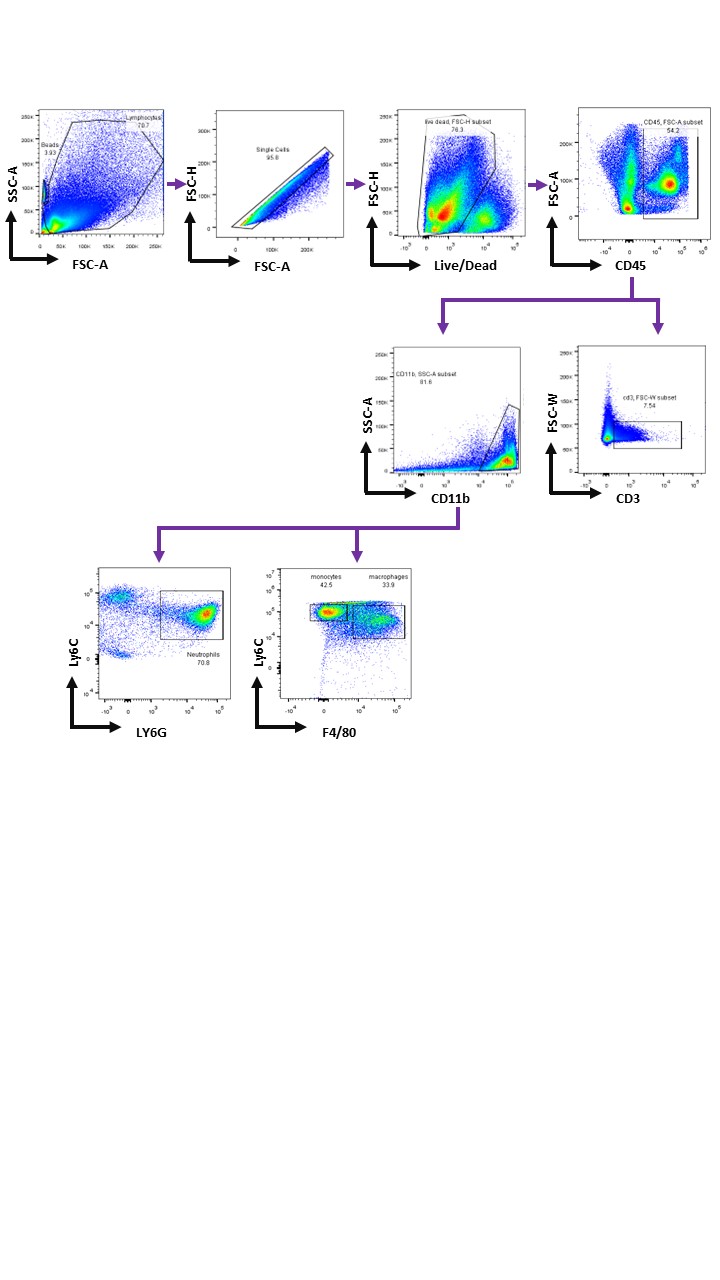
***

***Supplementary Figure 1 – Gating strategy for identifying immune cell populations in murine models of arthritis*.** Leukocytes were identified using forward (FSC) and side scatter (SSC), prior to defining single and then live CD45^+^ leukocytes. Gating strategy to identify CD3^+^ T-cells, Ly6G^+^CD11b^+^Ly6C^+^ neutrophils, CD11b^+^Ly6C^+^ monocytes and CD11b^+^F4/80^+^ macrophages.


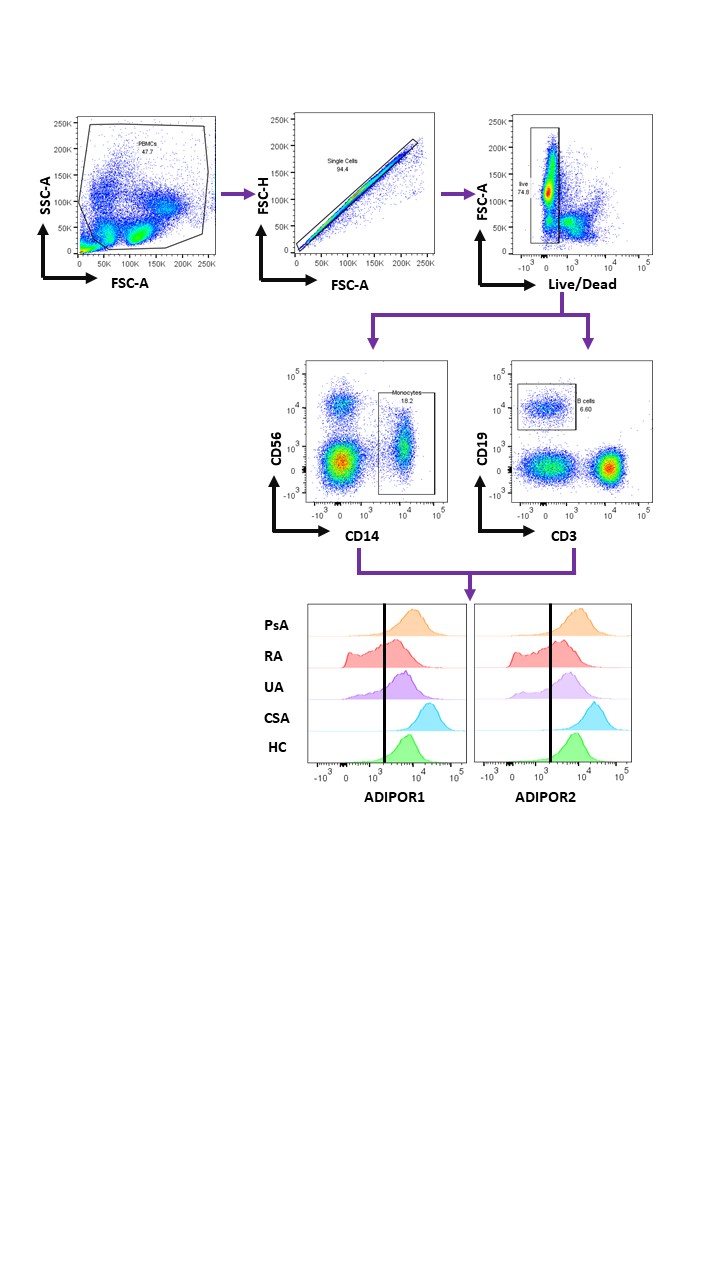


**Supplementary Figure 2 – Gating strategy for adiponectin receptor expression on human peripheral blood mononuclear cells.** The flow cytometry gating strategy used to phenotype adiponectin receptor expression on human peripheral mononuclear cells. Gating strategy to defined PBMC based on FSC and SSC, prior to determine single cells and then live PBMC based on exclusion of a live/dead marker prior to identifying CD19^+^ positive B-cells and CD14^+^ monocytes. The percentage of PBMC, B-cells or monocytes positively staining for adiponectin receptor was determined by gating to exclude any cells falling within the isotype region. Overlay histograms show expression of adiponectin receptor 1 and 2 (ADIPOR1, ADIPOR2, respectively) for each patient group.

***
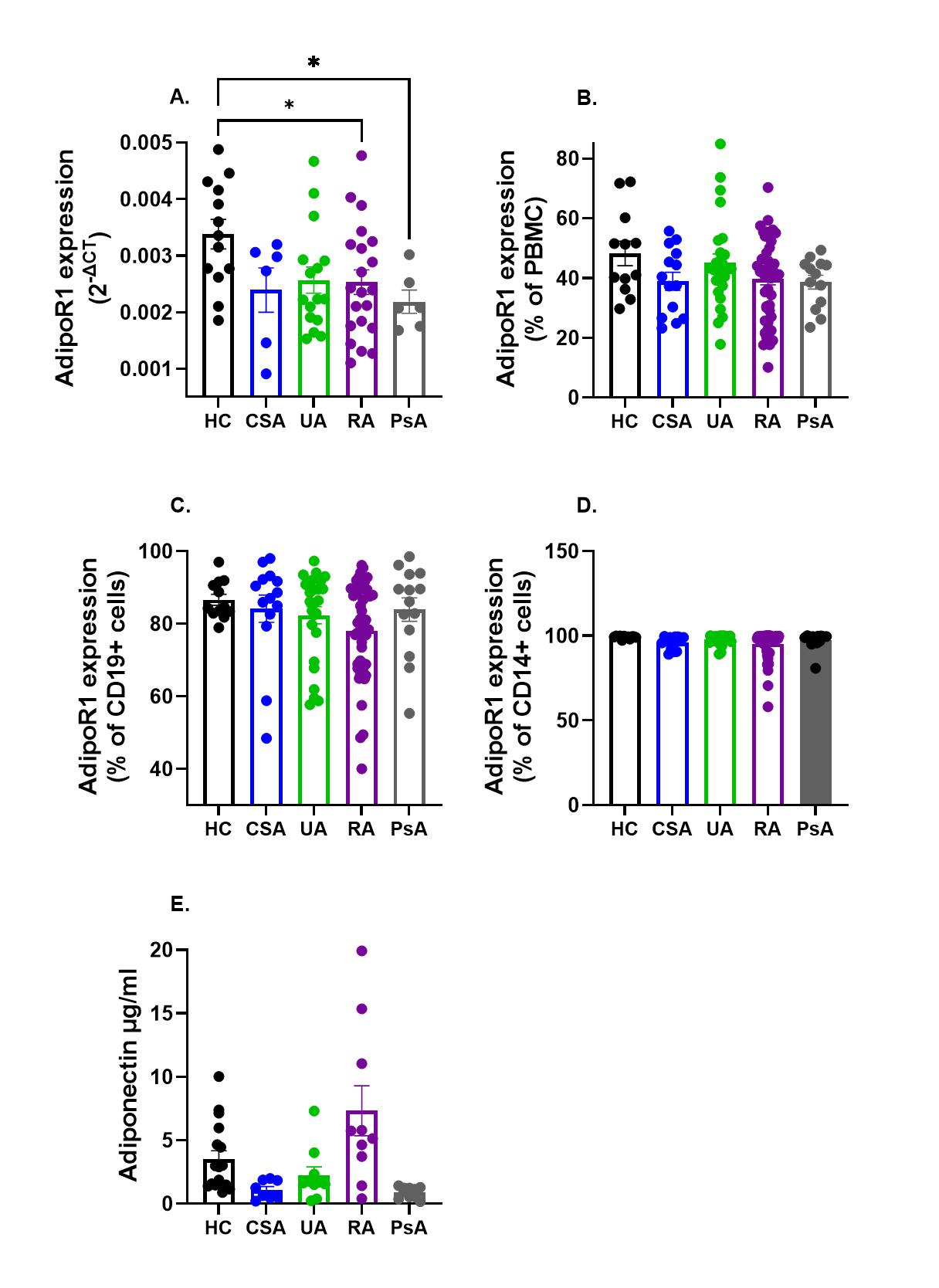
***

***Supplementary Figure 3: Expression of adiponectin receptor 1 on leukocytes from patients with RA and PsA.*** Peripheral blood mononuclear cells (PBMC) were isolated from patients with clinically suspect arthralgia (CSA, n=14), unclassified arthritis (UA, n=26), early RA (RA, n=47) or PsA (n=14) or age-matched healthy controls (HC, n=12). Adiponectin receptor 1 **(A)** gene or **(B-D)** protein expression was assessed on (A-B) PBMC, (C) CD19^+^ B-cells or (D) monocytes by (A) qPCR or (B-D) flow cytometry**. (A)** Gene expression was expressed as 2^-ΔCT^ relative to the housekeeping gene - β_2_M. ANOVA shows a significant effect of patient group on adiponectin receptor 1 expression, p<0.05. Protein expression was expressed as the percentage of **(B)** PBMC, or **(C)** B-cells or **(D)** monocytes positive for adiponectin receptor staining. **(E)** Plasma adiponectin concentrations expressed as µg/ml – n=10 per group, with n=17 for HC. Data are mean ± SEM. *= p<0.05 by Dunnett.

**
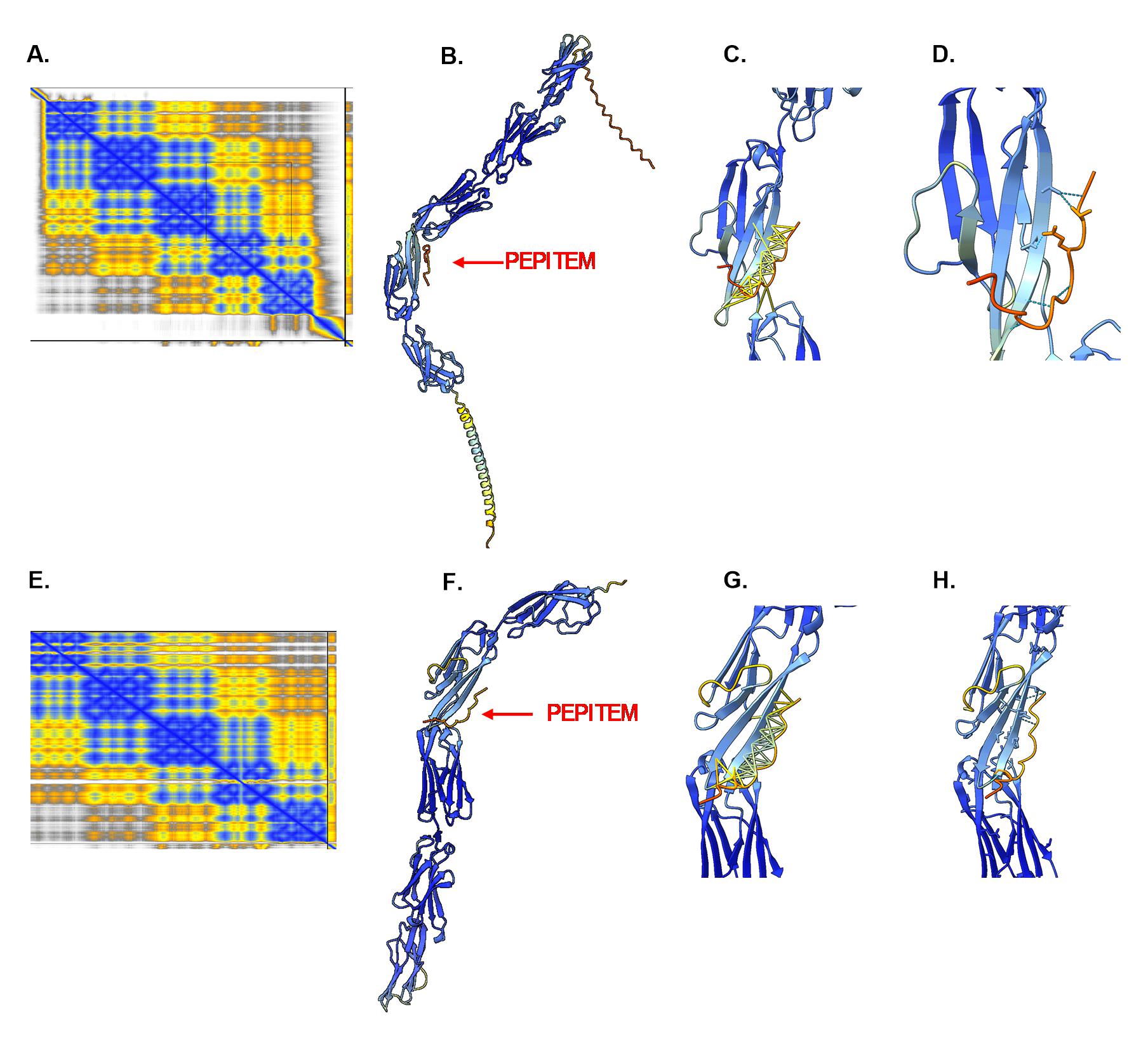
**

***Supplementary Figure 4: PEPITEM bioavailability is influenced by interactions with sICAM-1.* (A-D)** Full length ICAM-1 or **(E-H)** soluble ICAM-1 were ran with PEPITEM in (A, E) AlphaFold-Multimer followed by analysed of rank 1 model (B-D, F-H) using ChimeraX. Predicted aligned error heat map for one model of **(B)** ICAM-1 or **(F)** sICAM-1 domain and PEPITEM, where blue and yellow indicate low or high error, respectively. ChimeraX modelling of rank 1 model for PEPITEM interactions with **(C-D)** ICAM-1 or **(G-H)** sICAM-1 coloured by pLDDT. **(C-D, G-H)** Magnified view of binding location of PEPITEM on ICAM-1 revealing **(C)** 7 and **(G)** 6 predicted hydrogen bonds - high likelihood (blue), low (orange) - and **(D)** 32 and **(H)** 37 pseudobonds based on a distance of 5Å, confidence indicated by pLDDT colour as above.


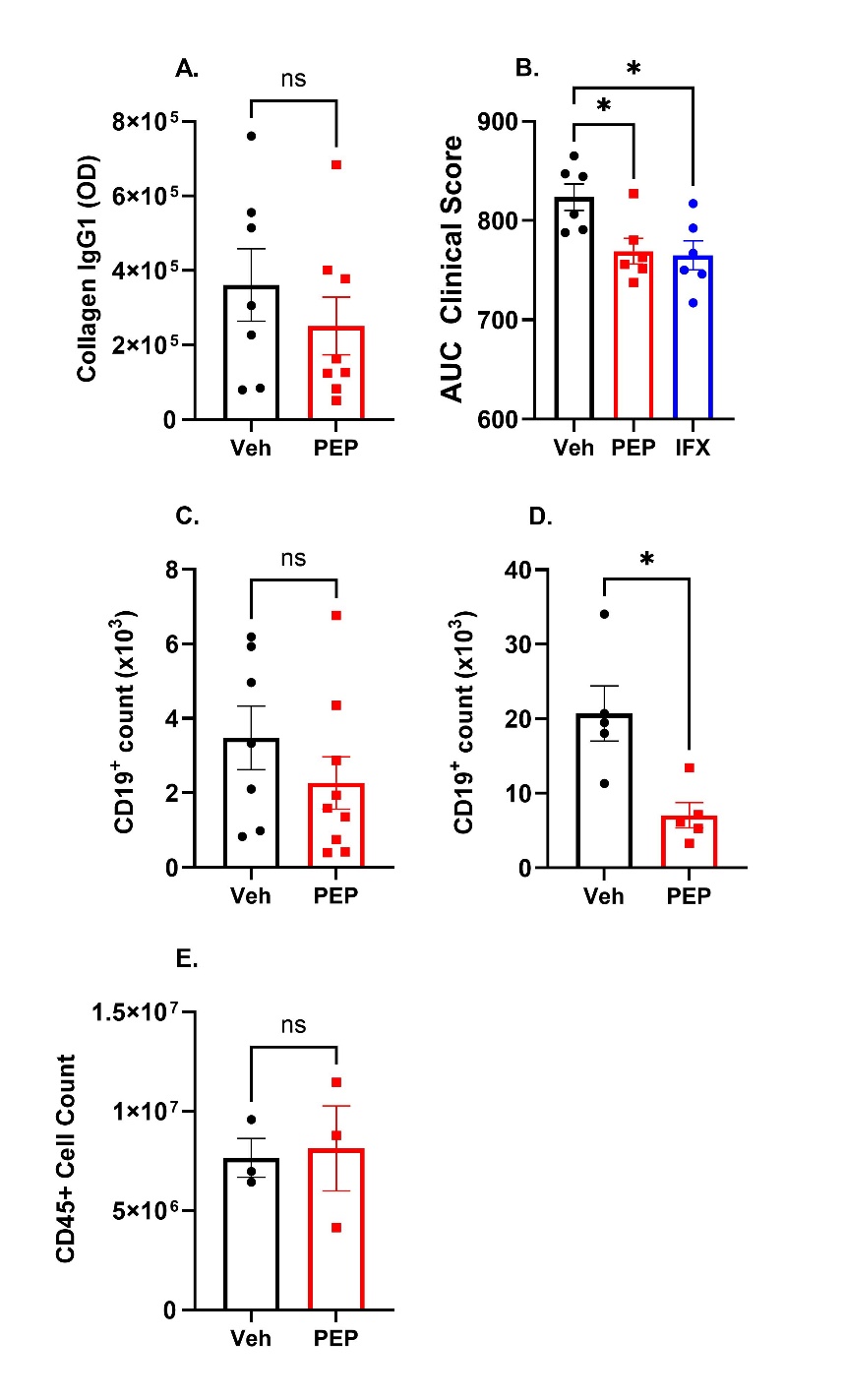


***Supplementary Figure 5: PEPITEM inhibitory activity is comparable to that of infliximab, but has no effect on lymph node responses.* (A, C, E)** In CIA, mice were treated with vehicle control (Veh, black) or PEPITEM-PEG (PEP, red) from day 21 onwards. **(A)** Autoantibody production, **(C)** CD19^+^ cells in the synovium and **(E)** CD45^+^ cells in the draining lymph node from CIA mice. **(B, D)** In the AIA model, mice were treated with vehicle control (Veh, black) or PEPITEM-PEG (PEP, red) or **(B)** infliximab (IFX, blue) from day 21 onwards. AIA-induced knee thickness (n=6) was expressed as AUC for the percentage change from baseline for each animal. In B, ANOVA shows a significant effect of treatment on joint thickness, p<0.05. **(D)** CD45^+^ cells in the draining lymph node from AIA mice. Data are mean ± SEM from at least n=2 independent experiment. *= p<0.05 by (B) Bonferroni post-test or (D) unpaired t-test compared to vehicle control.


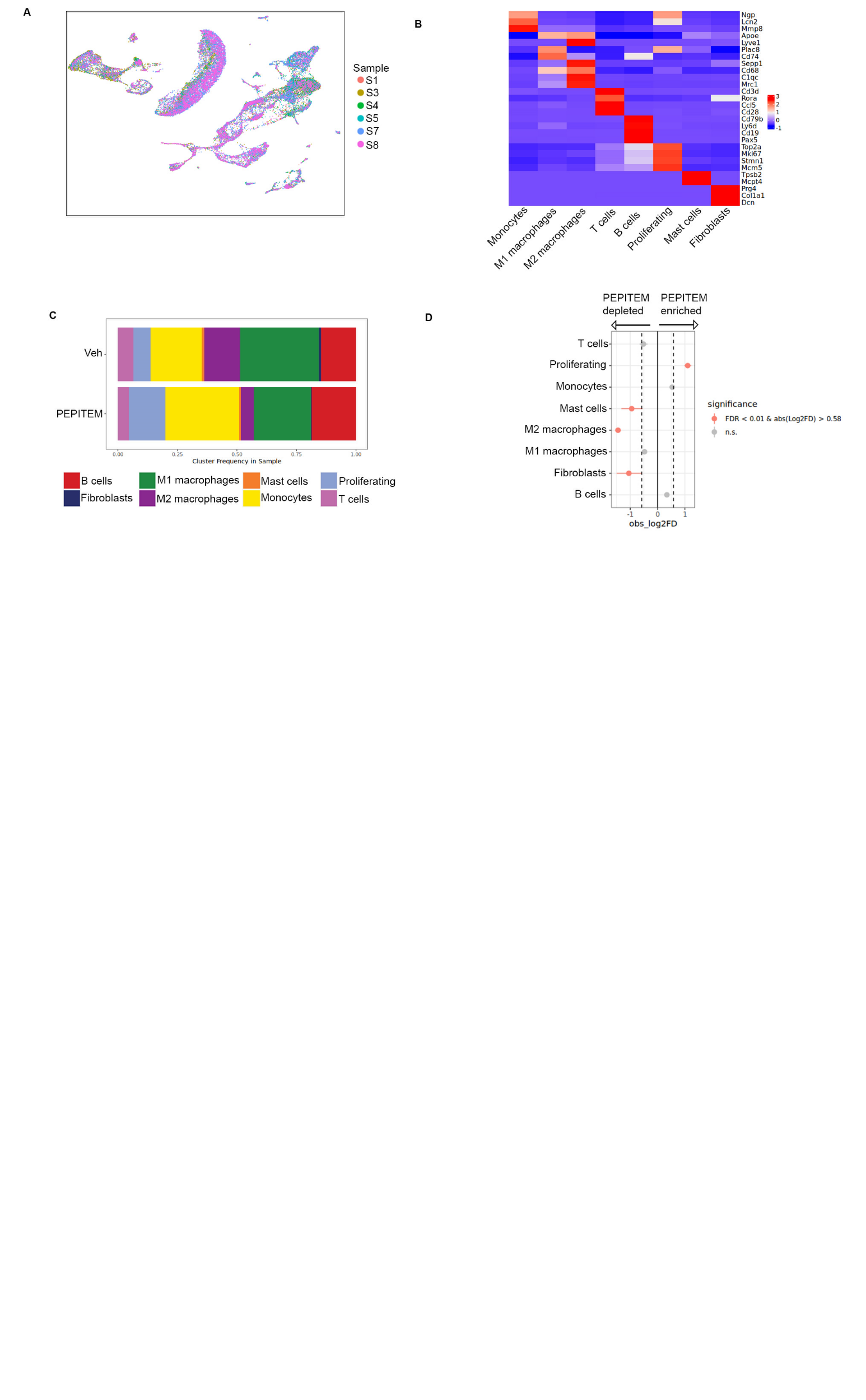


***Supplementary Figure 6: scRNAseq quality control and data extraction* (A)** UMAP of cells labelled by sample. **(B)** Heatmap of top genes per cluster. **(C-D)** Differential abundance analysis of cell types in vehicle or PEPITEM treated cells.


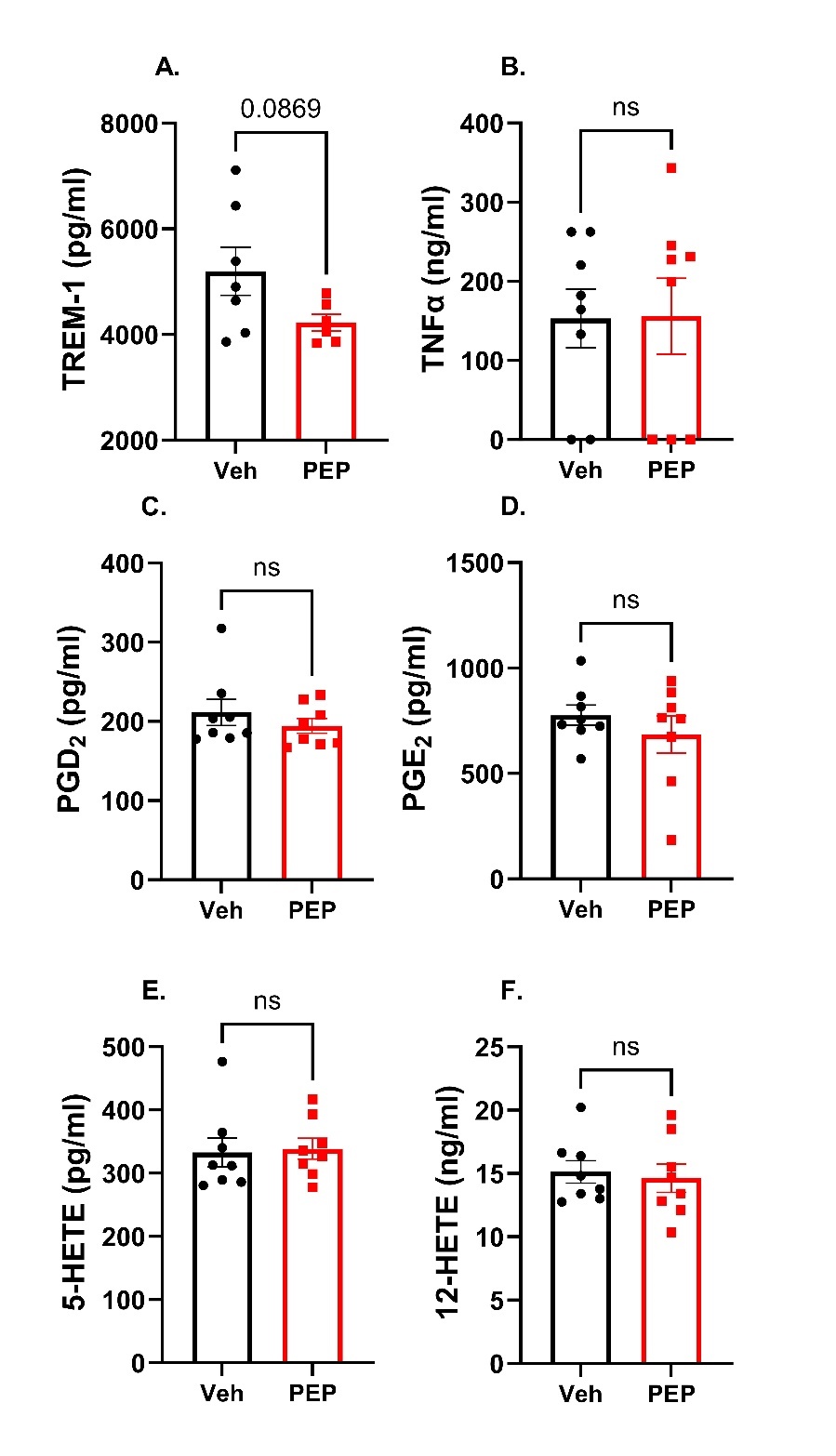


***Supplementary Figure 7 – PEPITEM had no effect on TNFα or prostanoid levels.*** Synovial tissue from AIA mice were treated with vehicle control (Veh, black) or PEPITEM (PEP, red) was collected at the peak of inflammation (day 3). After tissue digestion, the acellular fraction was analysed by ELISA for **(A)** TREM-1, (**B)** TNFα, **(C)** PGD_2_, **(D)** PGE_2_, **(E)** 5-HETE and **(F)** 12-HETE expressed as (B, F) ng/ml or (A, C-E) pg/ml n=7-8 mice per condition. Data are mean ± SEM from at least n=1 independent experiment, analysed using unpaired t-test.

***
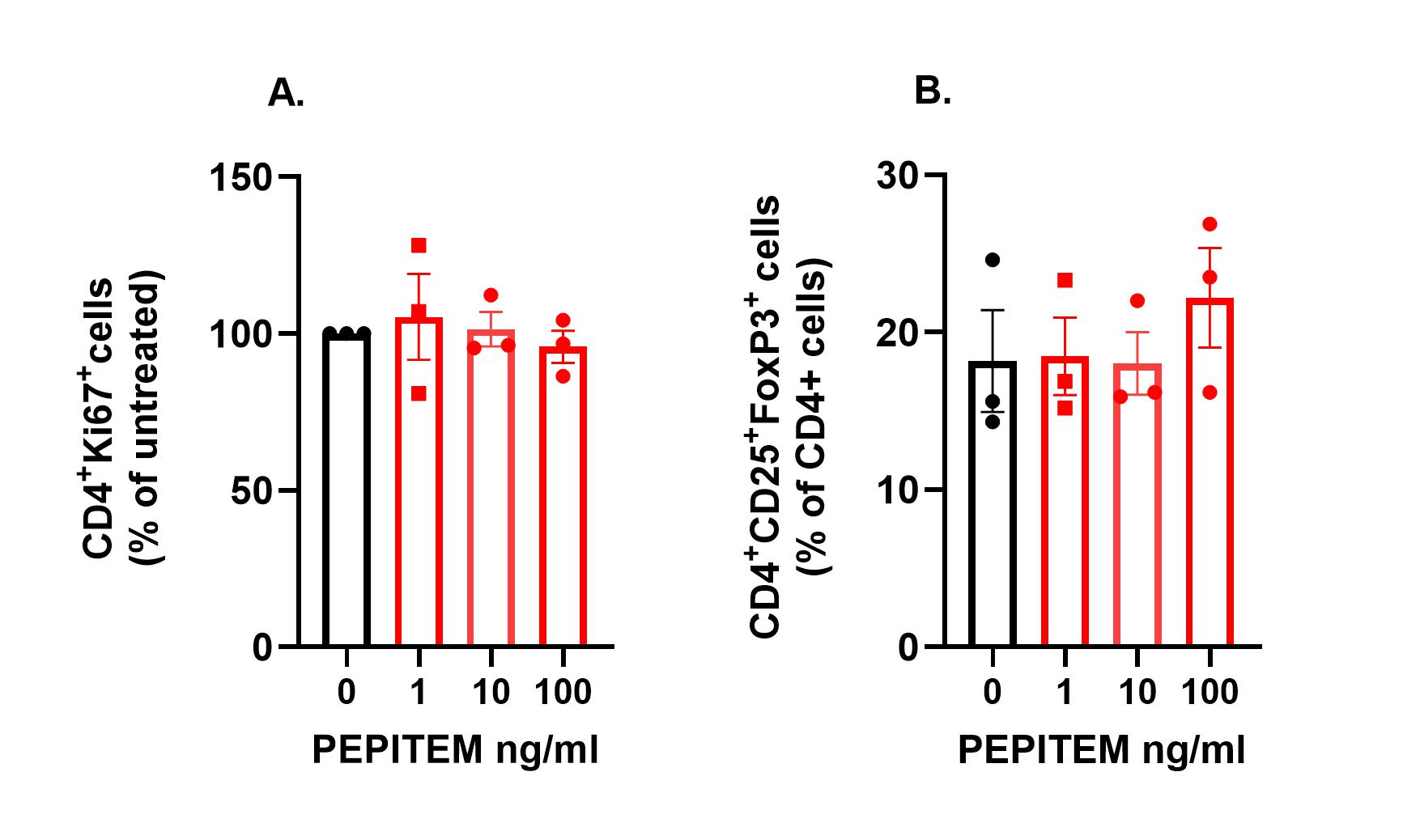
***

***Supplementary Figure 8 – PEPITEM had no effect on T-cell activation or polarisation.*** CD4+ native T-cells were polarised towards Treg or T_H1_ using a combination of antibody cocktails and differentiation media, treated in the presence of absence of PEPITEM. Gating strategy to defined T-cells based on FSC and SSC, prior to determine single cells and then live T-cells based on exclusion of a live/dead marker. Gating strategy used CD3^+^CD4^+^ to identify T-cells, **(B)** CD25+Foxp3+ to denote Tregs and **(A)** Ki67 was used to assess T-cell proliferation. Data are mean ± SEM from at least n=3 independent experiment.

***
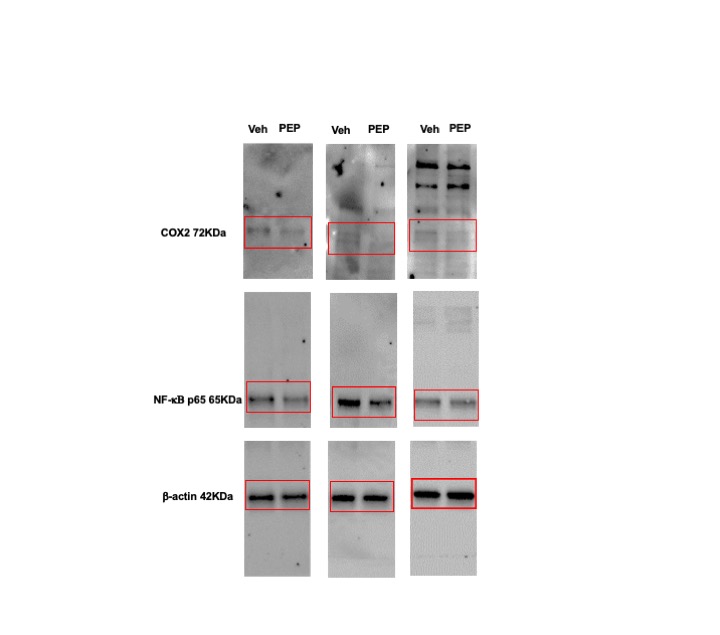
***

***Supplementary Figure 9 – Western blot gels for COX2, NF-κB and β-actin.*** COX-2, NF-κB p65 and β-actin protein in whole knee joint homogenates from vehicle or PEPITEM treated AIA mice was assessed at the peak of inflammation by western blot analysis. Images represent three separate independent experiments run each with n=4-8 mice per group pooled.


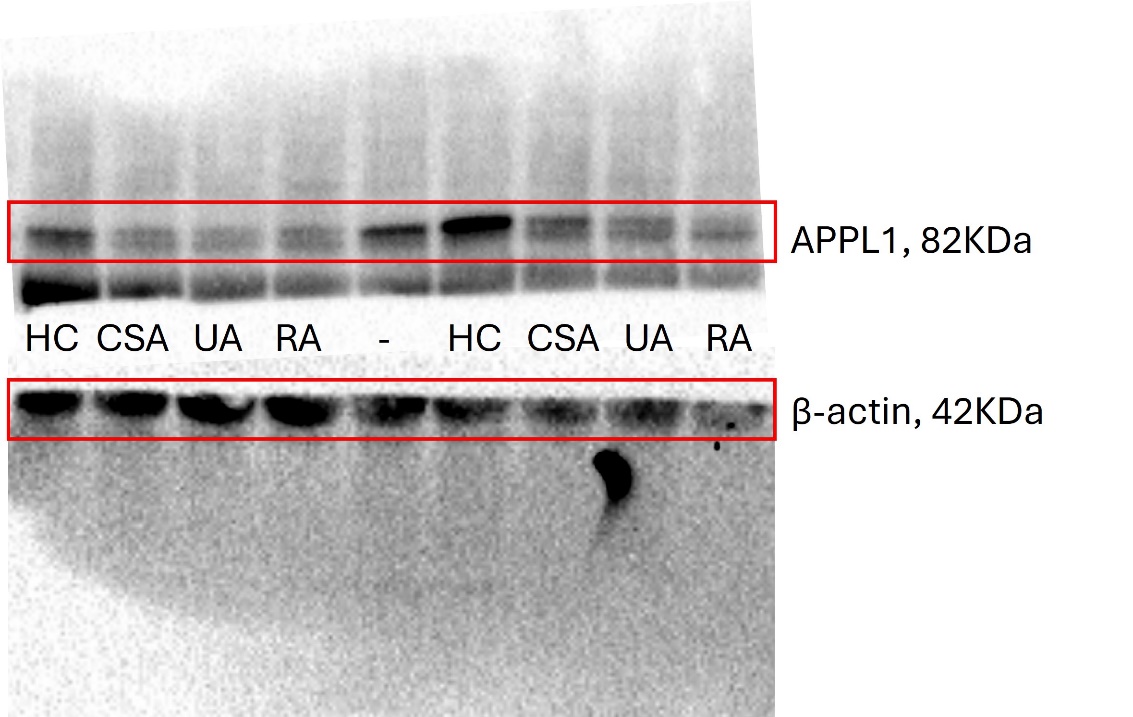


***Supplementary Figure 10 – Western blot gels for APPL-1, and β-actin.*** APPL-1 and β-actin protein in peripheral blood mononuclear cells (PBMC) from patients with clinically suspect arthralgia (CSA), unclassified arthritis (UA), early RA (RA) or age-matched healthy controls (HC) was assessed by western blot analysis. Images represent two separate independent experiments run each with n=2 patients per gel.
