# Supplementary material for: PEPITEM Regulates the Synovial Microenvironment During Immune‐Mediated Inflammatory Arthritis to Limit Disease
Source: Arthritis Rheumatol. 2026 Apr 13;78(7):1446–64. doi: 10.1002/art.70108 (PMC13313099; doi:10.1002/art.70108)
Supplement: Supplementary file 3 — Supplementary Table 1 Patient Clinical Characteristics Supplementary Table 2: DEG in monocytes from PEPITEM vs vehicle treated mice Supplementary Table 3: DEG in macrophages from PEPITEM vs vehicle treated mice [file ART-78-1446-s003.docx]

| **Supplementary Table 1 – Patient Clinical Characteristics** | | | | | |
| --- | --- | --- | --- | --- | --- |
|  | **HC**  **(n=13)** | **CSA**  **(n=15)** | **UA**  **(n=34)** | **RA**  **(n=59)** | **PsA**  **(n=34)** |
| **Age (years) ^†^** | 51 (35-70) | 49 (28-52) | 54 (45-65) | 59 (52-73) | 46 (30-61) |
| **Female; number (%)** | 8 (62) | 11 (74) | 21 (64) | 38 (66) | 6 (37.5) |
| **Symptom duration (weeks) ^†^** | **-** | 27 (18-75) | 20 (8-67) | 28 (14-52) | 34 (21-144) |
| **DAS28 CRP at baseline^††^** | **-** | 2.9 ± 0.3 | 3.5 ± 0.2 | 5.2 ± 0.2 | 3.8 ± 0.2 |
| **ESR (mm/h)^†^** | **-** | 16 (10-29) | 11 (5-35) | 30 (21-45) | 23 (8-40) |
| **CRP (mg/l)** **^†^** | **-** | 4 (3-9) | 8 (3-19) | 20 (7-34) | 8 (4-28) |
| **RF positive (%)** | **-** | 2 (13) | 7 (21) | 34 (59) | 0 (0) |
| **ACPA positive (%)** | **-** | 3 (20) | 4 (12) | 34 (60) | 0 (0) |
| **SJC28^†^** | **-** | 0 (0) | 1 (1-3) | 8 (2-13) | 2 (1-5) |
| **TJC28^†^** | **-** | 1 (0-5) | 2 (1-5) | 8 (5-11) | 2 (1-5) |
| **VAS^†^** | **-** | 47 (15-70) | 43 (24-68) | 67 (50-89) | 49 (28-66) |
| HC = healthy control, CSA = clinically suspect arthralgia, UA = unclassified arthritis  †median (interquartile range); ††mean±SD; RF = rheumatoid factor; ACPA = anti-citrullinated protein antibody; SJC28 = 28 swollen joint count; TJC28 = 28 tender joint count; VAS = global patient visual analogue scale; **-** = data not obtained from patients at time of presentation. | | | | | |

| **Supplementary Table 2 – DEG in monocytes from PEPITEM vs vehicle treated mice** |
| --- |

| **Gene Name** | **p_val** | **avg_log2FC** | **p_val_adj** |
| --- | --- | --- | --- |
| *Cdk8* | 1.67E-200 | -0.805643805 | 3.41E-196 |
| *Lars2* | 1.18E-152 | -0.70734091 | 2.41E-148 |
| *AY036118* | 1.86E-144 | -0.653205855 | 3.80E-140 |
| *Gphn* | 1.92E-123 | -0.586540473 | 3.91E-119 |
| *Il31ra* | 5.51E-111 | -0.394998126 | 1.12E-106 |
| *Camk1d* | 3.88E-94 | -0.459892149 | 7.91E-90 |
| *Gm26561* | 5.15E-79 | -0.483203311 | 1.05E-74 |
| *Lilr4b* | 5.44E-64 | -0.492466346 | 1.11E-59 |
| *Cebpb* | 1.66E-60 | -0.545989081 | 3.40E-56 |
| *Hexb* | 1.22E-50 | -0.371242451 | 2.49E-46 |
| *Cox17* | 2.90E-50 | -0.460388116 | 5.92E-46 |
| *Rps27* | 1.02E-48 | -0.356171985 | 2.07E-44 |
| *Eif1* | 1.06E-46 | -0.253186197 | 2.17E-42 |
| *Lilrb4a* | 7.77E-45 | -0.396305793 | 1.59E-40 |
| *Prdx5* | 5.63E-39 | 0.277467889 | 1.15E-34 |
| *Srgn* | 5.47E-37 | -0.36904146 | 1.12E-32 |
| *Ddx5* | 9.23E-36 | -0.291250261 | 1.88E-31 |
| *Jund* | 4.10E-34 | -0.421095684 | 8.37E-30 |
| *Slc15a3* | 8.91E-34 | -0.465429086 | 1.82E-29 |
| *Ccr1* | 1.13E-33 | -0.387745101 | 2.31E-29 |
| *Mcl1* | 3.65E-32 | -0.379959932 | 7.44E-28 |
| *Rpl27* | 1.89E-31 | -0.30829054 | 3.86E-27 |
| *Anp32a* | 1.72E-30 | -0.287097388 | 3.51E-26 |
| *Ckap4* | 8.54E-30 | 0.378878049 | 1.74E-25 |
| *Fosb* | 9.23E-30 | -0.440465388 | 1.88E-25 |
| *Chmp4b* | 6.46E-29 | -0.288895882 | 1.32E-24 |
| *Tkt* | 3.78E-28 | 0.323614557 | 7.71E-24 |
| *Ifitm6* | 1.69E-27 | 0.465433266 | 3.44E-23 |
| *Jdp2* | 1.87E-25 | -0.352879204 | 3.82E-21 |
| *Slc2a1* | 3.54E-25 | -0.374555591 | 7.22E-21 |
| *Malat1* | 3.56E-25 | -0.274245156 | 7.27E-21 |
| *Rps14* | 1.05E-24 | 0.272758734 | 2.14E-20 |
| *Cd14* | 2.08E-24 | -0.558573608 | 4.24E-20 |
| *Smox* | 3.91E-24 | -0.413588249 | 7.98E-20 |
| *C130026I21Rik* | 4.26E-24 | -0.311353736 | 8.69E-20 |
| *Selk* | 9.73E-24 | -0.263554895 | 1.98E-19 |
| *Fam101b* | 2.92E-23 | 0.310852303 | 5.95E-19 |
| *Fxyd5* | 8.40E-23 | -0.263586713 | 1.71E-18 |
| *Cnn2* | 1.00E-22 | 0.262241718 | 2.04E-18 |
| *Cybb* | 1.31E-22 | 0.438610742 | 2.68E-18 |
| *Rps24* | 2.01E-22 | 0.291415401 | 4.10E-18 |
| *Cd53* | 2.51E-22 | -0.265521857 | 5.13E-18 |
| *Ly6c2* | 3.25E-22 | 0.310941938 | 6.63E-18 |
| *Pnrc1* | 7.78E-22 | -0.32709619 | 1.59E-17 |
| *Tnfaip2* | 9.61E-22 | -0.592300945 | 1.96E-17 |
| *Il1r2* | 1.57E-21 | -0.386641121 | 3.20E-17 |
| *Stim2* | 1.58E-21 | 0.296139074 | 3.21E-17 |
| *Btg1* | 1.21E-20 | -0.276127012 | 2.47E-16 |
| *Plek* | 4.46E-20 | -0.434216728 | 9.11E-16 |
| *Sirpb1b* | 4.74E-20 | -0.319007125 | 9.67E-16 |
| *Ifitm1* | 5.84E-20 | -0.427332347 | 1.19E-15 |
| *Cd177* | 6.18E-20 | 0.330483514 | 1.26E-15 |
| *C5ar1* | 1.14E-19 | -0.264680149 | 2.33E-15 |
| *Adpgk* | 1.63E-19 | 0.329300139 | 3.33E-15 |
| *Egr1* | 2.56E-19 | -0.444012131 | 5.22E-15 |
| *Cd44* | 2.61E-19 | -0.322764574 | 5.33E-15 |
| *Ly6g* | 2.93E-19 | 0.344935807 | 5.98E-15 |
| *Cass4* | 9.88E-19 | -0.420846069 | 2.01E-14 |
| *Lamtor4* | 3.79E-18 | 0.269966796 | 7.72E-14 |
| *Nt5c2* | 6.24E-18 | 0.291568083 | 1.27E-13 |
| *Csrnp1* | 6.79E-18 | -0.302029349 | 1.38E-13 |
| *Hcar2* | 1.38E-17 | -0.528573315 | 2.81E-13 |
| *Trim30b* | 1.82E-17 | -0.251551484 | 3.72E-13 |
| *Mmp8* | 2.21E-17 | 0.328192473 | 4.51E-13 |
| *Clec4d* | 3.22E-17 | -0.279756786 | 6.57E-13 |
| *Emd* | 4.62E-17 | -0.275867397 | 9.42E-13 |
| *Zfp36l1* | 6.02E-17 | -0.417089613 | 1.23E-12 |
| *Pim1* | 8.23E-17 | -0.391785173 | 1.68E-12 |
| *Aldh2* | 9.13E-17 | 0.257954998 | 1.86E-12 |
| *Sdcbp* | 3.98E-16 | -0.297781255 | 8.12E-12 |
| *Il1b* | 4.58E-16 | -0.481499664 | 9.33E-12 |
| *Ranbp9* | 7.34E-16 | 0.292328247 | 1.50E-11 |
| *Junb* | 1.00E-15 | -0.384515713 | 2.04E-11 |
| *Pla2g7* | 1.20E-15 | -0.331834366 | 2.46E-11 |
| *Entpd1* | 1.24E-15 | -0.284471241 | 2.53E-11 |
| *Atrn* | 2.40E-15 | 0.279832432 | 4.90E-11 |
| *Scrg1* | 2.68E-15 | 0.361099207 | 5.46E-11 |
| *Stim1* | 4.40E-15 | 0.345076828 | 8.97E-11 |
| *Wfdc17* | 6.15E-15 | -0.435826831 | 1.25E-10 |
| *Dusp1* | 7.51E-15 | -0.385752136 | 1.53E-10 |
| *Ptafr* | 7.61E-15 | -0.313617268 | 1.55E-10 |
| *Fth1* | 7.70E-15 | -0.317094982 | 1.57E-10 |
| *Cxcl2* | 1.50E-14 | -0.684496022 | 3.06E-10 |
| *Camp* | 1.75E-14 | 0.28689391 | 3.58E-10 |
| *Dach1* | 1.83E-14 | 0.390231425 | 3.74E-10 |
| *Ptgs2* | 2.20E-14 | -0.435705194 | 4.48E-10 |
| *Socs3* | 7.75E-14 | -0.272677877 | 1.58E-09 |
| *Syne1* | 9.40E-14 | 0.277759367 | 1.92E-09 |
| *Tgm2* | 9.60E-14 | -0.318853311 | 1.96E-09 |
| *Trem1* | 3.42E-13 | -0.266327896 | 6.98E-09 |
| *Marcks* | 3.49E-13 | -0.295351699 | 7.12E-09 |
| *Plekho2* | 4.23E-13 | -0.260541434 | 8.63E-09 |
| *Lrrk2* | 4.48E-13 | 0.250949164 | 9.14E-09 |
| *Fgl2* | 5.82E-13 | -0.347086577 | 1.19E-08 |
| *Asprv1* | 6.81E-13 | 0.398020682 | 1.39E-08 |
| *Atf3* | 1.21E-12 | -0.449380706 | 2.47E-08 |
| *H2-Q10* | 1.60E-12 | -0.284690755 | 3.27E-08 |
| *Hmgn2* | 1.71E-12 | -0.280485781 | 3.48E-08 |
| *Ltf* | 1.82E-12 | 0.271293672 | 3.72E-08 |
| *Cxcr4* | 2.07E-12 | -0.325232158 | 4.21E-08 |
| *Tbc1d8* | 2.09E-12 | 0.307932621 | 4.26E-08 |
| *Trim30d* | 2.61E-12 | -0.253777901 | 5.32E-08 |
| *Snrk* | 2.95E-12 | 0.266143272 | 6.02E-08 |
| *Il1rap* | 3.42E-12 | -0.387803827 | 6.97E-08 |
| *Rassf3* | 4.11E-12 | 0.261603184 | 8.38E-08 |
| *Cstb* | 4.92E-12 | -0.307801586 | 1.00E-07 |
| *Vps37b* | 6.74E-12 | -0.263415052 | 1.37E-07 |
| *Clec4n* | 6.94E-12 | -0.444108443 | 1.42E-07 |
| *Alcam* | 7.81E-12 | -0.261402672 | 1.59E-07 |
| *Bcl2a1b* | 9.92E-12 | -0.433651165 | 2.02E-07 |
| *Tnfaip8* | 1.21E-11 | 0.259584752 | 2.47E-07 |
| *Ier3* | 1.60E-11 | -0.321499852 | 3.27E-07 |
| *Ccl3* | 4.19E-11 | -0.609844723 | 8.54E-07 |
| *Il1f9* | 8.16E-11 | -0.280701273 | 1.67E-06 |
| *Stxbp5* | 9.76E-11 | 0.267626416 | 1.99E-06 |
| *Golim4* | 1.04E-10 | 0.312921237 | 2.12E-06 |
| *Tiparp* | 1.53E-10 | -0.26139256 | 3.12E-06 |
| *Ntng2* | 1.55E-10 | 0.285194962 | 3.16E-06 |
| *Il1rn* | 5.07E-10 | -0.359655061 | 1.03E-05 |
| *Neat1* | 7.14E-10 | -0.277854541 | 1.46E-05 |
| *Pde7b* | 8.08E-10 | -0.302885107 | 1.65E-05 |
| *Ppp1r15a* | 8.14E-10 | -0.282060775 | 1.66E-05 |
| *Chil3* | 1.03E-09 | 0.357987941 | 2.10E-05 |
| *Rps18* | 1.15E-09 | 0.266203693 | 2.34E-05 |
| *Epb41* | 1.96E-09 | 0.307480623 | 4.00E-05 |
| *Mbnl1* | 1.52E-08 | 0.328121928 | 0.000309848 |
| *G0s2* | 1.78E-08 | -0.300365295 | 0.000362164 |
| *Thbs1* | 3.14E-08 | -0.277774448 | 0.000639362 |
| *Trim30a* | 1.29E-07 | -0.300159191 | 0.002640812 |
| *Irg1* | 1.41E-07 | -0.372018999 | 0.002883046 |
| *Csf1* | 2.36E-07 | -0.382502791 | 0.004822774 |
| *Ccl4* | 2.84E-07 | -0.713071713 | 0.005791062 |
| *Trf* | 2.88E-07 | -0.263429249 | 0.005864262 |
| *Nfe2l2* | 7.97E-07 | -0.268017451 | 0.016255638 |
| *Slfn4* | 8.24E-07 | -0.410105284 | 0.016796897 |
| *Nr4a1* | 9.13E-07 | -0.267660884 | 0.018627882 |
| *Basp1* | 1.99E-06 | -0.331002478 | 0.040554425 |

| **Supplementary Table 3 – DEG in macrophages from PEPITEM vs vehicle treated mice** | | | | |
| --- | --- | --- | --- | --- |
| **Gene name** | **p_val** | **avg_log2FC** | **p_val_adj** |  |
| *AY036118* | 1.01E-228 | -0.644510559 | 2.05E-224 |  |
| *Il31ra* | 1.01E-179 | -0.377770284 | 2.07E-175 |  |
| *Cdk8* | 1.94E-135 | -0.464013109 | 3.95E-131 |  |
| *Uba52* | 1.31E-112 | -0.415497661 | 2.67E-108 |  |
| *Lars2* | 6.27E-103 | -0.442840021 | 1.28E-98 |  |
| *Ubb* | 9.56E-102 | -0.36468584 | 1.95E-97 |  |
| *Coro1a* | 4.30E-98 | 0.414784369 | 8.77E-94 |  |
| *Arhgap15* | 9.02E-97 | 0.472862071 | 1.84E-92 |  |
| *Malat1* | 1.90E-93 | -0.470454955 | 3.87E-89 |  |
| *Rps9* | 2.28E-91 | 0.307066776 | 4.64E-87 |  |
| *Rpl15* | 1.53E-90 | -0.389898528 | 3.13E-86 |  |
| *Jund* | 1.64E-90 | -0.432953886 | 3.34E-86 |  |
| *Ubc* | 3.09E-88 | -0.406051616 | 6.29E-84 |  |
| *C1qb* | 5.80E-86 | -0.713493872 | 1.18E-81 |  |
| *C1qc* | 1.27E-85 | -0.731241838 | 2.59E-81 |  |
| *C1qa* | 4.58E-80 | -0.703574164 | 9.33E-76 |  |
| *Rgl1* | 3.29E-76 | -0.468970868 | 6.72E-72 |  |
| *Dok2* | 1.46E-75 | -0.279062914 | 2.97E-71 |  |
| *Rbpj* | 1.57E-75 | -0.443865798 | 3.20E-71 |  |
| *Gm10073* | 2.04E-75 | 0.366222512 | 4.16E-71 |  |
| *Rasgef1b* | 2.28E-75 | -0.514592697 | 4.65E-71 |  |
| *Ramp1* | 2.40E-75 | 0.33472711 | 4.90E-71 |  |
| *Ms4a6d* | 3.27E-74 | -0.457353611 | 6.68E-70 |  |
| *Dgkg* | 5.90E-74 | 0.433314245 | 1.20E-69 |  |
| *Tyrobp* | 8.23E-71 | 0.263754623 | 1.68E-66 |  |
| *Arhgdib* | 1.26E-70 | 0.338378444 | 2.56E-66 |  |
| *Mrc1* | 1.59E-70 | -0.713068031 | 3.24E-66 |  |
| *Rab3il1* | 1.97E-70 | -0.282157106 | 4.02E-66 |  |
| *Cybb* | 5.22E-70 | 0.39836739 | 1.06E-65 |  |
| *Sell* | 8.95E-70 | 0.400645252 | 1.82E-65 |  |
| *Prdx5* | 9.95E-70 | 0.443583884 | 2.03E-65 |  |
| *Socs3* | 4.07E-69 | -0.458662107 | 8.29E-65 |  |
| *Sdcbp* | 4.51E-69 | -0.305756694 | 9.20E-65 |  |
| *Dab2* | 1.57E-68 | -0.528912355 | 3.20E-64 |  |
| *Fcgr2b* | 2.97E-68 | -0.407407665 | 6.05E-64 |  |
| *Mgl2* | 3.13E-68 | -0.564558193 | 6.38E-64 |  |
| *Hspa5* | 3.23E-68 | -0.424009762 | 6.59E-64 |  |
| *St13* | 3.71E-68 | -0.276088134 | 7.56E-64 |  |
| *Pmp22* | 9.26E-68 | -0.512410994 | 1.89E-63 |  |
| *Slamf7* | 8.28E-67 | -0.352775005 | 1.69E-62 |  |
| *Pltp* | 4.06E-66 | -0.598652673 | 8.27E-62 |  |
| *Rps14* | 3.14E-65 | 0.256397158 | 6.39E-61 |  |
| *Rpl35* | 2.36E-64 | -0.297140207 | 4.81E-60 |  |
| *Ifitm6* | 1.45E-63 | 0.639856356 | 2.96E-59 |  |
| *B2m* | 3.47E-63 | -0.370967456 | 7.08E-59 |  |
| *C3ar1* | 6.31E-63 | -0.35884458 | 1.29E-58 |  |
| *Folr2* | 8.52E-63 | -0.585046033 | 1.74E-58 |  |
| *Nedd9* | 3.27E-62 | 0.456235577 | 6.67E-58 |  |
| *Ccl7* | 8.91E-62 | -0.905723911 | 1.82E-57 |  |
| *Emp1* | 1.07E-61 | -0.50027672 | 2.17E-57 |  |
| *Maf* | 4.47E-61 | -0.506242766 | 9.12E-57 |  |
| *Tmem106a* | 2.54E-60 | -0.265718537 | 5.18E-56 |  |
| *Gmfg* | 7.85E-60 | 0.283824983 | 1.60E-55 |  |
| *Rac2* | 1.21E-59 | 0.320300441 | 2.46E-55 |  |
| *Mgst1* | 2.15E-59 | 0.465400903 | 4.39E-55 |  |
| *Atf3* | 3.88E-59 | -0.551076133 | 7.92E-55 |  |
| *Tuba1b* | 4.59E-59 | -0.255989094 | 9.37E-55 |  |
| *Taldo1* | 8.61E-59 | 0.270203231 | 1.76E-54 |  |
| *Fry* | 1.09E-58 | 0.343723782 | 2.22E-54 |  |
| *Frmd4b* | 1.22E-58 | -0.4102712 | 2.48E-54 |  |
| *Rpl27* | 1.77E-58 | -0.265175359 | 3.61E-54 |  |
| *Sdc3* | 1.81E-58 | -0.326771291 | 3.69E-54 |  |
| *F5* | 3.90E-58 | 0.458005191 | 7.95E-54 |  |
| *Lyst* | 3.97E-58 | 0.339238927 | 8.10E-54 |  |
| *Hp* | 6.40E-58 | 0.438451804 | 1.31E-53 |  |
| *Lmna* | 7.88E-58 | -0.361736637 | 1.61E-53 |  |
| *Lgmn* | 1.45E-57 | -0.394322149 | 2.95E-53 |  |
| *Rap1gap2* | 2.04E-57 | 0.322402542 | 4.16E-53 |  |
| *Fxyd2* | 3.58E-57 | -0.609634066 | 7.30E-53 |  |
| *Ighm* | 1.34E-56 | 0.596508033 | 2.74E-52 |  |
| *Ifi205* | 2.02E-56 | -0.411733123 | 4.12E-52 |  |
| *Oas1a* | 2.84E-56 | -0.255401684 | 5.79E-52 |  |
| *Gas6* | 3.90E-56 | -0.52093738 | 7.95E-52 |  |
| *Rps16* | 4.11E-56 | 0.254161975 | 8.39E-52 |  |
| *AI607873* | 4.13E-55 | -0.358967764 | 8.42E-51 |  |
| *Sorl1* | 7.79E-55 | 0.292102618 | 1.59E-50 |  |
| *Anxa4* | 4.36E-54 | -0.28410031 | 8.89E-50 |  |
| *Vim* | 9.08E-54 | -0.310687743 | 1.85E-49 |  |
| *Pgd* | 1.19E-53 | 0.284892181 | 2.43E-49 |  |
| *Mnda* | 3.11E-53 | -0.429495966 | 6.35E-49 |  |
| *Cytip* | 4.39E-53 | 0.348667237 | 8.96E-49 |  |
| *Gphn* | 4.61E-53 | -0.282947407 | 9.39E-49 |  |
| *Lamtor4* | 6.71E-53 | 0.269197369 | 1.37E-48 |  |
| *Klf6* | 4.24E-52 | -0.422036 | 8.64E-48 |  |
| *Lilr4b* | 1.26E-51 | -0.331696678 | 2.56E-47 |  |
| *Oasl1* | 1.27E-51 | -0.293504106 | 2.59E-47 |  |
| *Gsr* | 1.42E-51 | 0.31503507 | 2.89E-47 |  |
| *Tkt* | 1.82E-51 | 0.279108739 | 3.71E-47 |  |
| *Rpsa* | 2.87E-51 | 0.308027608 | 5.86E-47 |  |
| *Ifi204* | 7.00E-51 | -0.440741701 | 1.43E-46 |  |
| *Msrb1* | 7.62E-51 | 0.374823449 | 1.55E-46 |  |
| *Klhl2* | 8.61E-51 | 0.305853973 | 1.76E-46 |  |
| *Fn1* | 1.14E-50 | 0.596126036 | 2.32E-46 |  |
| *Gfod1* | 1.24E-50 | 0.414790219 | 2.52E-46 |  |
| *Cbr2* | 2.60E-50 | -0.481956593 | 5.30E-46 |  |
| *Cmip* | 3.09E-50 | 0.278430742 | 6.30E-46 |  |
| *Rpl18a* | 8.25E-50 | 0.289381158 | 1.68E-45 |  |
| *Fosb* | 1.09E-49 | -0.434785143 | 2.23E-45 |  |
| *Cd63* | 2.85E-49 | -0.413691674 | 5.82E-45 |  |
| *Cxcl16* | 3.44E-49 | -0.335781093 | 7.02E-45 |  |
| *Creb5* | 5.58E-49 | -0.330133069 | 1.14E-44 |  |
| *Limd2* | 1.66E-48 | 0.273715417 | 3.39E-44 |  |
| *Igf1* | 1.85E-48 | -0.263153587 | 3.78E-44 |  |
| *Ankrd44* | 4.06E-48 | 0.28908041 | 8.28E-44 |  |
| *Ccl12* | 5.81E-48 | -0.565604657 | 1.19E-43 |  |
| *Rnase6* | 6.39E-48 | 0.301279532 | 1.30E-43 |  |
| *Sbf2* | 1.28E-47 | -0.271735612 | 2.61E-43 |  |
| *Snx2* | 1.64E-47 | -0.295780308 | 3.34E-43 |  |
| *Fgfr1* | 2.57E-47 | -0.260334111 | 5.25E-43 |  |
| *Ctss* | 3.33E-47 | -0.270384748 | 6.78E-43 |  |
| *Egr2* | 8.08E-47 | -0.257968616 | 1.65E-42 |  |
| *Cd163* | 9.01E-47 | -0.592664905 | 1.84E-42 |  |
| *Gm26561* | 9.15E-47 | -0.285656677 | 1.87E-42 |  |
| *Xylt1* | 1.00E-46 | 0.380999077 | 2.04E-42 |  |
| *Mertk* | 2.54E-46 | -0.278367495 | 5.18E-42 |  |
| *Casp4* | 2.66E-46 | -0.322660744 | 5.43E-42 |  |
| *Ccl2* | 4.68E-46 | -0.733709961 | 9.54E-42 |  |
| *Plcb1* | 5.59E-46 | 0.447139068 | 1.14E-41 |  |
| *Chil3* | 1.16E-45 | 0.609908552 | 2.37E-41 |  |
| *Spred1* | 1.58E-45 | -0.300748496 | 3.22E-41 |  |
| *Cd68* | 3.36E-45 | -0.312380398 | 6.86E-41 |  |
| *Svil* | 6.71E-45 | 0.311257117 | 1.37E-40 |  |
| *Ctsb* | 9.11E-45 | -0.389798124 | 1.86E-40 |  |
| *Rps5* | 1.39E-44 | 0.268213932 | 2.83E-40 |  |
| *Mafb* | 2.35E-44 | -0.453493009 | 4.79E-40 |  |
| *Dusp1* | 3.81E-44 | -0.449913121 | 7.77E-40 |  |
| *Ecm1* | 1.37E-43 | -0.325330605 | 2.79E-39 |  |
| *Tmem176b* | 1.92E-43 | -0.345593556 | 3.91E-39 |  |
| *Stab1* | 2.76E-43 | -0.381684931 | 5.64E-39 |  |
| *Marcks* | 3.87E-43 | -0.283433392 | 7.89E-39 |  |
| *Junb* | 4.13E-43 | -0.28691277 | 8.42E-39 |  |
| *Plekhg5* | 1.23E-42 | -0.25907596 | 2.50E-38 |  |
| *Lyve1* | 2.16E-42 | -0.583601044 | 4.41E-38 |  |
| *Lyn* | 2.53E-42 | 0.258378897 | 5.16E-38 |  |
| *Metrnl* | 4.31E-42 | -0.285674787 | 8.79E-38 |  |
| *Fgr* | 5.90E-42 | 0.281420242 | 1.20E-37 |  |
| *Hmgb2* | 7.33E-42 | 0.496891383 | 1.49E-37 |  |
| *Rpl10a* | 1.08E-41 | 0.26490318 | 2.21E-37 |  |
| *Snx18* | 1.40E-41 | 0.278884192 | 2.85E-37 |  |
| *Runx2* | 2.56E-41 | 0.595333237 | 5.22E-37 |  |
| *Rps20* | 3.56E-41 | 0.253274422 | 7.26E-37 |  |
| *Pygl* | 6.17E-41 | 0.284973682 | 1.26E-36 |  |
| *Mmp8* | 1.05E-40 | 0.467614577 | 2.15E-36 |  |
| *Cd81* | 1.40E-40 | -0.38247495 | 2.86E-36 |  |
| *Samsn1* | 2.13E-40 | 0.292404225 | 4.34E-36 |  |
| *Pdia3* | 2.39E-40 | -0.257994335 | 4.87E-36 |  |
| *Stk10* | 3.55E-40 | 0.303624262 | 7.25E-36 |  |
| *Napsa* | 4.95E-40 | 0.273286806 | 1.01E-35 |  |
| *Sntb1* | 5.01E-40 | 0.25449672 | 1.02E-35 |  |
| *Pepd* | 7.28E-40 | -0.27842597 | 1.48E-35 |  |
| *Fam107b* | 8.42E-40 | 0.263119875 | 1.72E-35 |  |
| *Cd36* | 1.34E-39 | -0.442010999 | 2.73E-35 |  |
| *Atrnl1* | 1.66E-39 | 0.300252658 | 3.39E-35 |  |
| *Klf4* | 2.93E-39 | -0.373246246 | 5.97E-35 |  |
| *Trem3* | 3.26E-39 | 0.279888045 | 6.64E-35 |  |
| *Arhgap24* | 1.12E-38 | 0.298984062 | 2.28E-34 |  |
| *Myo1f* | 1.22E-38 | 0.286204804 | 2.50E-34 |  |
| *Rgs1* | 2.10E-38 | -0.606944217 | 4.29E-34 |  |
| *Pros1* | 5.86E-38 | -0.352563243 | 1.19E-33 |  |
| *Ssh2* | 9.54E-38 | 0.29310156 | 1.95E-33 |  |
| *Mcemp1* | 1.15E-37 | 0.293294331 | 2.34E-33 |  |
| *Pf4* | 1.45E-37 | -0.559998613 | 2.97E-33 |  |
| *Mtmr10* | 2.97E-37 | -0.302148236 | 6.06E-33 |  |
| *Lgals3bp* | 3.07E-37 | -0.296178344 | 6.27E-33 |  |
| *Irf7* | 8.54E-37 | -0.514256569 | 1.74E-32 |  |
| *Fcgrt* | 1.05E-36 | -0.397938207 | 2.13E-32 |  |
| *Gpr141* | 1.25E-36 | 0.330380875 | 2.54E-32 |  |
| *Ldlrad3* | 1.41E-36 | 0.338821757 | 2.88E-32 |  |
| *Itgb7* | 2.05E-36 | 0.258034207 | 4.18E-32 |  |
| *C4b* | 4.81E-36 | -0.386047173 | 9.80E-32 |  |
| *Ppp2r5a* | 5.05E-36 | 0.273080758 | 1.03E-31 |  |
| *Ms4a7* | 1.03E-35 | -0.416138917 | 2.10E-31 |  |
| *Mbnl1* | 1.33E-35 | 0.324959069 | 2.72E-31 |  |
| *Zfp36l1* | 2.93E-35 | -0.318670101 | 5.98E-31 |  |
| *Hspa8* | 6.11E-35 | -0.274211685 | 1.25E-30 |  |
| *Mt1* | 8.16E-35 | -0.626334549 | 1.66E-30 |  |
| *Ptafr* | 8.55E-35 | -0.272124187 | 1.74E-30 |  |
| *Mir142hg* | 1.03E-34 | 0.254921349 | 2.09E-30 |  |
| *Mpeg1* | 1.25E-34 | 0.256588881 | 2.54E-30 |  |
| *Cd177* | 1.59E-34 | 0.317298992 | 3.25E-30 |  |
| *Rab27a* | 3.21E-34 | 0.25543793 | 6.55E-30 |  |
| *Isg15* | 3.89E-34 | -0.762837274 | 7.93E-30 |  |
| *Fam65b* | 4.91E-34 | 0.283936186 | 1.00E-29 |  |
| *Atrn* | 5.54E-34 | 0.326772352 | 1.13E-29 |  |
| *Psd3* | 6.60E-34 | -0.332356434 | 1.35E-29 |  |
| *Ltc4s* | 6.63E-34 | -0.317160691 | 1.35E-29 |  |
| *Aqp1* | 8.85E-34 | -0.316489639 | 1.80E-29 |  |
| *Cers6* | 9.91E-34 | 0.286686251 | 2.02E-29 |  |
| *Tns1* | 1.94E-33 | -0.291555744 | 3.95E-29 |  |
| *Iqgap2* | 2.15E-33 | 0.299413236 | 4.38E-29 |  |
| *Mt2* | 3.93E-33 | -0.582497675 | 8.01E-29 |  |
| *Rtp4* | 6.04E-33 | -0.266292728 | 1.23E-28 |  |
| *Tbc1d8* | 6.49E-33 | 0.35003887 | 1.32E-28 |  |
| *Gda* | 6.84E-33 | 0.403302485 | 1.40E-28 |  |
| *Prkcb* | 9.02E-33 | 0.265564745 | 1.84E-28 |  |
| *Ccl8* | 6.68E-32 | -0.73733888 | 1.36E-27 |  |
| *Timp2* | 6.96E-32 | -0.492718598 | 1.42E-27 |  |
| *Colec12* | 9.48E-32 | -0.28640559 | 1.93E-27 |  |
| *Grk5* | 1.67E-31 | 0.440884612 | 3.41E-27 |  |
| *Bcl2a1d* | 1.94E-31 | -0.371169173 | 3.96E-27 |  |
| *Parp8* | 3.82E-31 | 0.331746409 | 7.80E-27 |  |
| *Gm26740* | 4.47E-31 | 0.265795513 | 9.11E-27 |  |
| *Stard8* | 4.64E-31 | -0.306510679 | 9.45E-27 |  |
| *Ahnak* | 6.16E-31 | -0.257665923 | 1.26E-26 |  |
| *Utrn* | 6.85E-31 | 0.254408668 | 1.40E-26 |  |
| *Icam1* | 8.27E-31 | -0.288055934 | 1.69E-26 |  |
| *Ccl24* | 8.33E-31 | -0.378763995 | 1.70E-26 |  |
| *Itpr1* | 9.50E-31 | 0.369235464 | 1.94E-26 |  |
| *Gpcpd1* | 1.66E-30 | 0.287668096 | 3.39E-26 |  |
| *Nupr1* | 3.33E-30 | -0.280811873 | 6.80E-26 |  |
| *Cd83* | 7.35E-30 | -0.369724063 | 1.50E-25 |  |
| *Tnf* | 7.83E-30 | -0.259202459 | 1.60E-25 |  |
| *Plac8* | 1.04E-29 | 0.29990634 | 2.13E-25 |  |
| *Pdia6* | 1.28E-29 | -0.264793474 | 2.60E-25 |  |
| *Wwp1* | 1.73E-29 | -0.361545949 | 3.53E-25 |  |
| *Etv6* | 2.41E-29 | 0.303664261 | 4.92E-25 |  |
| *Tanc2* | 3.10E-29 | -0.260746326 | 6.32E-25 |  |
| *Furin* | 4.27E-29 | -0.25275248 | 8.71E-25 |  |
| *Arl15* | 6.97E-29 | 0.261187284 | 1.42E-24 |  |
| *Tmem176a* | 1.28E-28 | -0.255084938 | 2.60E-24 |  |
| *Sdc4* | 2.13E-28 | -0.262840746 | 4.34E-24 |  |
| *Fos* | 2.23E-28 | -0.445880764 | 4.56E-24 |  |
| *Tuba1a* | 2.98E-28 | -0.289926394 | 6.07E-24 |  |
| *Ophn1* | 5.05E-28 | -0.251142242 | 1.03E-23 |  |
| *Vsig4* | 7.47E-28 | -0.559829576 | 1.52E-23 |  |
| *Gpr34* | 9.13E-28 | -0.260331547 | 1.86E-23 |  |
| *Bcl11a* | 1.00E-27 | 0.375681229 | 2.05E-23 |  |
| *Cstb* | 1.15E-27 | -0.363623967 | 2.34E-23 |  |
| *Ccr5* | 1.20E-27 | -0.327094227 | 2.45E-23 |  |
| *Csf1r* | 1.43E-27 | -0.266419483 | 2.91E-23 |  |
| *Pglyrp1* | 2.72E-27 | 0.381042593 | 5.55E-23 |  |
| *Zfp36* | 6.99E-27 | -0.397564602 | 1.43E-22 |  |
| *Serpinb6a* | 8.97E-27 | -0.348301591 | 1.83E-22 |  |
| *Tank* | 9.44E-27 | -0.302082745 | 1.92E-22 |  |
| *Lyz1* | 1.66E-26 | -0.723324362 | 3.39E-22 |  |
| *1700112E06Rik* | 1.82E-26 | 0.332201083 | 3.70E-22 |  |
| *Ly6a* | 3.16E-26 | -0.518971212 | 6.45E-22 |  |
| *Thbs1* | 3.36E-26 | 0.355373615 | 6.86E-22 |  |
| *Mdfic* | 3.88E-26 | -0.251025955 | 7.90E-22 |  |
| *Itsn1* | 5.61E-26 | -0.270172719 | 1.14E-21 |  |
| *Pag1* | 1.15E-25 | 0.349247829 | 2.34E-21 |  |
| *Manf* | 1.48E-25 | -0.250631609 | 3.02E-21 |  |
| *Fcgr1* | 2.09E-25 | -0.457385153 | 4.26E-21 |  |
| *Ccr2* | 2.83E-25 | 0.272245641 | 5.78E-21 |  |
| *Glul* | 3.64E-25 | -0.301068135 | 7.43E-21 |  |
| *Sepp1* | 3.66E-25 | -0.640892132 | 7.46E-21 |  |
| *Rnase4* | 1.80E-24 | -0.43115272 | 3.67E-20 |  |
| *Gria3* | 3.11E-24 | 0.296283817 | 6.34E-20 |  |
| *Pbx1* | 3.35E-24 | 0.273016831 | 6.83E-20 |  |
| *Fnip1* | 4.50E-24 | -0.255521952 | 9.18E-20 |  |
| *Gcnt2* | 5.56E-24 | 0.266146313 | 1.13E-19 |  |
| *Smpdl3a* | 7.97E-24 | 0.289180029 | 1.63E-19 |  |
| *Cnnm2* | 1.14E-23 | 0.265348697 | 2.32E-19 |  |
| *H3f3b* | 1.58E-23 | -0.253650374 | 3.23E-19 |  |
| *Egr1* | 1.62E-23 | -0.548839213 | 3.31E-19 |  |
| *Sulf2* | 4.54E-23 | -0.257640898 | 9.26E-19 |  |
| *Il1rn* | 9.60E-23 | -0.495148848 | 1.96E-18 |  |
| *Kynu* | 1.15E-22 | 0.266224769 | 2.34E-18 |  |
| *Pecam1* | 1.78E-22 | 0.292683856 | 3.63E-18 |  |
| *Aif1* | 2.47E-22 | -0.252509201 | 5.04E-18 |  |
| *Cxcl10* | 3.95E-22 | -0.381484865 | 8.05E-18 |  |
| *Satb1* | 5.20E-22 | 0.286843986 | 1.06E-17 |  |
| *Eepd1* | 1.53E-21 | 0.384644771 | 3.12E-17 |  |
| *Plekhm3* | 2.18E-21 | 0.33520017 | 4.44E-17 |  |
| *Trps1* | 2.73E-21 | 0.257845239 | 5.57E-17 |  |
| *Crip1* | 5.07E-21 | -0.274948093 | 1.03E-16 |  |
| *H2-Ab1* | 1.18E-20 | -0.430766847 | 2.40E-16 |  |
| *Abr* | 1.53E-20 | 0.336089216 | 3.12E-16 |  |
| *Nrg1* | 1.69E-20 | 0.429492394 | 3.45E-16 |  |
| *Mctp2* | 2.41E-20 | 0.616049517 | 4.91E-16 |  |
| *Marcksl1* | 3.57E-20 | -0.250447705 | 7.28E-16 |  |
| *S100a9* | 5.83E-20 | 0.583513564 | 1.19E-15 |  |
| *Clec10a* | 6.44E-20 | -0.346899435 | 1.31E-15 |  |
| *Ppm1h* | 1.00E-19 | 0.329108055 | 2.05E-15 |  |
| *Gadd45b* | 1.03E-19 | -0.265079084 | 2.10E-15 |  |
| *Cfh* | 2.63E-19 | -0.54208113 | 5.36E-15 |  |
| *Ly6g* | 3.75E-19 | 0.250398088 | 7.65E-15 |  |
| *Rsad2* | 5.99E-19 | -0.423618696 | 1.22E-14 |  |
| *Bcr* | 9.96E-19 | 0.267791203 | 2.03E-14 |  |
| *S100a8* | 2.95E-18 | 0.565387144 | 6.02E-14 |  |
| *H2-Eb1* | 7.56E-18 | -0.52572665 | 1.54E-13 |  |
| *Dach1* | 1.14E-17 | 0.317726932 | 2.33E-13 |  |
| *Camp* | 1.18E-17 | 0.94524405 | 2.42E-13 |  |
| *Ikzf1* | 3.89E-17 | 0.255382407 | 7.92E-13 |  |
| *Txnip* | 7.90E-17 | -0.313118956 | 1.61E-12 |  |
| *Arhgef3* | 1.03E-16 | -0.283600638 | 2.10E-12 |  |
| *Vcan* | 2.40E-16 | 0.352073126 | 4.90E-12 |  |
| *Bcl2a1b* | 4.10E-16 | -0.328945147 | 8.36E-12 |  |
| *Lrch1* | 4.76E-16 | 0.251350149 | 9.70E-12 |  |
| *Mmp9* | 1.87E-15 | 0.321254445 | 3.81E-11 |  |
| *Ltf* | 3.92E-15 | 0.726486585 | 7.99E-11 |  |
| *Atp2b4* | 4.64E-15 | 0.268995669 | 9.47E-11 |  |
| *Ngp* | 1.01E-14 | 0.739488838 | 2.06E-10 |  |
| *Retnlg* | 1.55E-14 | 0.461151925 | 3.17E-10 |  |
| *Man1a* | 4.89E-14 | -0.259510708 | 9.96E-10 |  |
| *H2-Aa* | 6.64E-13 | -0.404583614 | 1.35E-08 |  |
| *Fgl2* | 7.19E-13 | -0.303887094 | 1.47E-08 |  |
| *Wfdc21* | 8.80E-13 | 0.378959185 | 1.79E-08 |  |
| *Rnf213* | 1.40E-12 | -0.268324212 | 2.85E-08 |  |
| *Gm26917* | 2.47E-12 | -0.298432872 | 5.04E-08 |  |
| *Gramd1b* | 2.77E-12 | 0.26496509 | 5.66E-08 |  |
| *Ifi27l2a* | 3.18E-12 | -0.321510075 | 6.48E-08 |  |
| *Slfn4* | 8.85E-12 | -0.352868104 | 1.80E-07 |  |
| *Lcn2* | 1.01E-11 | 0.611985265 | 2.06E-07 |  |
| *Fyn* | 1.80E-11 | 0.306070365 | 3.67E-07 |  |
| *Ednrb* | 2.14E-11 | -0.279768733 | 4.37E-07 |  |
| *Cd74* | 3.58E-11 | -0.409667343 | 7.30E-07 |  |
| *Abhd17b* | 4.18E-11 | 0.322077098 | 8.53E-07 |  |
| *Serinc3* | 7.80E-10 | -0.303033223 | 1.59E-05 |  |
| *Cd209a* | 7.84E-10 | -0.274052425 | 1.60E-05 |  |
| *Adpgk* | 8.46E-10 | 0.269402785 | 1.73E-05 |  |
| *Tcf4* | 1.01E-09 | 0.363823135 | 2.06E-05 |  |
| *Zbp1* | 1.99E-09 | -0.269515549 | 4.06E-05 |  |
| *Tex2* | 3.61E-08 | 0.5013825 | 0.000735289 |  |
| *Ccl5* | 1.58E-07 | -0.359995839 | 0.003230023 |  |
| *Bach2* | 1.85E-06 | 0.29733023 | 0.037669814 |  |
| *St8sia4* | 2.05E-06 | 0.310847684 | 0.041717689 |  |
| *Lrp8* | 2.25E-06 | 0.347165463 | 0.045956529 |  |
